# Gold-catalyzed cyclization of allenyl acetal derivatives

**DOI:** 10.3762/bjoc.9.202

**Published:** 2013-08-27

**Authors:** Dhananjayan Vasu, Samir Kundlik Pawar, Rai-Shung Liu

**Affiliations:** 1 Department of Chemistry, National Tsing Hua University, Hsinchu, 30013, Taiwan (ROC)

**Keywords:** allenyl acetals, 5-alkylidenecyclopent-2-en-1-ones, cyclization, gold catalysis

## Abstract

The gold-catalyzed transformation of allenyl acetals into 5-alkylidenecyclopent-2-en-1-ones is described. The outcome of our deuterium labeling experiments supports a 1,4-hydride shift of the resulting allyl cationic intermediates because a complete deuterium transfer is observed. We tested the reaction on various acetal substrates bearing a propargyl acetate, giving 4-methoxy-5-alkylidenecyclopent-2-en-1-ones **4** via a degradation of the acetate group at the allyl cation intermediate.

## Introduction

**Scheme 1:**
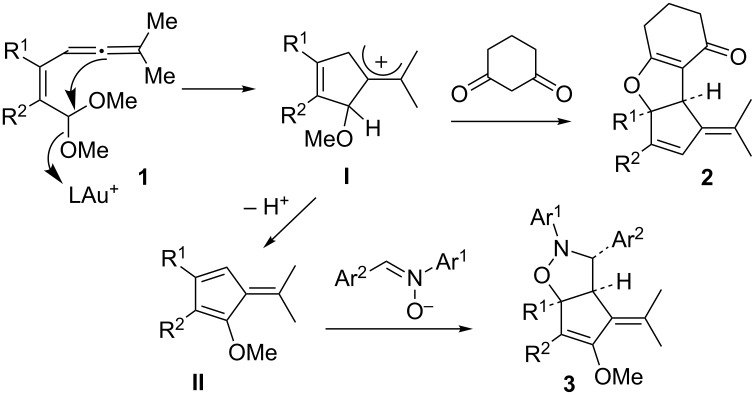
Reported cascade reactions on allenyl acetals.

Gold-catalyzed cyclization/cycloaddition reactions [1–5] are useful synthetic methods to construct complicated carbo- and oxacyclic frameworks. Such cascade reactions have been well studied on various difunctionalized molecules including oxoalkynes [6–13], oxoallenes [14], oxoalkenes [15] and allenyl acetals [16–18]. In this cascade sequence, two new rings and three chemical bonds are generated in a one-pot procedure. We previously reported gold-catalyzed reactions of allenyl acetals with suitable dipolarophiles such as 1,3-diones to chemoselectively produce the cycloaddition product **2** [17] (Scheme 1). Similar reactions with nitrones stereoselectively delivered distinct formal cycloadducts **3** [18]. We postulate that compounds **2** arise from the attack of 1,3-diones at initially generated allyl cation intermediates **I**. In the case of electrophilic nitrones, allyl cations **I** release a proton to form reactive 1-methoxyfulvenes **II** to achieve a [3 + 2]-nitrone cycloaddition. The versatility of cationic intermediates **I** encourages us to understand their behavior in the absence of a dipolarophile. This work reports gold-catalyzed intramolecular cyclizations of these allenyl acetals [19].

## Results and Discussion

We first tested the intramolecular cyclizations of allenyl acetal **1a** with PPh_3_AuCl/AgSbF_6_ (5 mol %), which was shown to be an active catalyst in the two cascade reactions, as depicted in Scheme 1 [17–18]. As shown in Table 1, the treatment of compound **1a** with this gold catalyst (5 mol %) in dichloromethane (DCM, 28 °C, 0.5 h) afforded 5-isopropylidenecyclopent-2-en-1-one derivative **4a** in 65% yield (Table 1, entry 1). With a change of the counter anion as in PPh_3_AuCl/AgOTf, the product yield increased to 89% (Table 1, entry 2). PPh_3_AuCl/AgNTf_2_ was also active to give the same product in 83% yield (Table 1, entry 3). Under the same conditions, AgOTf alone gave the desired **4a** in 48% yield (Table 1, entry 4). AuCl_3_ and PtCl_2_ enabled a complete consumption of the starting material **1a**, but the yields of compound **4a** were 51% and 30%, respectively (Table 1, entries 5 and 6).

**Table 1:** Catalyst screening over various acid catalysts.

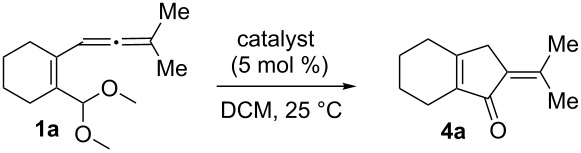			

Entry^a^	Catalyst	Time (h)	Yield (%)^b^

1	PPh_3_AuCl/AgSbF_6_	0.5	65
2	PPh_3_AuCl/AgOTf	0.5	89
3	PPh_3_AuCl/AgNTf_2_	0.5	83
4	AgOTf	2.0	48
5	AuCl_3_/CO	1.5	51
6	PtCl_2_/CO	1.5	30

^a^[**1a**] = 0.1 M. ^b^Isolated yields.

Table 2 shows the substrate scope including additional allenyl acetals **1b**–**1h**. The reactions were catalyzed by PPh_3_AuCl/AgNTf_2_ (5 mol %) in DCM. As shown in entries 1–3, this cyclization was applicable to allenyl acetals **1b**–**1d** bearing a cyclopentyl bridge. The resulting products **4b**–**4d** were produced with satisfactory yields (68–82%). We also tested the reaction on acyclic allenyl acetal **1e** (*E*/*Z* = 3:1), and afforded the desired product **4e** in 52% yield according to initial *E*-configured **1e**. The structure of compound **4e** was determined by ^1^H NMR NOE spectra. The reaction was still operable with **1f**, bearing a 1,2-disubsituted allene, giving the desired **4f** in moderate yield (49%). Its *E*-configuration was determined by NOE measurements, and assignable to other products including **4g** and **4h**. The reaction worked well with substrates bearing a different trisubstituted allenes, giving the desired cyclopentenone **4g** and **4h** in 82–83% yields.

**Table 2:** Gold-catalyzed cyclization of allenyl acetals.

Entry	Substrates^a^	Time/min	Product (yield)^b^

1	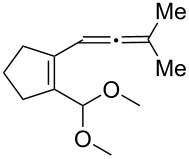 **1b**	15	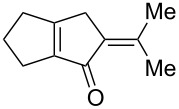 **4b** (82%)
2	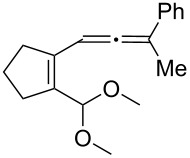 **1c**	10	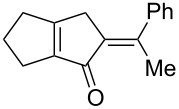 **4c** (68%)
3	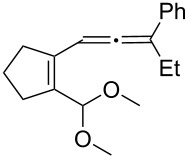 **1d**	10	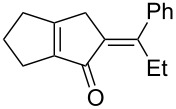 **4d** (70%)
4^c^	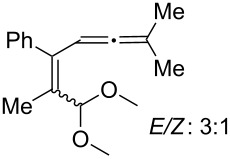 **1e**	30	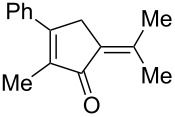 **4e** (52%)
5	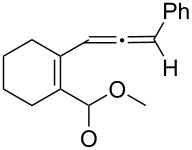 **1f**	30	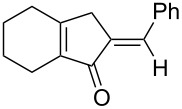 **4f** (49%)
6	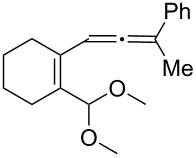 **1g**	30	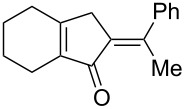 **4g** (82%)
7	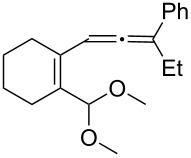 **1h**	10	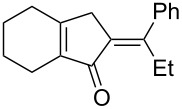 **4h** (83%)

^a^5 mol % AuClPPh_3_/AgOTf, [**1**] = 0.1 M, 25 °C, DCM. ^b^Isolated yield. ^c^10 mol % of gold catalyst.

**Scheme 2:**
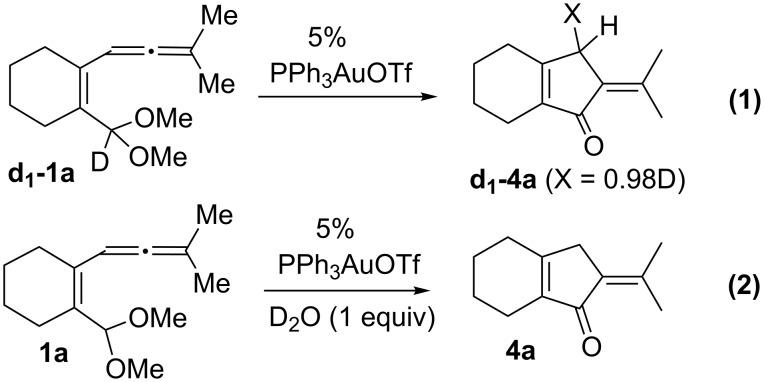
Gold-catalyzed cyclization of deuterated **d_1_-1a**.

The preceding cyclization is mechanistically interesting because it involves a cleavage of the C–H bond of the acetal group. We prepared **d_1_-1a** bearing a deuterium (>98%, Scheme 2, reaction 1) at its acetal group. The resulting product **d_1_-4a** has almost one full deuterium (X = 0.98 D) at one of the methylene protons according to DEPT ^13^C NMR analysis. In the presence of added D_2_O, undeuterated **1a** gave the product without deuterium content (Scheme 2, reaction 2). The results of these labeling experiments reveal a 1,4-hydrogen shift [20–22] in the **d_1_-1a**→**d_1_-4a** transformation.

**Scheme 3:**
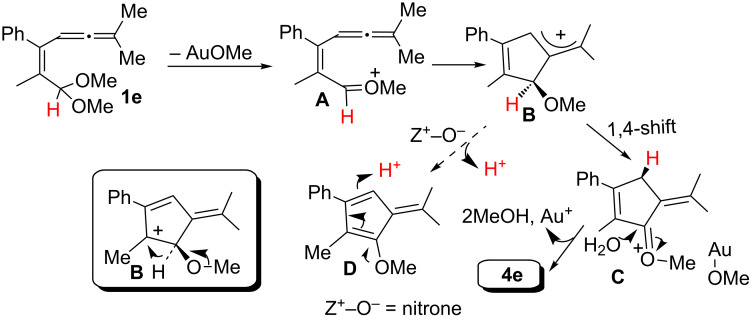
A plausible reaction mechanism.

Scheme 3 shows a plausible mechanism to rationalize the transformation of the allenyl acetal **1e** into the observed cyclopentenone **4e**. The deuterium labeling experiment of the **d_1_-1a→d_1_-4a** transformation (Scheme 2, reaction 1) indicates that one methylene proton of **4a** is derived from the original acetal group. Accordingly, we postulate a 1,4-hydride shift [21–22] for the intermediate transformation **B**→**C**. We excluded an alternative route involving the protonation of the fulvene intermediate **D** because this route would water as a proton source. The formation of the fulvene intermediate **D** from allyl cation **B** is assisted by a weak base like nitrone [18]. We envisage that a 1,2-hydrogen shift for the allyl cation **B** fails to explain a complete deuterium transfer for the **d_1_-1a→d_1_-4a** transformation because its resulting cyclopent-3-en-1-one derivative became isomerized to the final product **4a** with a loss of deuterium content.

**Scheme 4:**
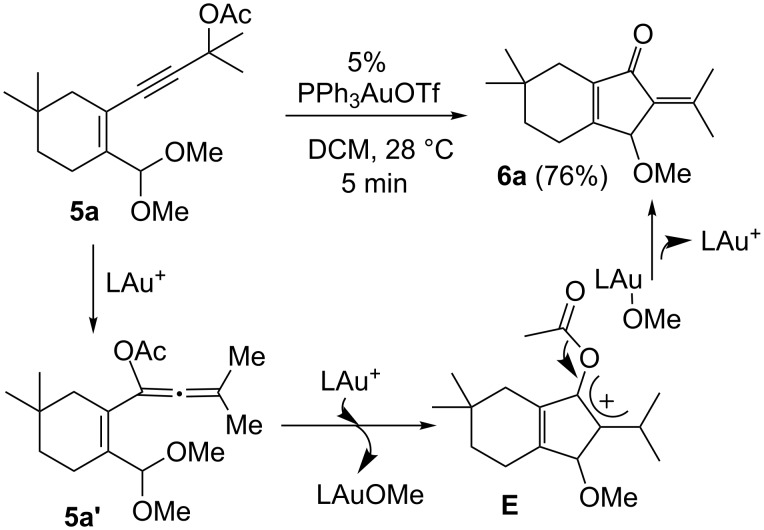
The reaction of propargyl acetate **5a**.

We also prepared the substrate **5a** bearing a propargyl acetate moiety because this functionality can be transferred to the allenyl acetate **5a’** by a gold catalyst [23–24]. As shown in Scheme 4, the treatment of species **5a** with PPh_3_AuOTf (5 mol %) in dichloromethane (28 °C, 5 min) gave 4-methoxy-5-isopropylidenecyclopent-2-en-1-one **6a** in 76% yield. The structure of compound **6a** was determined by an X-ray diffraction study (crystallographic data are provided in Supporting Information File 1). Formation of this product is postulated to arise from the attack of the methoxy anion at the acetyl group of the corresponding allyl cation **E**, a process not involving a 1,4-hydride shift. This alternative pathway highlights the diversified mechanism of such oxidative cyclizations.

We prepared the additional substrates **5b**–**5g** bearing an acetate group to examine the scope of the reaction, results are shown in Table 3. This gold-catalyzed cyclization was applicable to compound **5b** bearing a cyclopentyl bridge, giving the desired **6b** in 96% yield. The reaction worked also with **5c** and **5d** bearing a cyclohexyl bridge, delivering the desired products **6c** and **6d** in 78% and 72% yields, respectively (Table 3, entries 2 and 3). We tested the reaction with the benzenoid substrates **5e**–**5g**, giving the corresponding enones **6e**–**6g** in 63–78% yields.

**Table 3:** Gold-catalyzed carbocyclization of propargylic esters.

Entry	Substrates^a^	Time/min	Product (yield)^b^

1	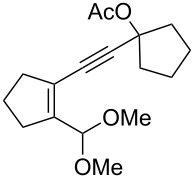 **5b**	5	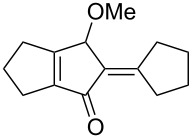 **6b** (96%)
2	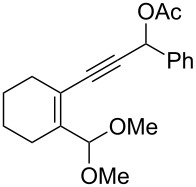 **5c**	5	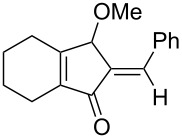 **6c** (78%)
3	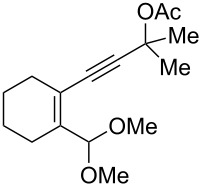 **5d**	5	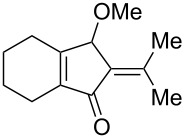 **6d** (72%)
4	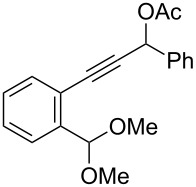 **5e**	10	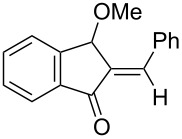 **6e** (78%)
5	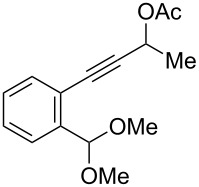 **5f**	10	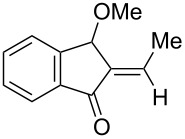 **6f** (68%)
6	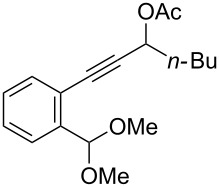 **5g**	10	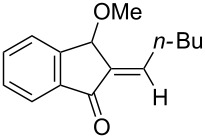 **6g** (63%)

^a^5 mol % AuClPPh_3_/ AgOTf, [**5**] = 0.1 M, 25 °C, DCM. ^b^Isolated yield.

## Conclusion

In summary, we report a gold-catalyzed transformation of allenyl acetals **1** into 5-alkylidenecyclopent-2-en-1-ones **4**. Our deuterium labeling experiments support a 1,4-hydride shift for the resulting allyl cation because of a complete deuterium transfer. This observation excludes the pathway involving the protonation of a 1-methoxyfulvene species. We tested the reactions of acetal substrates **5** bearing a propargyl acetate to afford 4-methoxy-5-alkylidenecyclopent-2-en-1-ones **6**. The formation mechanism involves a degradation of the acetate group at the corresponding allyl cation.

## Experimental

### General procedure for the gold-catalyzed carbocyclization

**General procedure for the the gold(I)-catalyzed carbocyclization of vinylallenyl acetal:** A two-necked flask was charged with chloro(triphenylphosphine)gold(I) (11.1 mg, 0.022 mmol) and silver triflate (5.8 mg, 0.022 mmol), and to this mixture CH_2_Cl_2_ (2.0 mL) was added. The resulting solution was stirred at room temperature for 10 min. To this mixture a solution of vinylallenyl acetal **1a** (100 mg, 0.45 mmol) in CH_2_Cl_2_ (2.5 mL) was added dropwise, and the mixture was kept stirring at 25 °C for 30 min before it was filtered over a short silica bed. The solvent was evaporated under reduced pressure. The crude product was eluted through a short silica column (3% ethyl acetate in hexane) to afford the desired ketone **4a** (70.6 mg, 0.40 mmol, 89%) as a pale yellow oil.

**General procedure for the gold(I)-catalyzed carbocyclization of propargylic ester acetals:** Chloro(triphenylphosphine)gold(I) (8.0 mg, 0.016 mmol) and silver triflate (4.2 mg, 0.016 mmol) were added to a dried Schlenk tube under an N_2_ atmosphere, and freshly distilled CH_2_Cl_2_ (1.0 mL) was introduced by a syringe. The resulting mixture was stirred at room temperature for 10 minutes before the addition of propargylic ester acetal **5a** (100 mg, 0.32 mmol) in CH_2_Cl_2_ (2.2 mL). The reaction mixture was stirred for additional 5 minutes at 25 °C. After the completion of reaction, the brown suspension was filtered through a short bed of silica gel. The solvent was removed under reduced pressure. The crude product was purified by flash chromatography to afford the desired ketone **6a** (58 mg, 0.25 mmol, 76%) as a dark yellow oil.

## Supporting Information

File 1 Experimental details.
