# Supplementary material for: Gold-catalyzed cyclization of allenyl acetal derivatives
Source: Beilstein J Org Chem. 2013 Aug 27;9:1751–6. doi: 10.3762/bjoc.9.202 (PMC3778408; doi:10.3762/bjoc.9.202)
Supplement: File 1 — Experimental details. [file Beilstein_J_Org_Chem-09-1751-s001.pdf]

Supporting Information

for

Gold-catalyzed cyclization of allenyl acetal derivatives

Dhananjayan Vasu, Samir Kundlik Pawar and Rai-Shung Liu*

Address: Department of Chemistry, National Tsing Hua University, Hsinchu, 30013, Taiwan (ROC)

Email: Rai-Shung Liu - rsliu@mx.nthu.edu.tw

* Corresponding author

Experimental details

Table of Contents:

(I) Representative synthetic procedures--S2

(II) General procedure for the gold(I)-catalyzed carbocyclization-----------------------S6

(III) Spectral data of compounds (**1a to 6g**) ---S7

(IV) ^{1}H and ^{13}C NMR spectra of compounds (**1a to 6g**) ----------------------------------S20

(V) ^{1}H NMR NOE spectra of compounds **4c, 4e, and 4f** --------------------------------S82

(VI) X-ray structure of compound **6a**--S92

(I) Representative synthetic procedures:

(a) General procedure:

Unless otherwise noted, all the reactions were performed in oven-dried glassware under nitrogen atmosphere with freshly distilled solvents. The THF was dried with sodium/benzophenone and distilled before use. *N*,*N*-dimethylformamide (DMF) and dichloromethane (DCM) were distilled from CaH_2 under nitrogen. DMF and triethylamine (Et_3N) were stored over 4 Å molecular sieves prior to use. All other commercial reagents were used without further purification. ^{1}H NMR and ^{13}C NMR spectra were recorded on a Varian 400, Bruker 400 and a Bruker 600 MHz spectrometer using chloroform-*d* ($CDCl_3$) and benzene-d_6 (C_6D_6) as internal standard.

(b) Typical procedure for the synthesis of 1-(dimethoxymethyl)-2-(3-methylbuta-1,2-dienyl)cyclohex-1-ene (1a). [1,2]

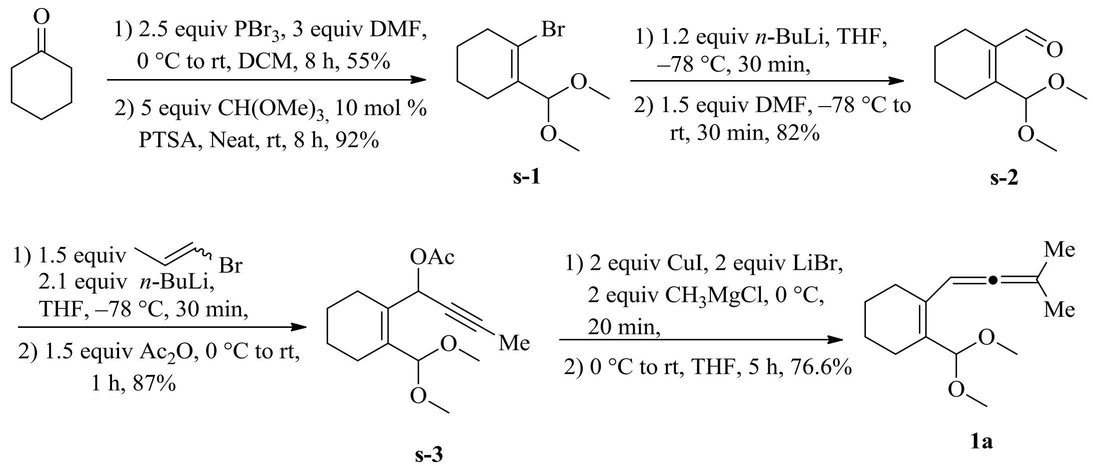

Scheme S1:

(b-1) Synthesis of 1-bromo-2-(dimethoxymethyl)cyclohex-1-ene (s-1). [1]

To dry DMF (11.8 mL, 152.9 mmol) in DCM (100 mL) was slowly added PBr_3 (12.0 mL, 127.5 mmol) at 0 °C and the mixture was stirred for 1 h at this temperature before the addition of cyclohexanone (5 g, 51.0 mmol) in dry DCM. The resulting solution was stirred for 8 h at room temperature. After completion of the reaction, the residue was carefully and slowly added to crushed ice and neutralized with saturated $NaHCO_3$ solution and extracted with EtOAc (3 × 50

mL). The combined organic extracts were initially washed with saturated aqueous $NaHCO_3$, followed by water and brine. The organic phase was dried over $MgSO_4$, filtered, and concentrated in vacuo to afford the crude bromocyclohex-1-enecarbaldehyde (5.30 g, 28.0 mmol, 55%) as yellow oil. To this yellow oil, 15.3 mL (140.2 mmol) of trimethyl orthoformate and PTSA (0.48 g, 2.80 mmol) were added and stirred at 25–30 °C until complete consumption of the aldehyde (8 h, TLC). After completion of the reaction, the mixture was diluted with hexane and neutralized by using saturated aqueous $NaHCO_3$ solution. The resulting mixture was then extracted with hexane (2 × 20 mL). The combined organic layer was dried over anhydrous Na_2SO_4 and concentrated under reduced pressure to afford the crude product (**s-1**) (6.06 g, 25.8 mmol, 92%) as pale yellow oil that was used for the next step without further purification.

(b-2) Synthesis of 2-(dimethoxymethyl)cyclohex-1-enecarbaldehyde (s-2).

To a THF (100 mL) solution of crude compound (**s-1**) (6.06 g, 25.8 mmol) was added *n*-butyllithium (12.4 mL, 30.9 mmol, 2.5 M) at −78 °C, and the mixtures were stirred for 30 min. The reaction was quenched by adding dried DMF (2.99 mL, 38.7 mmol), and the resulting mixture was allowed to reach room temperature for another 30 min. The resulting solution was partitioned between 50 mL hexane and saturated Na_2CO_3 (aq) (1:1, v/v), and the aqueous layer was extracted with hexane (2 × 30 mL). The combined organic layer was dried over anhydrous Na_2SO_4 and concentrated under reduced pressure. The residue was eluted through a triethylamine-pretreated silica gel column to give compound (**s-2**) (3.89 g, 21.1 mmol, 82%) as pale yellow oil.

(b-3) Synthesis of 1-(2-(dimethoxymethyl)cyclohex-1-enyl)but-2-ynyl acetate (s-3).

A THF (100 mL) solution of 1-bromo-1-propene (2.71 mL, 31.7 mmol) was cooled to −78 °C before addition of *n*-butyllithium (17.7 mL, 44.4 mmol, 2.5 M). The reaction was kept at −78 °C for 30 min, and to this solution was added a THF (5 mL) solution of compound (**s-2**) (3.89 g, 21.1 mmol) and the reaction was allowed to reach room temperature for 30 min, followed by addition of acetic anhydride (2.99 mL, 31.7 mmol) at 0 °C. The resulting mixture was stirred at room temperature for another 1 h before it was quenched with saturated aqueous Na_2CO_3. The aqueous solution was extracted with hexane (30 mL × 3). The combined organic layer was dried

over anhydrous Na_2SO_4 and concentrated in vacuo. The crude product **s-3** (4.89 g, 18.4 mmol, 87%) was obtained as pale yellow oil and used for next step without further purification.

(b-4) Synthesis of 1-(dimethoxymethyl)-2-(3-methylbuta-1,2-dienyl)cyclohex-1-ene (1a). [2] To a well stirred mixture of lithium bromide (3.19 g, 36.8 mmol) and copper iodide (6.98 g, 36.8 mmol) in THF (100 mL) at 0 °C was added methylmagnesium chloride (12.3 mL, 36.8 mmol, 3 M), and the solution was stirred for 20 min at 0 °C. The propargylic ester (**s-3**) (4.89 g, 18.4 mmol) in THF (5 mL) was added dropwise and the resulting mixture was slowly warmed to rt and stirred for an additional 5 h. At the completion of the reaction indicated by TLC, the mixture was poured into a saturated aqueous solution of ammonium chloride and partitioned between hexane/saturated Na_2CO_3 (aq) (1:2, v/v). The aqueous layer was extracted with hexane (2 × 100 mL). The combined organic layer was dried over anhydrous Na_2SO_4 and evaporated under reduced pressure. The crude product was purified on a triethylamine-pretreated silica column to afford desired vinylallenyl acetal (**1a**) (3.35 g, 14.1 mmol, 76.6%) as a yellow oil.

(c) Synthesis of 4-(2-(dimethoxymethyl)-5,5-dimethylcyclohex-1-enyl)-2-methylbut-3-yn-2-yl acetate (5a):

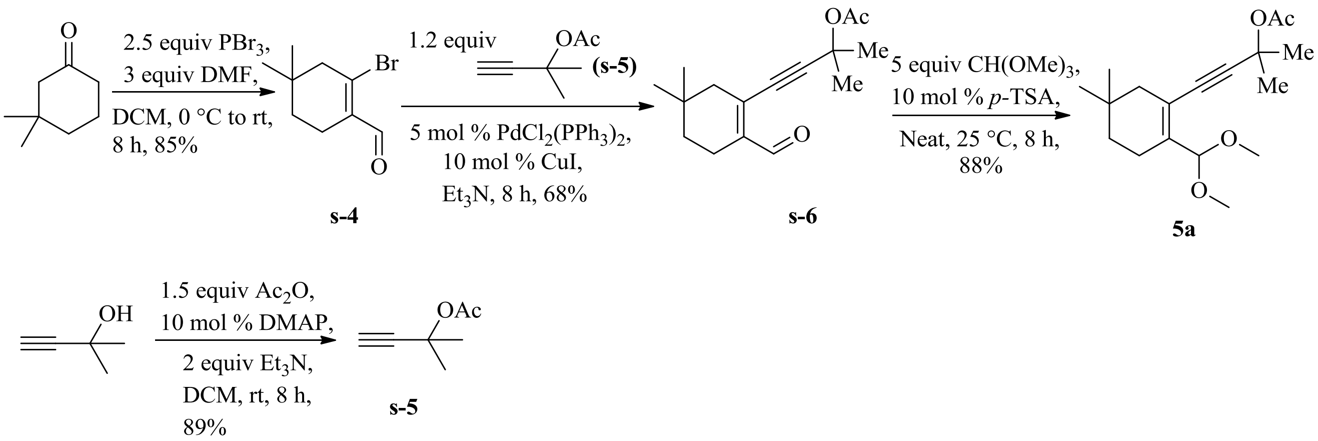

Scheme S2:

(c-1) Synthesis of 2-bromo-4,4-dimethylcyclohex-1-enecarbaldehyde (s-4). [1]

To dry DMF (9.20 mL, 118.9 mmol) in chloroform (100 mL) was slowly added PBr_3 (9.31 mL, 99.0 mmol) at 0 °C and the mixture was stirred for 1 h at this temperature before addition of 3,3-dimethylcyclohexanone (5 g, 39.6 mmol) in dry DCM. The resulting solution was stirred for 8 h at room temperature. After completion of the reaction, the residue was carefully added to crushed ice and neutralized with saturated $NaHCO_3$ solution and extracted with EtOAc (3 × 50 mL). The combine organic extracts were initially washed with saturated aqueous $NaHCO_3$ followed by water and brine. The organic phase was dried over $MgSO_4$, filtered, and concentrated in vacuo. The crude residue was purified by flash chromatography (hexane/ethyl acetate, 9:1) to afford 2-bromo-4,4-dimethylcyclohex-1-enecarbaldehyde (**s-4**) (7.31 g, 85%, 33.7 mmol) as a yellow oil.

(c-2) Synthesis of 2-methylbut-3-yn-2-yl acetate (s-5).

To a DCM (200 mL) solution of 2-methylbut-3-yn-2-ol (10 g, 118.9 mmol) was added Ac_2O (18.2 g, 178.4 mmol), Et_3N (24.1 g, 237.8 mmol) and several crystals of DMAP (1.45 g, 11.9 mmol). The mixture was stirred overnight at room temperature. Upon completion of the reaction as indicated by TLC, the solution was then quenched with saturated ammonium chloride (aq). The resulting mixture was partitioned between 200 mL hexane and saturated Na_2CO_3 (aq) (1:1, v/v), and the aqueous layer was extracted with hexane (2 × 50 mL). The combined organic layer was dried over anhydrous Na_2SO_4 and concentrated under reduced pressure. The crude product was eluted through a triethylamine-pretreated silica gel column to afford compound (**s-5**) (13.4 g, 89%, 106.2 mmol) as pale yellow oil.

(c-3) Synthesis of 4-(2-formyl-5,5-dimethylcyclohex-1-enyl)-2-methylbut-3-yn-2-yl acetate (s-6).

To a suspension of $PdCl_2(PPh_3)_2$ (1.18 g, 1.68 mmol) and CuI (0.641 g, 3.37 mmol) in 50 mL triethylamine at room temperature compound (**s-4**) (7.31 g, 33.7 mmol) was added and stirred for 15 min. To this solution, 2-methylbut-3-yn-2-yl acetate (**s-5**) (5.10 g, 40.4 mmol) was added dropwise using a standard syringe. After stirring the mixture at room temperature for 8 h the resulting solution was filtered through a small celite bed and washed three times with ethyl acetate. It was then concentrated and purified by column chromatography, which yielded the product **s-6** (6.00 g, 68%, 22.9 mmol) as yellow oil.

(c-4) Synthesis of 4-(2-(dimethoxymethyl)-5,5-dimethylcyclohex-1-enyl)-2-methylbut-3-yn-2-yl acetate (5a). [3]

A mixture of aldehyde (**s-6**) (6.00 g, 22.9 mmol), *p*-TsOH monohydrate (0.394 g, 2.29 mmol) and trimethyl orthoformate (12.53 mL, 114.5 mmol) was stirred at 25 °C for 8 h and then concentrated in vacuo. The residue was diluted with hexane, washed with saturated $NaHCO_3$ solution, water, and brine. The organic phase was dried over Na_2SO_4, filtered, and concentrated under reduced pressure. The crude residue was purified by column chromatography with

pretreated triethylamine silica gel to afford the desired propargylic ester acetal (**5a**) (6.22 g, 20.2 mmol, 88%) as yellow oil.

References:

1. Vasu, D.; Liu, R.-S. *Chem. Eur. J.* **2012**, *18*, 13638. DOI:10.1002/chem.201201777

2. Macdonald, T. L.; Reagan D. R., *J. Org. Chem.* **1980**, *45*, 4740. DOI: 10.1021/jo01311a037

3. a) Kumar, R.; Kumar D.; Chakraborti, A. K., *Synthesis*. **2007**, *2*, 299. DOI: 10.1055/s-2006-958948; b) Chakraborti, A. K.; Gulhane, R., *Chem. Commun*. **2003**, 1896. DOI: 10.1039/B304178F

(II) General procedure for gold-catalyzed carbocyclization:

(a) General procedure for the gold (I)-catalyzed carbocyclization of vinylallenyl acetal:

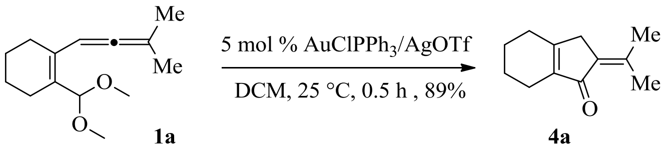

1a **4a**

A two-necked flask was charged with chloro(triphenylphosphine)gold(I) (11.1 mg, 0.022 mmol) and silver triflate (5.8 mg, 0.022 mmol), and CH_2Cl_2 (2.0 mL) was added. The resulting mixture was stirred at room temperature for 10 min. To this mixture was added a CH_2Cl_2 (2.5 mL) solution of vinylallenyl acetal (**1a**) (100 mg, 0.45 mmol) dropwise and the mixture was kept stirring at 25 °C for 30 min before it was filtered over a short silica bed. The solvent was evaporated under reduced pressure. The crude product was eluted through a short silica column (3% ethyl acetate in hexane) affords the desired ketone **4a** (70.6 mg, 0.40 mmol, 89%) as a pale yellow oil.

(b) General procedure for the gold(I)-catalyzed carbocyclization of propargylic ester acetals:

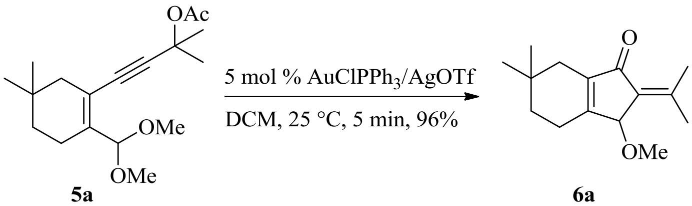

5a **6a**

Chloro(triphenylphosphine)gold (I) (8.0 mg, 0.016 mmol) and silver triflate (4.2 mg, 0.016 mmol) was added to a dried Schlenk tube under an N_2 atmosphere, and then freshly distilled DCM (1.0 mL) was introduced by a syringe. The resulting mixture was stirred at room temperature for 10 minutes before the addition of propargylic ester acetal (**5a**) (100 mg, 0.32 mmol) in DCM (2.2 mL). The reaction mixture was stirred for another 5 minutes at 25 ºC (reaction monitored by TLC). After completion of the reaction, the brown suspension was filtered through a short bed of silica gel and the solvent was removed under reduced pressure. The crude product was purified by flash chromatography to afford the desired ketone **6a** (58 mg, 0.25 mmol, 76%) as dark yellow oil.

(III) Spectral data of Compounds 1a to 6g:

Spectral data for 1-(dimethoxymethyl)-2-(3-methylbuta-1,2-dienyl)cyclohex-1-ene (1a). [1]

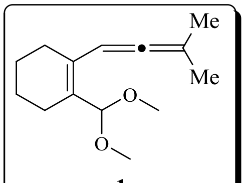

1a

Compound **1a** was prepared according to the known literature procedure [1] reported by our laboratory.

Spectral data for 1-(dimethoxymethyl)-2-(3-methylbuta-1,2-dienyl)cyclopent-1-ene (1b).

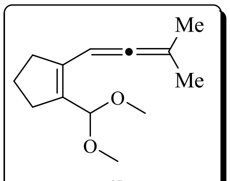

1b

Yellow oil, IR (neat, cm^{-1}): 2962 (s), 1649 (w), 1441 (m), 1086 (s); ^{1}H NMR (400 MHz, CD_2Cl_2): δ 6.15–6.12 (m, 1H), 5.02 (s, 1H), 3.28 (s, 6H), 2.47–2.38 (m, 4H), 1.83–1.77 (m, 2H), 1.75 (s, 3H), 1.73 (s, 3H); ^{13}C NMR (100 MHz, CD_2Cl_2): δ 205.3, 137.2, 134.7, 101.5, 97.0, 87.2, 53.6 (2 x OCH_3), 35.2, 33.6, 21.9, 20.6 (2 x CH_3); HRMS calcd for $C_{13}H_{20}O_2$: 208.1463, found: 208.1462.

Spectral data for (4-(2-(dimethoxymethyl)cyclopent-1-enyl)buta-2,3-dien-2-yl)benzene (1c).

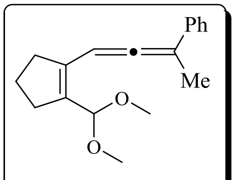

1c

Yellow oil, IR (neat, cm^{-1}): 2978 (s), 1638 (w), 1464 (m), 1064 (s), 867 (s), 692 (s); ^{1}H NMR (400 MHz, $CDCl_3$): δ 7.41–7.38 (m, 2H), 7.33–7.29 (m, 2H), 7.22–7.18 (m, 1H), 6.66 (q, *J* = 2.8 Hz, 1H), 5.12 (s, 1H), 3.37 (s, 3H), 3.36 (s, 3H), 2.56–2.52 (m, 2H), 2.50–2.45 (m, 2H), 2.14 (d, *J* = 2.8 Hz, 3H), 1.87–1.80 (m, 2H); ^{13}C NMR (100 MHz, $CDCl_3$): δ 208.4, 136.7, 135.8, 135.5, 128.3, 126.6, 125.7, 102.1, 101.2, 90.8, 53.6, 53.5, 34.8, 33.3, 21.4, 17.0; HRMS calcd for $C_{18}H_{22}O_2$: 270.1620, found: 270.1618.

Spectral data for (1-(2-(dimethoxymethyl)cyclopent-1-enyl)penta-1,2-dien-3-yl)benzene (1d).

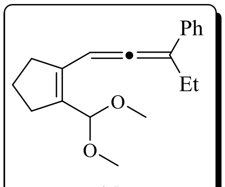

1d

Pale yellow oil, IR (neat, cm^{-1}): 2978 (s), 1638 (w), 1464 (m), 1064 (s), 878 (s), 686 (s); ^{1}H NMR (400 MHz, C_6D_6): δ 7.44–7.42 (m, 2H), 7.19–7.15 (m, 2H), 7.07–7.03 (m, 1H), 6.99 (t, *J* =

3.2 Hz, 1H), 5.10 (s, 1H), 3.18 (s, 3H), 3.17 (s, 3H), 2.67–2.62 (m, 2H), 2.51–2.46 (m, 2H), 2.38–2.31 (m, 2H), 1.72–1.66 (m, 2H), 1.10 (t, *J* = 7.4 Hz, 3H); ^{13}C NMR (100 MHz, C_6D_6): δ 208.2, 137.1, 136.5, 135.4, 128.7, 127.0, 126.4, 109.5, 101.4, 93.7, 52.8, 52.7, 35.3, 34.5, 23.5, 21.8, 12.9; HRMS calcd for $C_{19}H_{24}O_2$: 284.1776, found: 284.1773.

Spectral data for (1,1-dimethoxy-2,6-dimethylhepta-2,4,5-trien-3-yl)benzene (1e).

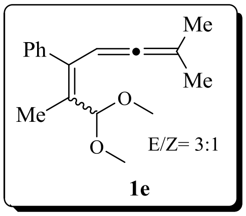

Pale yellow oil, IR (neat, cm^{-1}): 1647 (w), 1453 (m), 1048 (s), 869 (s), 689 (s); ^{1}H NMR (400 MHz, $CDCl_3$): δ 7.28–7.24 (m, 2H), 7.21–7.19 (m, 5H), 7.08–7.04 (m, 3H), 6.28–6.25 (m, 1H), 6.15–6.12 (m, 1H), 5.24 (s, 1H), 4.40 (s, 1H), 3.41 (s, 6H), 3.17 (s, 6H), 1.85 (s, 3H), 1.42 (s, 3H), 1.41 (s, 3H), 1.40 (s, 3H); ^{13}C NMR (100 MHz, $CDCl_3$): δ 207.2, 206.6, 140.9, 139.8, 137.3, 137.0, 129.5, 129.3, 128.9 (1 x CH, 1 x C), 127.5, 127.3, 126.5, 126.4, 105.3, 102.8, 97.4, 97.2, 92.4, 91.0, 54.6 (2 x OCH_3), 54.3 (2 x OCH_3), 19.7 (2 x CH_3), 19.6 (2 x CH_3), 14.5, 11.0; HRMS calcd for $C_{17}H_{22}O_2$: 258.3554, found: 258.3552.

Spectral data for (3-(2-(dimethoxymethyl)cyclohex-1-enyl)propa-1,2-dienyl)benzene (1f).

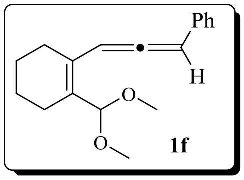

Colourless oil, IR (neat, cm^{-1}): 2957 (s), 1633 (w), 1465 (m), 1063 (s), 872 (s), 681 (s); ^{1}H NMR (400 MHz, C_6D_6): δ 7.33–7.30 (m, 2H), 7.20–7.15 (m, 2H), 7.08–7.04 (m, 1H), 7.00 (d, *J* = 6.4 Hz, 1H), 6.41 (d, *J* = 6.4 Hz, 1H), 5.13 (s, 1H), 3.25 (s, 3H), 3.21 (s, 3H), 2.43–2.40 (m, 2H), 2.33–2.28 (m, 1H), 2.23–2.12 (m, 1H), 1.55–1.47 (m, 4H); ^{13}C NMR (100 MHz, C_6D_6): δ 208.4, 134.8, 133.7, 129.2, 129.0, 127.3, 127.1, 102.7, 98.0, 96.8, 53.7, 53.4, 27.7, 25.3, 22.8, 22.7; HRMS calcd for $C_{18}H_{22}O_2$: 270.1620, found: 270.1621.

Spectral data for (4-(2-(dimethoxymethyl)cyclohex-1-enyl)buta-2,3-dien-2-yl)benzene (1g).

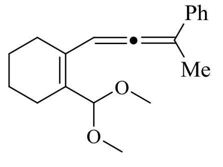

1g

Yellow oil, IR (neat, cm^{-1}): 2934 (s), 1647 (w), 1471 (m), 1085 (s), 869 (s), 687 (s); ^1H NMR (400 MHz, $CDCl_3$): δ 7.42–7.40 (m, 2H), 7.34–7.30 (m, 2H), 7.23–7.19 (m, 1H), 6.69–6.67 (m, 1H), 5.16 (s, 1H), 3.39 (s, 3H), 3.38 (s, 3H), 2.21–2.14 (m, 7H), 1.62–1.61 (m, 4H); ^{13}C NMR (100 MHz, $CDCl_3$): δ 206.9, 136.8, 131.8, 130.5, 128.3, 126.6, 125.6, 103.2, 102.8, 94.1, 54.4, 54.3, 27.4, 24.1, 22.4, 22.3, 17.0; HRMS calcd for $C_{19}H_{24}O_2$: 284.1776, found: 284.1779.

Spectral data for (1-(2-(dimethoxymethyl)cyclohex-1-enyl)penta-1,2-dien-3-yl)benzene (1h).

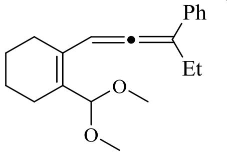

1h

Yellow oil, IR (neat, cm^{-1}): 2945 (s), 1638 (w), 1442 (m), 1048 (s), 872 (s), 691 (s); ^1H NMR (400 MHz, C_6D_6): δ 7.43–7.41 (m, 2H), 7.17–7.13 (m, 2H), 7.05–7.01 (m, 1H), 6.93 (t, J = 3.2 Hz, 1H), 5.13 (s, 1H), 3.18 (s, 3H), 3.16 (s, 3H), 2.39–2.31 (m, 4H), 2.23–2.17 (m, 2H), 1.51–1.39 (m, 4H), 1.08 (t, J = 7.3 Hz, 3H); ^{13}C NMR (100 MHz, C_6D_6): δ 206.6, 137.1, 132.9, 129.9, 128.7, 127.0, 126.3, 110.6, 102.7, 96.9, 53.7, 53.5, 27.8, 25.1, 23.5, 22.9, 22.8, 12.9; HRMS calcd for $C_{20}H_{26}O_2$: 298.1933, found: 298.1931.

Spectral data for 1-(dimethoxymethyl)-2-(3-methylbuta-1,2-dienyl)cyclohex-1-ene (d_1-1a).

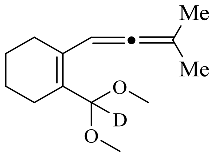

d_1-1a

Pale yellow oil, IR (neat, cm^{-1}): 2932 (s), 1645 (w), 1439 (m), 1012 (s); ^1H NMR (400 MHz, $CDCl_3$): δ 6.12–6.09 (m, 1H), 3.29 (s, 6H), 2.14–1.99 (m, 4H), 1.68 (s, 3H), 1.67 (s, 3H), 1.56–1.53 (m, 4H); ^{13}C NMR (100 MHz, $CDCl_3$): δ 203.5, 131.4, 130.4, 97.6, 90.0, 54.2 (2 x OCH_3), 27.2, 23.4, 22.5, 22.4, 20.4 (2 x CH_3); HRMS calcd for $C_{14}H_{21}DO_2$: 223.1683, found: 223.1689.

Spectral data for 4-(2-(dimethoxymethyl)-5,5-dimethylcyclohex-1-enyl)-2-methylbut-3-yn-2-yl acetate (5a).

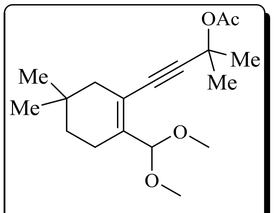

5a

Pale yellow oil, IR (neat, cm^{-1}): 2961 (s), 1721 (s), 1642 (w), 1468 (m), 1048 (s); ^1H NMR (400 MHz, $CDCl_3$): δ 5.22 (s, 1H), 3.36 (s, 6H), 2.13–2.10 (m, 2H), 1.97 (s, 3H), 1.92–1.91 (m, 2H), 1.63 (s, 6H), 1.31 (t, J = 6.5 Hz, 2H), 0.86 (s, 6H); ^{13}C NMR (100 MHz, $CDCl_3$): δ 169.1, 141.0, 118.1, 105.2, 93.0, 83.0, 72.3, 55.2 (2 x OCH_3), 43.4, 34.3, 29.0 (2 x CH_3), 28.7, 27.9 (2 x CH_3), 21.9, 19.6; HRMS calcd for $C_{18}H_{28}O_4$: 308.1988, found: 308.1984.

Spectral data for 1-((2-(dimethoxymethyl)cyclopent-1-enyl)ethynyl)cyclopentyl acetate (5b).

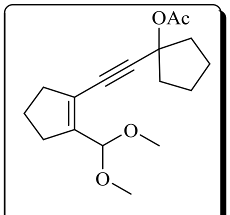

5b

Yellow oil, IR (neat, cm^{-1}): 2932 (s), 1728 (s), 1631 (w), 1427 (m), 1042 (s); ^1H NMR (400 MHz, C_6D_6): δ 5.44 (s, 1H), 3.37 (s, 6H), 2.63–2.58 (m, 2H), 2.43–2.38 (m, 2H), 2.30–2.15 (m, 4H), 1.65 (s, 3H), 1.61–1.50 (m, 6H); ^{13}C NMR (100 MHz, C_6D_6): δ 168.7, 149.0, 122.3, 102.5, 95.3, 81.1, 80.9, 54.6 (2 x OCH_3), 40.7 (2 x CH_2), 37.0, 31.4, 23.5 (2 x CH_2), 22.6, 21.3; HRMS calcd for $C_{17}H_{24}O_4$: 292.1675, found: 292.1670.

Spectral data for 3-(2-(dimethoxymethyl)cyclohex-1-enyl)-1-phenylprop-2-ynyl acetate (5c).

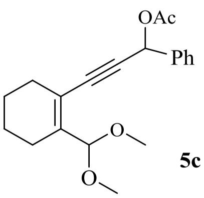

5c

Yellow oil, IR (neat, cm^{-1}): 2946 (s), 1730 (s), 1635 (w), 1442 (m), 1063 (s), 887 (s), 691 (s); ^{1}H NMR (400 MHz, $CDCl_3$): δ 7.52–7.49 (m, 2H), 7.39 –7.31 (m, 3H), 6.56 (s, 1H), 5.12 (s, 1H), 3.33 (s, 3H), 3.30 (s, 3H), 2.20–2.19 (m, 2H), 2.12–2.11 (m, 2H), 2.08 (s, 3H), 1.60–1.57 (m, 4H); ^{13}C NMR (100 MHz, $CDCl_3$): δ 169.7, 143.5, 137.2, 128.8, 128.6, 127.6, 118.7, 105.2, 89.0, 86.1, 66.2, 55.2 (2 x OCH_3), 29.8, 22.0, 21.9, 21.4, 21.0; HRMS calcd for $C_{20}H_{24}O_4$: 328.1675, found: 328.1673.

Spectral data for 4-(2-(dimethoxymethyl)cyclohex-1-enyl)-2-methylbut-3-yn-2-yl acetate (5d).

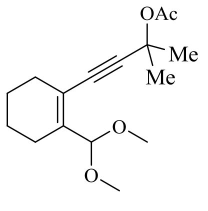

5d

Yellow oil, IR (neat, cm^{-1}): 2954 (s), 1722 (s), 1628 (w), 1463 (m), 1054 (s); ^{1}H NMR (400 MHz, $CDCl_3$): δ 5.19 (s, 1H), 3.33 (s, 6H), 2.11–2.09 (m, 2H), 2.06–2.04 (m, 2H), 1.94 (s, 3H), 1.60 (s, 6H), 1.54–1.50 (m, 4H); ^{13}C NMR (100 MHz, $CDCl_3$): δ 169.0, 142.3, 118.9, 105.3, 93.3, 82.7, 72.2, 55.1 (2 x OCH_3), 29.8, 28.9 (2 x CH_3), 22.1, 21.8, 21.7, 21.5; HRMS calcd for $C_{16}H_{24}O_2$: 280.1675, found: 280.1678.

Spectral data for 3-(2-(dimethoxymethyl)phenyl)-1-phenylprop-2-ynyl acetate (5e).

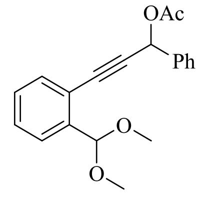

5e

Yellow oil, IR (neat, cm^{-1}): 3059 (m), 1735 (s), 1636 (w), 1438 (m), 1051 (s), 896 (s), 689 (s); ^{1}H NMR (400 MHz, $CDCl_3$): δ 7.62 (d, J = 7.4 Hz, 2H), 7.57 (d, J = 7.8 Hz, 1H), 7.48 (d, J = 7.8 Hz, 1H), 7.42–7.34 (m, 4H), 7.29–7.25 (m, 1H), 6.70 (s, 1H), 5.58 (s, 1H), 3.35 (s, 3H), 3.34 (s, 3H), 2.12 (s, 3H); ^{13}C NMR (100 MHz, $CDCl_3$): δ 169.6, 140.4, 137.2, 132.5, 129.0, 128.9, 128.7, 128.4, 127.8, 126.2, 121.0, 102.5, 90.3, 84.8, 66.2, 54.2, 54.1, 20.9; HRMS calcd for $C_{20}H_{20}O_4$: 324.1362, found: 324.1366.

Spectral data for 4-(2-(dimethoxymethyl)phenyl)but-3-yn-2-yl acetate (5f).

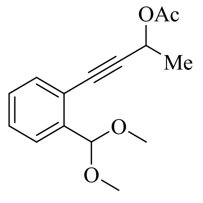

5f

Yellow oil, IR (neat, cm^{-1}): 3068 (m), 1730 (s), 1628 (w), 1459 (m), 1060 (s), 901 (s), 698 (s); ^{1}H NMR (400 MHz, $CDCl_3$): δ 7.54 (dd, *J* = 7.6, 1.1 Hz, 1H), 7.41 (dd, *J* = 7.6, 1.1 Hz, 1H), 7.31 (td, *J* = 7.6, 1.1 Hz, 1H), 7.23 (td, *J* = 7.6, 1.1 Hz, 1H), 5.66 (q, *J* = 6.7 Hz, 1H), 5.57 (s, 1H), 3.38 (s, 3H), 3.37 (s, 3H), 2.06 (s, 3H), 1.57 (d, *J* = 6.7 Hz, 3H); ^{13}C NMR (100 MHz, $CDCl_3$): δ 169.8, 140.0, 132.4, 128.6, 128.2, 125.9, 121.1, 102.6, 91.9, 82.0, 60.7, 54.4 (2 x OMe), 21.3, 21.0; HRMS calcd for $C_{15}H_{18}O_4$: 262.1205, found: 262.1209

Spectral data for 1-(2-(dimethoxymethyl)phenyl)hept-1-yn-3-yl acetate (5g).

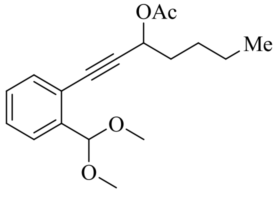

5g

Yellow oil, IR (neat, cm^{-1}): 3073 (m), 1734 (s), 1636 (w), 1448 (m), 1053 (s), 912 (s), 686 (s); ^{1}H NMR (400 MHz, $CDCl_3$): δ 7.51 (dd, *J* = 7.4 Hz, 1.2 Hz, 1H), 7.38 (dd, *J* = 7.4 Hz, 1.2 Hz, 1H), 7.26 (td, *J* = 7.4, 1.2 Hz, 1H), 7.19 (td, *J* = 7.4 Hz, 1.2 Hz, 1H), 5.56–5.53 (m, 2H), 3.33 (s, 3H), 3.32 (s, 3H), 2.04 (s, 3H), 1.84–1.79 (m, 2H), 1.50–1.42 (m, 2H), 1.39–1.29 (m, 2H), 0.88 (t, *J* = 7.3 Hz, 3H); ^{13}C NMR (100 MHz, $CDCl_3$): δ 169.7, 139.9, 132.3, 128.4, 128.1, 125.9, 121.1, 102.4, 91.1, 82.5, 64.4, 54.1 (2 x OMe), 34.3, 27.0, 22.1, 20.8, 13.7; HRMS calcd for $C_{18}H_{24}O_4$: 304.1675, found: 304.1671.

Spectral data for 2-(propan-2-ylidene)-2,3,4,5,6,7-hexahydro-1*H*-inden-1-one (4a).

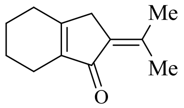

4a

Pale yellow oil, IR (neat, cm^{-1}): 2967 (s), 1672 (s), 1652 (m); ^{1}H NMR (400 MHz, $CDCl_3$): δ 2.91 (s, 2H), 2.28–2.25 (m, 5H), 2.14–2.10 (m, 2H), 1.81 (s, 3H), 1.70–1.65 (m, 2H), 1.64–1.60 (m, 2H); ^{13}C NMR (100 MHz, $CDCl_3$): δ 196.4, 162.7, 143.7, 141.7, 129.1, 35.8, 27.5, 23.8, 22.2, 21.7, 20.1, 19.4; HRMS calcd for $C_{12}H_{16}O$: 176.1201, found: 176.1191.

Spectral data for 2-(propan-2-ylidene)-2,3,5,6-tetrahydropentalen-1(4*H*)-one (4b).

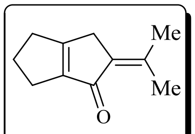

4b

Yellow solid, IR (neat, cm^{-1}): 2959 (s), 1671 (s), 1642 (m); ^{1}H NMR (400 MHz, $CDCl_3$): δ 2.98 (s, 2H), 2.55–2.51 (m, 2H), 2.43–2.38 (m, 2H), 2.29–2.21 (m, 5H), 1.82 (s, 3H); ^{13}C NMR (100 MHz, $CDCl_3$): δ 192.6, 175.1, 152.0, 144.1, 134.5, 31.6, 31.3, 27.0, 25.3, 23.7, 19.4; HRMS calcd for $C_{11}H_{14}O$: 162.1045, found: 162.1041.

Spectral data for (*E*)-2-(1-phenylethylidene)-2,3,5,6-tetrahydropentalen-1(4*H*)-one (4c).

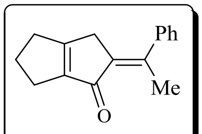

4c

Pale yellow oil, IR (neat, cm^{-1}): 2972 (s), 1682 (s), 1651 (m), 891 (s), 698 (s); ^{1}H NMR (400 MHz, $CDCl_3$): δ 7.37–7.33 (m, 2H), 7.29–7.26 (m, 1H), 7.22–7.20 (m, 2H), 2.89 (s, 2H), 2.58 (s, 3H), 2.50–2.44 (m, 4H), 2.29–2.22 (m, 2H); ^{13}C NMR (100 MHz, $CDCl_3$): δ 193.2, 177.4, 151.8, 146.3, 143.7, 135.8, 128.3, 127.4, 126.9, 32.4, 31.6, 27.0, 25.3, 19.6; HRMS calcd for $C_{16}H_{16}O$: 224.1201, found: 224.1200.

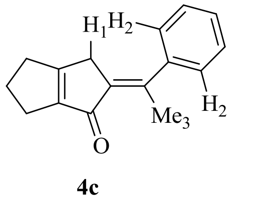

4c

Irradiation	Intensity increase
H_1 (δ 2.89)	H_2 (δ 7.22–7.20, 3.00 %).
H_2 (δ 7.22–7.20)	H_1 (δ 2.89, 1.75%), H_3 (δ 2.58, 1.51%).
H_3 (δ 2.58)	H_2 (δ 7.22–7.20, 0.76%).

Spectral data for (*E*)-2-(1-phenylpropylidene)-2,3,5,6-tetrahydropentalen-1(4*H*)-one (4d).

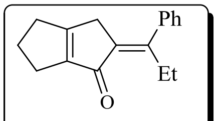

4d

Pale yellow oil, IR (neat, cm^{-1}): 2963 (s), 1674 (s), 1656 (m), 887 (s), 695 (s); ^1H NMR (400 MHz, $CDCl_3$): δ 7.37–7.32 (m, 2H), 7.30–7.26 (m, 1H), 7.17–7.14 (m, 2H), 3.12 (q, J = 7.5 Hz, 2H), 2.81 (s, 2H), 2.48–2.42 (m, 4H), 2.28–2.21 (m, 2H), 0.98 (t, J = 7.5 Hz, 3H); ^{13}C NMR (100 MHz, $CDCl_3$): δ 192.8, 177.3, 152.8, 151.9, 142.2, 135.2, 128.2, 127.3, 127.2, 32.3, 31.6, 27.0, 25.4, 25.3, 12.8; HRMS calcd for $C_{17}H_{18}O$: 238.1358, found: 238.1364.

Spectral data for 2-methyl-3-phenyl-5-(propan-2-ylidene)cyclopent-2-enone (4e).

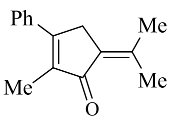

4e

Pale yellow oil, IR (neat, cm^{-1}): 1680 (s), 1645 (m), 897 (s), 687 (s); ^1H NMR (600 MHz, $CDCl_3$): δ 7.54–7.53 (m, 2H), 7.45–7.43 (m, 2H), 7.39–7.36 (m, 1H), 3.40 (s, 2H), 2.36 (s, 3H), 2.01 (t, J = 1.4 Hz, 3H), 1.94 (s, 3H); ^{13}C NMR (150 MHz, $CDCl_3$): δ 196.9, 156.6, 145.6, 139.7, 136.3, 129.1, 128.6 (1 x CH, 1 x C), 127.8, 35.2, 24.1, 20.0, 10.3; HRMS calcd for $C_{15}H_{16}O$: 212.1201, found: 212.1197.

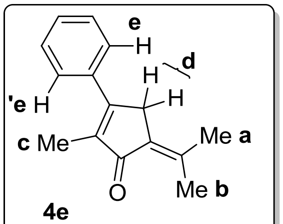

4e

Irradiation	Intensity increase
H**d** (δ 3.40)	H**a** (δ 1.94, 3.38%), H**e** (δ 7.52–7.54, 3.86%)
H**a** (δ 1.94)	H**d** (δ 3.40, 2.38%)
H**c** (δ 2.00)	H**e'** (δ 7.52–7.54, 1.26%).
H**e,e'** (δ 7.52–7.54)	H**d** (δ 3.40, 2.52%)

Spectral data for (*E*)-2-benzylidene-2,3,4,5,6,7-hexahydro-1*H*-inden-1-one (4f).

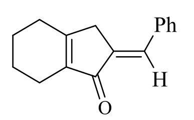

4f

Pale yellow oil, IR (neat, cm^{-1}): 2958 (s), 1669 (s), 1645 (m), 893 (s), 691 (s); ^{1}H NMR (600 MHz, $CDCl_3$): δ 7.56–7.55 (m, 2H), 7.40–7.37 (m, 2H), 7.34–7.31 (m, 2H), 3.36 (s, 2H), 2.42–2.40 (m, 2H), 2.27–2.24 (m, 2H), 1.79–1.75 (m, 2H), 1.71–1.68 (m, 2H); ^{13}C NMR (150 MHz, $CDCl_3$): δ 196.5, 166.7, 140.2, 135.7, 133.7, 130.2, 129.8, 129.0, 128.8, 36.0, 28.0, 22.3, 21.7, 20.4; HRMS calcd for $C_{16}H_{16}O$: 224.1201, found: 224.1195.

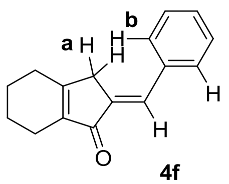

4f

Irradiation	Intensity increase
H**a** (δ 3.36)	H**b** (δ 7.55, 4.83%)
H**b** (δ 7.55)	H**d** (δ 3.36, 2.94%)

Spectral data for (*E*)-2-(1-phenylethylidene)-2,3,4,5,6,7-hexahydro-1*H*-inden-1-one (4g).

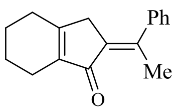

4g

Yellow oil, IR (neat, cm^{-1}): 2963 (s), 1672 (s), 1651 (m), 886 (s), 698 (s); ^{1}H NMR (400 MHz, $CDCl_3$): δ 7.37–7.34 (m, 2H), 7.30–7.26 (m, 1H), 7.24–7.21 (m, 2H), 2.87 (s, 2H), 2.59 (s, 3H), 2.24–2.17 (m, 4H), 1.70–1.63 (m, 4H); ^{13}C NMR (100 MHz, $CDCl_3$): δ 197.2, 165.0, 145.8, 143.9, 141.7, 130.6, 128.3, 127.4, 127.0, 36.8, 27.5, 22.2, 21.7, 20.2, 19.6; HRMS calcd for $C_{17}H_{18}O$: 238.1358, found: 238.1361.

Spectral data for (*E*)-2-(1-phenylpropylidene)-2,3,4,5,6,7-hexahydro-1*H*-inden-1-one (4h).

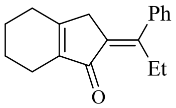

4h

Yellow oil, IR (neat, cm^{-1}): 2961 (s), 1676 (s), 1662 (m), 876 (s), 693 (s); ^{1}H NMR (400 MHz, $CDCl_3$): δ 7.37–7.33 (m, 2H), 7.30–7.26 (m, 1H), 7.18–7.16 (m, 2H), 3.14 (q, *J* = 7.5 Hz, 2H), 2.79 (s, 2H), 2.21–2.17 (m, 4H), 1.70–1.60 (m, 4H), 0.98 (t, *J* = 7.5 Hz, 3H); ^{13}C NMR (100 MHz, $CDCl_3$): δ 196.7, 164.9, 152.4, 142.4, 141.8, 129.9, 128.2, 127.3, 127.2, 36.7, 27.5, 25.5, 22.2, 21.8, 20.2, 12.8; HRMS calcd for $C_{18}H_{20}O$: 252.1514, found: 252.1512.

Spectral data for 2-(propan-2-ylidene)-2,3,4,5,6,7-hexahydro-1*H*-inden-1-one (d_1-4a).

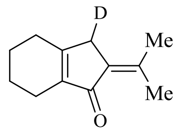

d_1-4a

Pale yellow oil, IR (neat, cm^{-1}): 2969 (s), 1674 (s), 1650 (m); ^{1}H NMR (400 MHz, $CDCl_3$): δ 2.92 (s, 1H), 2.31–2.28 (m, 5H), 2.16–2.12 (m, 2H), 1.83 (s, 3H), 1.73–1.60 (m, 4H); ^{13}C NMR (100 MHz, $CDCl_3$): δ 196.5, 162.8, 143.8, 141.9, 129.2, 35.5 (t, J = 19.7 Hz), 27.6, 23.9, 22.3, 21.8, 20.2, 19.5; HRMS calcd for $C_{12}H_{15}DO$: 177.1264, found: 177.1271.

Spectral data for 6,6-dimethyl-2-(propan-2-ylidene)-2,3,4,5,6,7-hexahydro-1*H*-inden-1-one (6a).

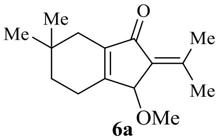

6a

Pale yellow oil, IR (neat, cm^{-1}): 2959 (s), 1663 (s), 1652 (m); ^{1}H NMR (400 MHz, $CDCl_3$): δ 4.93 (s, 1H), 2.94 (s, 3H), 2.43–2.36 (m, 1H), 2.30 (s, 3H), 2.27–2.20 (m, 1H), 1.99–1.96 (m, 5H), 1.46 (t, J = 7.6 Hz, 2H), 0.91 (s, 3H), 0.89 (s, 3H); ^{13}C NMR (100 MHz, $CDCl_3$): δ 194.8, 160.4, 149.3, 144.6, 128.4, 77.9, 50.2, 35.0, 33.7, 29.2, 28.0, 27.9, 23.0, 22.4, 20.1; HRMS calcd for $C_{15}H_{22}O_2$: 234.1620, found: 234.1623.

Spectral data for 2-cyclopentylidene-3-methoxy-2,3,5,6-tetrahydropentalen-1(4*H*)-one (6b).

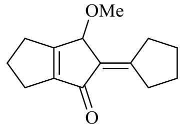

6b

Dark yellow oil, IR (neat, cm^{-1}): 2975 (s), 1673 (s), 1648 (m), 1121 (s); ^{1}H NMR (400 MHz, $CDCl_3$): δ 4.81 (s, 1H), 3.13 (s, 3H), 2.88–2.84 (m, 2H), 2.65–2.33 (m, 6H), 2.32–2.25 (m, 2H), 1.74–1.59 (m, 4H); ^{13}C NMR (100 MHz, $CDCl_3$): δ 190.2, 174.2, 159.5, 155.0, 130.6, 75.5, 52.8, 32.7, 32.0, 30.0, 26.9, 26.3, 25.3, 25.2; HRMS calcd for $C_{14}H_{18}O_2$: 218.1307, found: 218.1301.

Spectral data for (*E*)-2-benzylidene-3-methoxy-2,3,4,5,6,7-hexahydro-1*H*-inden-1-one (6c).

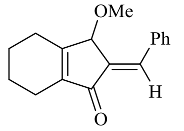

6c

Yellow oil, IR (neat, cm^{-1}): 2973 (s), 1672 (s), 1641 (m), 1124 (s), 885 (s), 691 (s); ^{1}H NMR (400 MHz, $CDCl_3$): δ 7.82–7.80 (m, 2H), 7.47 (d, *J* = 1.3 Hz, 1H), 7.41–7.32 (m, 3H), 5.31 (s, 1H), 2.92 (s, 3H), 2.53–2.47 (m, 1H), 2.34–2.29 (m, 3H), 1.85–1.69 (m, 4H); ^{13}C NMR (100 MHz, $CDCl_3$): δ 194.1, 164.9, 144.1, 134.1, 133.8, 131.5, 131.3, 129.6, 128.6, 76.6, 49.7, 24.7, 21.8, 21.5, 20.1; HRMS calcd for $C_{17}H_{18}O_2$: 254.1307, found: 254.1312.

Spectral data for 3-methoxy-2-(propan-2-ylidene)-2,3,4,5,6,7-hexahydro-1*H*-inden-1-one (6d).

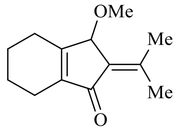

6d

Brown oil, IR (neat, cm^{-1}): 2965 (s), 1669 (s), 1636 (m), 1120 (s); ^{1}H NMR (400 MHz, $CDCl_3$): δ 4.90 (s, 1H), 2.95 (s, 3H), 2.42–2.35 (m, 1H), 2.30 (s, 3H), 2.24–2.13 (m, 3H), 1.95 (s, 3H), 1.79–1.66 (m, 4H); ^{13}C NMR (100 MHz, $CDCl_3$): δ 194.6, 161.5, 149.2, 145.1, 128.2, 78.2, 50.2, 24.5, 23.1, 22.0, 21.6, 20.1, 19.9; HRMS calcd for $C_{13}H_{18}O_2$: 206.1307, found: 206.1313.

Spectral data for (*E*)-2-benzylidene-3-methoxy-2,3-dihydro-1*H*-inden-1-one (6e).

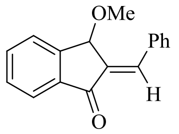

6e

Yellow oil, IR (neat, cm^{-1}): 2982 (m), 1672 (s), 1642 (m), 891 (s), 692 (s); ^{1}H NMR (400 MHz, $CDCl_3$): δ 7.95–7.92 (m, 2H), 7.89 (d, *J* = 7.7 Hz, 1H), 7.82 (d, *J* = 1.5 Hz, 1H), 7.74–7.66 (m, 2H), 7.53–7.49 (m, 1H), 7.47–7.40 (m, 3H), 5.95 (d, *J* = 1.5 Hz, 1H), 2.91 (s, 3H); ^{13}C NMR (100 MHz, $CDCl_3$): δ 192.4, 148.5, 138.8, 138.6, 135.1, 134.0, 133.4, 131.9, 130.4, 129.8, 128.8, 126.2, 123.7, 74.7, 50.4; HRMS calcd for $C_{17}H_{14}O_2$: 250.0994, found: 250.0991.

Spectral data for (*E*)-2-ethylidene-3-methoxy-2,3-dihydro-1*H*-inden-1-one (6f).

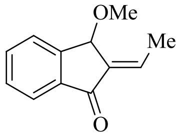

6f

Yellow oil, IR (neat, cm^{-1}): 2953 (m), 1668 (s), 1642 (m), 887 (s), 697 (s); ^{1}H NMR (400 MHz, $CDCl_3$): δ 7.85 (d, *J* = 7.8 Hz, 1H), 7.71–7.65 (m, 2H), 7.52–7.48 (m, 1H), 7.19–7.13 (m, 1H), 5.73 (s, 1H), 3.00 (s, 3H), 2.10 (d, *J* = 7.4 Hz, 3H); ^{13}C NMR (100 MHz, $CDCl_3$): δ 191.2, 148.5, 138.9, 138.3, 136.7, 134.9, 129.6, 126.2, 123.8, 74.8, 51.2, 14.8; HRMS calcd for $C_{12}H_{12}O_2$: 188.0837, found: 188.0841.

Spectral data for (*E*)-3-methoxy-2-pentylidene-2,3-dihydro-1*H*-inden-1-one (6g).

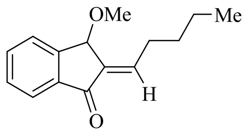

6g

Yellow oil, IR (neat, cm^{-1}): 2957 (m), 1670 (s), 1645 (m), 895 (s), 691 (s); ^{1}H NMR (400 MHz, $CDCl_3$): δ 7.84 (d, *J* = 7.6 Hz, 1H), 7.69–7.63 (m, 2H), 7.50–7.46 (m, 1H), 7.07 (td, *J* = 7.8 Hz, 1.7 Hz, 1H), 5.70 (s, 1H), 2.98 (s, 3H), 2.51–2.45 (m, 2H), 1.56–1.49 (m, 2H), 1.44–1.35 (m, 2H), 0.92 (t, *J* = 7.2 Hz, 3H); ^{13}C NMR (100 MHz, $CDCl_3$): δ 191.4, 148.5, 143.6, 139.0, 135.6, 134.9, 129.7, 126.2, 123.8, 74.9, 51.2, 30.5, 28.8, 22.6, 13.8; HRMS calcd for $C_{15}H_{18}O_2$: 230.1307, found: 230.1310.

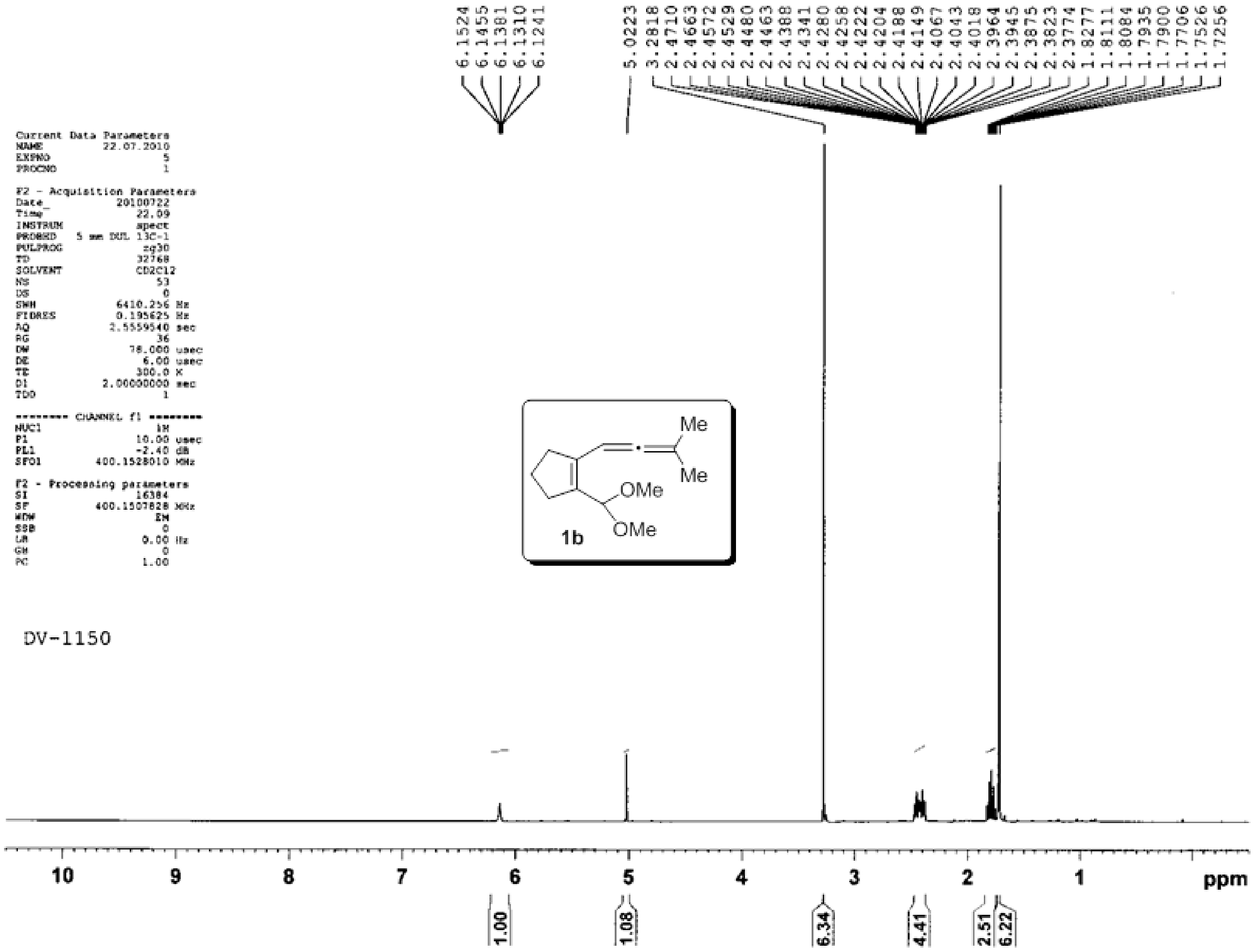
6.1524
6.1455
6.1381
6.1310
6.1241
5.0223
3.2818
2.4710
2.4663
2.4572
2.4529
2.4480
2.4463
2.4388
2.4341
2.4280
2.4258
2.4222
2.4204
2.4188
2.4149
2.4067
2.4043
2.4018
2.3964
2.3945
2.3875
2.3823
2.3774
1.8277
1.8111
1.8084
1.7935
1.7900
1.7706
1.7526
1.7256
Current Data Parameters
NAME 22.07.2010
EXPNO 5
PROCNO 1
F2 - Acquisition Parameters
Date_ 20100722
Time 22.09
INSTRUM spect
PROBHD 5 mm DUL 13C-1
PULPROG zg30
TD 32768
SOLVENT CD2Cl2
NS 53
DS 0
SWH 6410.256 Hz
FIDRES 0.195625 Hz
AQ 2.5559540 sec
RG 36
DW 78.000 usec
DE 6.00 usec
TE 300.0 K
D1 2.00000000 sec
TD0 1
======== CHANNEL f1 ========
NUC1 1H
P1 10.00 usec
PL1 -2.40 dB
SFO1 400.1528010 MHz
F2 - Processing parameters
SI 16384
SF 400.1507828 MHz
WDW EM
SSB 0
LB 0.00 Hz
GB 0
PC 1.00
DV-1150
Me
Me
OMe
OMe
1b
10
9
8
7
6
5
4
3
2
1
ppm
1.00
1.08
6.34
4.41
2.51
6.22

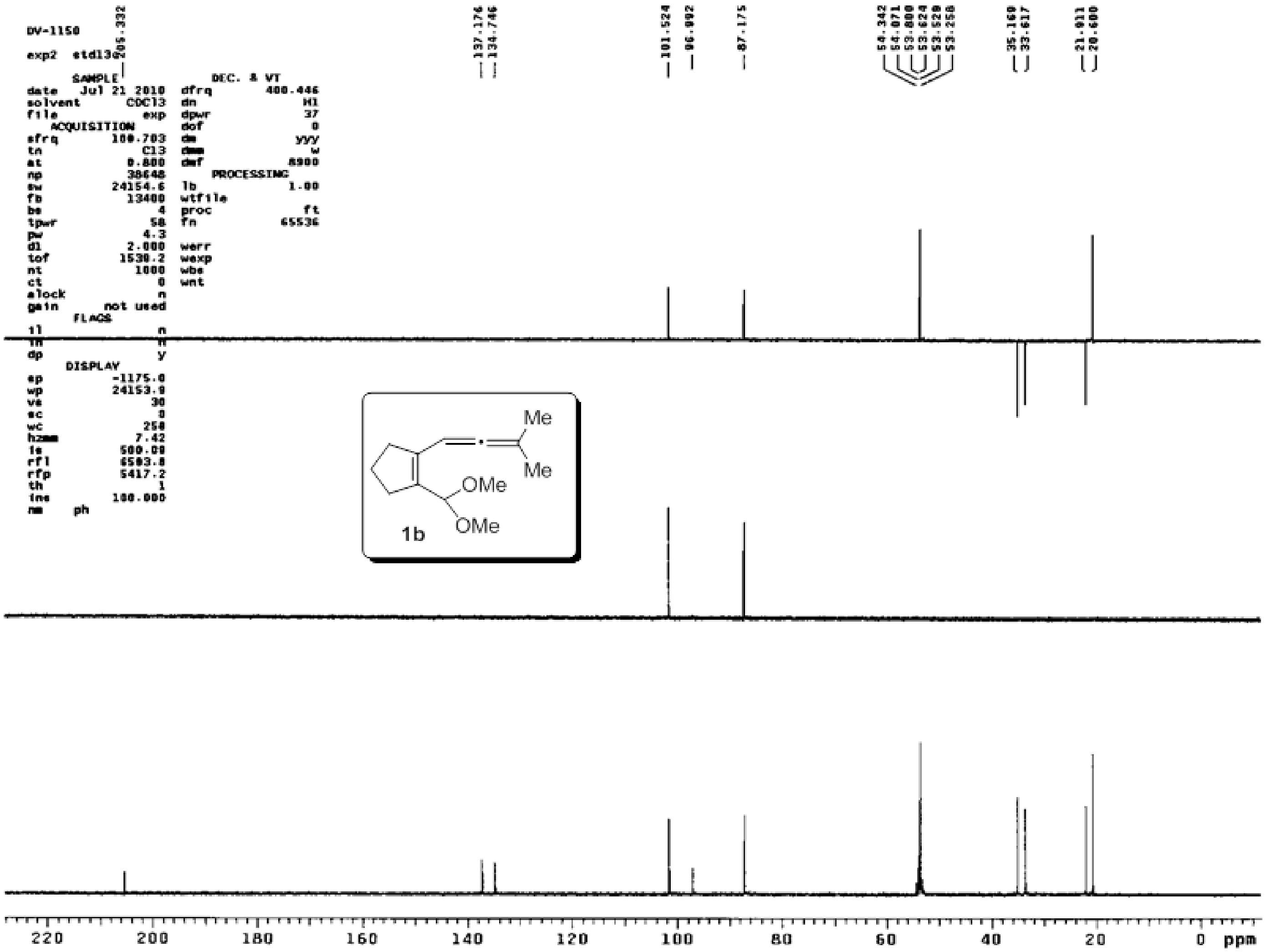
DV-1150
exp2 std13C
SAMPLE
date Jul 21 2010
solvent CDCl3
file exp
ACQUISITION
DEC. & VT
PROCESSING
FLAGS
DISPLAY
205.332
137.176
134.746
101.524
96.892
87.175
54.342
54.071
53.800
53.624
53.520
53.258
35.160
33.617
21.011
20.600
Me
Me
OMe
OMe
1b
ppm

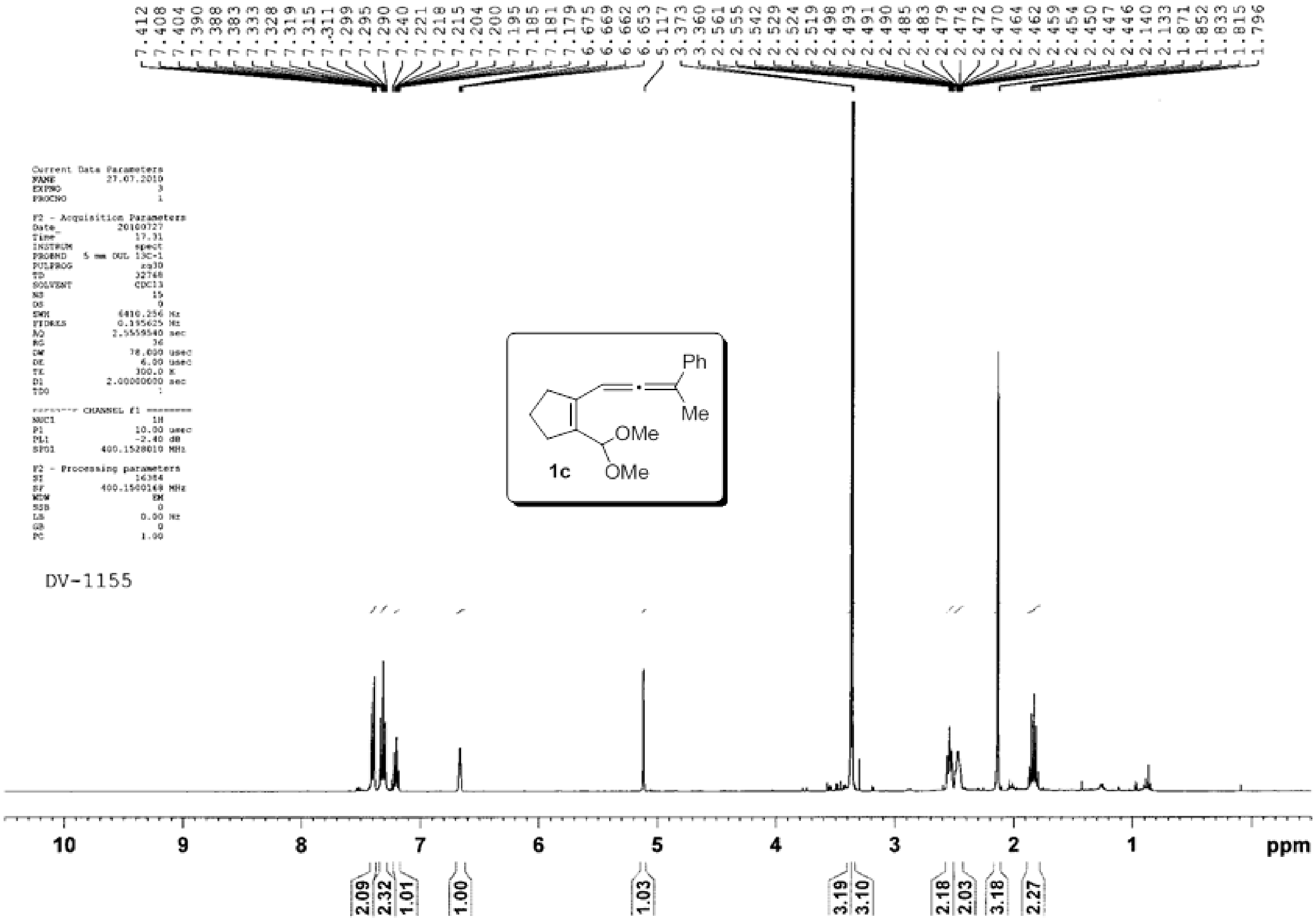
7.412 7.408 7.404 7.390 7.388 7.383 7.333 7.328 7.319 7.315 7.311 7.299 7.295 7.290 7.240 7.221 7.218 7.215 7.204 7.200 7.195 7.185 7.181 7.179 6.675 6.669 6.662 6.653 5.117 3.373 3.360 2.561 2.555 2.542 2.529 2.524 2.519 2.498 2.493 2.491 2.490 2.485 2.483 2.479 2.474 2.472 2.470 2.464 2.462 2.459 2.454 2.450 2.447 2.446 2.140 2.133 1.871 1.852 1.833 1.815 1.796
DV-1155
Ph
Me
OMe
1c
OMe
10 9 8 7 6 5 4 3 2 1 ppm
2.09 2.32 1.01 1.00 1.03 3.19 3.10 2.18 2.03 3.18 2.27

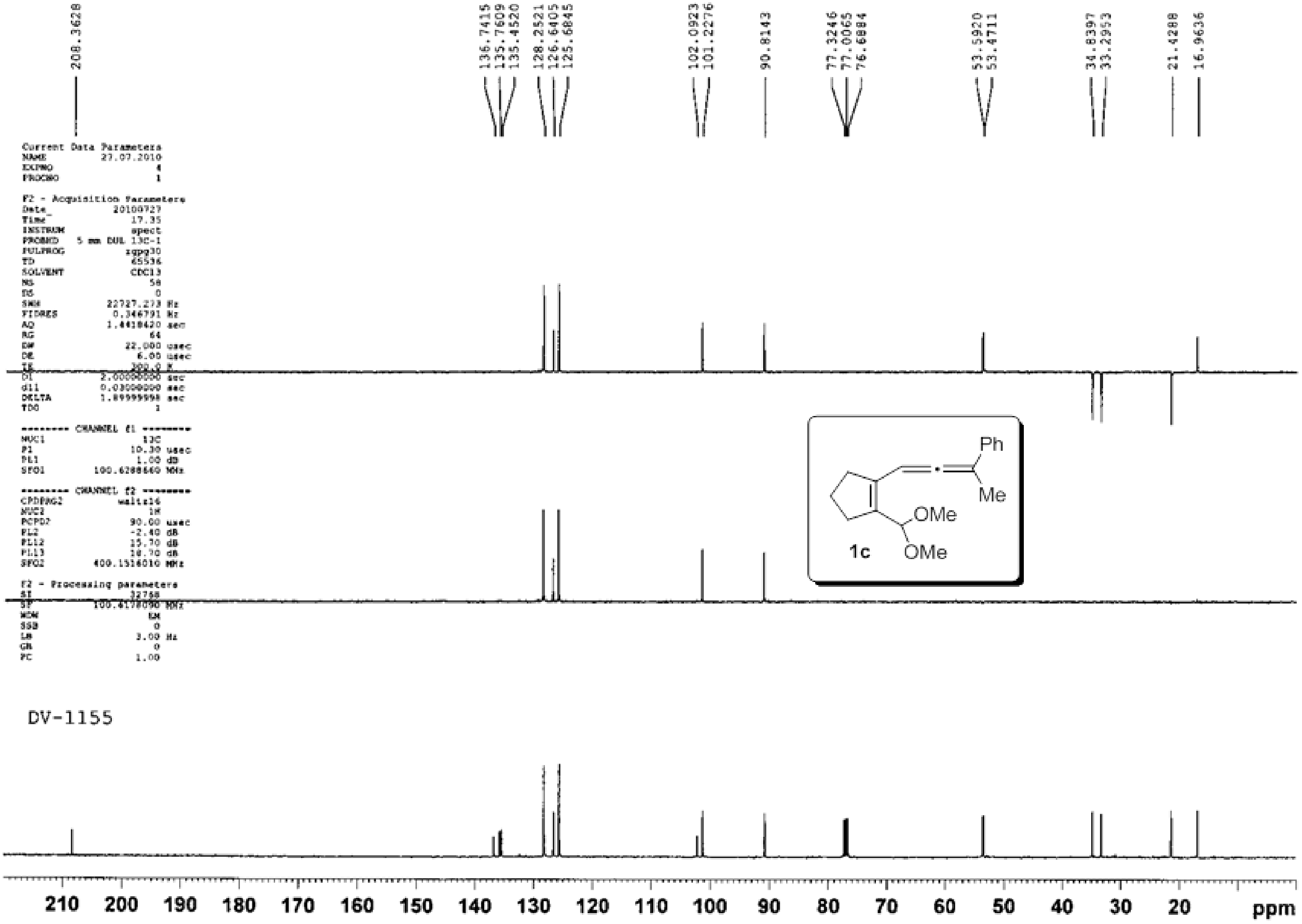

208.3628
136.7415
135.7609
135.4520
128.2521
126.6405
125.6845
102.0923
101.2276
90.8143
77.3246
77.0065
76.6884
53.5920
53.4711
34.8397
33.2953
21.4288
16.9636
Ph
Me
OMe
OMe
1c
DV-1155
210 200 190 180 170 160 150 140 130 120 110 100 90 80 70 60 50 40 30 20 ppm

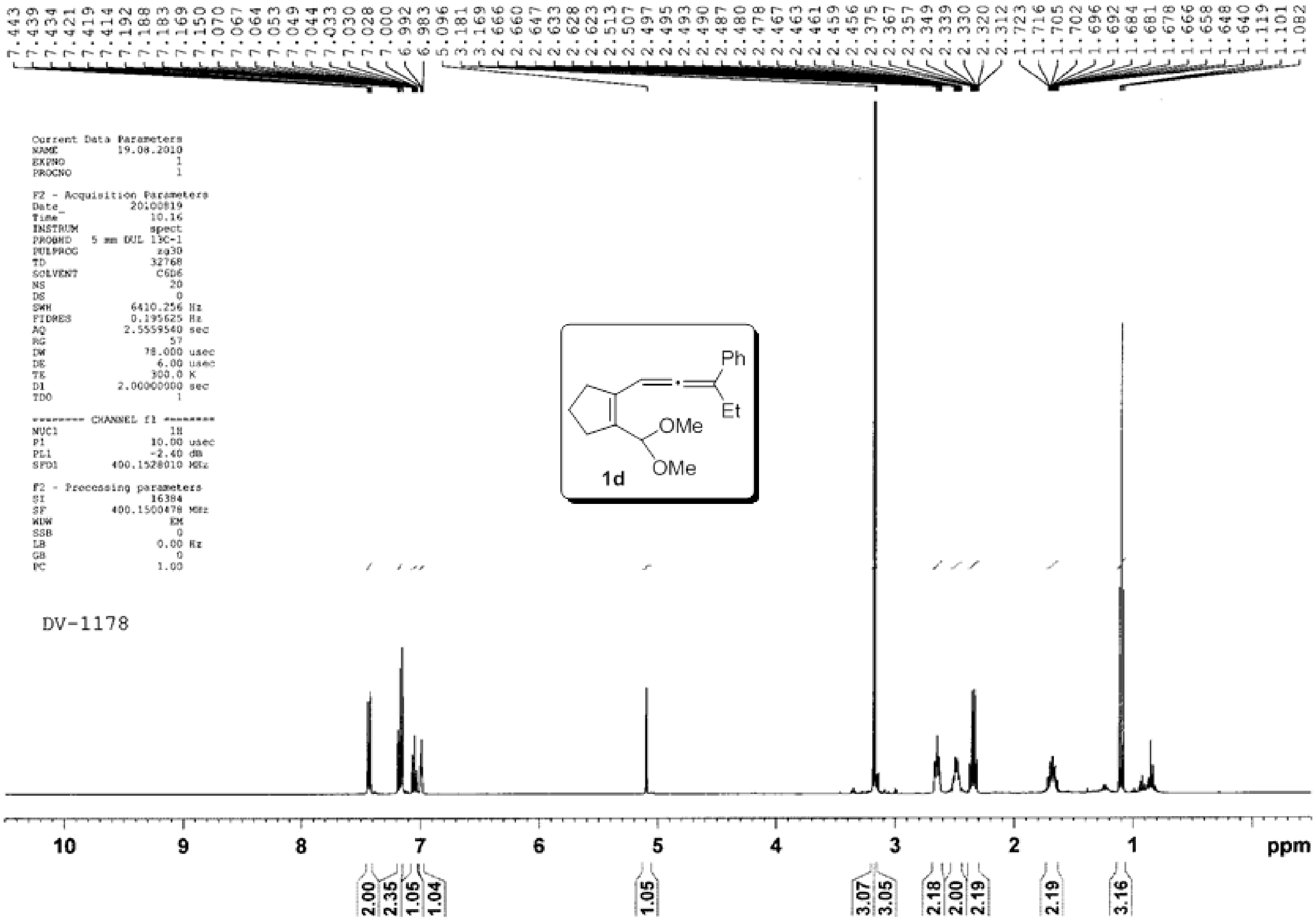
Current Data Parameters
NAME 19.08.2010
EXPNO 1
PROCNO 1
F2 - Acquisition Parameters
Date_ 20100819
Time 10.16
INSTRUM spect
PROBHD 5 mm DUL 13C-1
PULPROG zg30
TD 32768
SOLVENT C6D6
NS 20
DS 0
SWH 6410.256 Hz
FIDRES 0.195625 Hz
AQ 2.5559540 sec
RG 57
DW 78.000 usec
DE 6.00 usec
TE 300.0 K
D1 2.00000000 sec
TD0 1
======== CHANNEL f1 ========
NUC1 1H
P1 10.00 usec
PL1 -2.40 dB
SFO1 400.1528010 MHz
F2 - Processing parameters
SI 16384
SF 400.1500478 MHz
WDW EM
SSB 0
LB 0.00 Hz
GB 0
PC 1.00
DV-1178
Ph
Et
OMe
OMe
1d
ppm

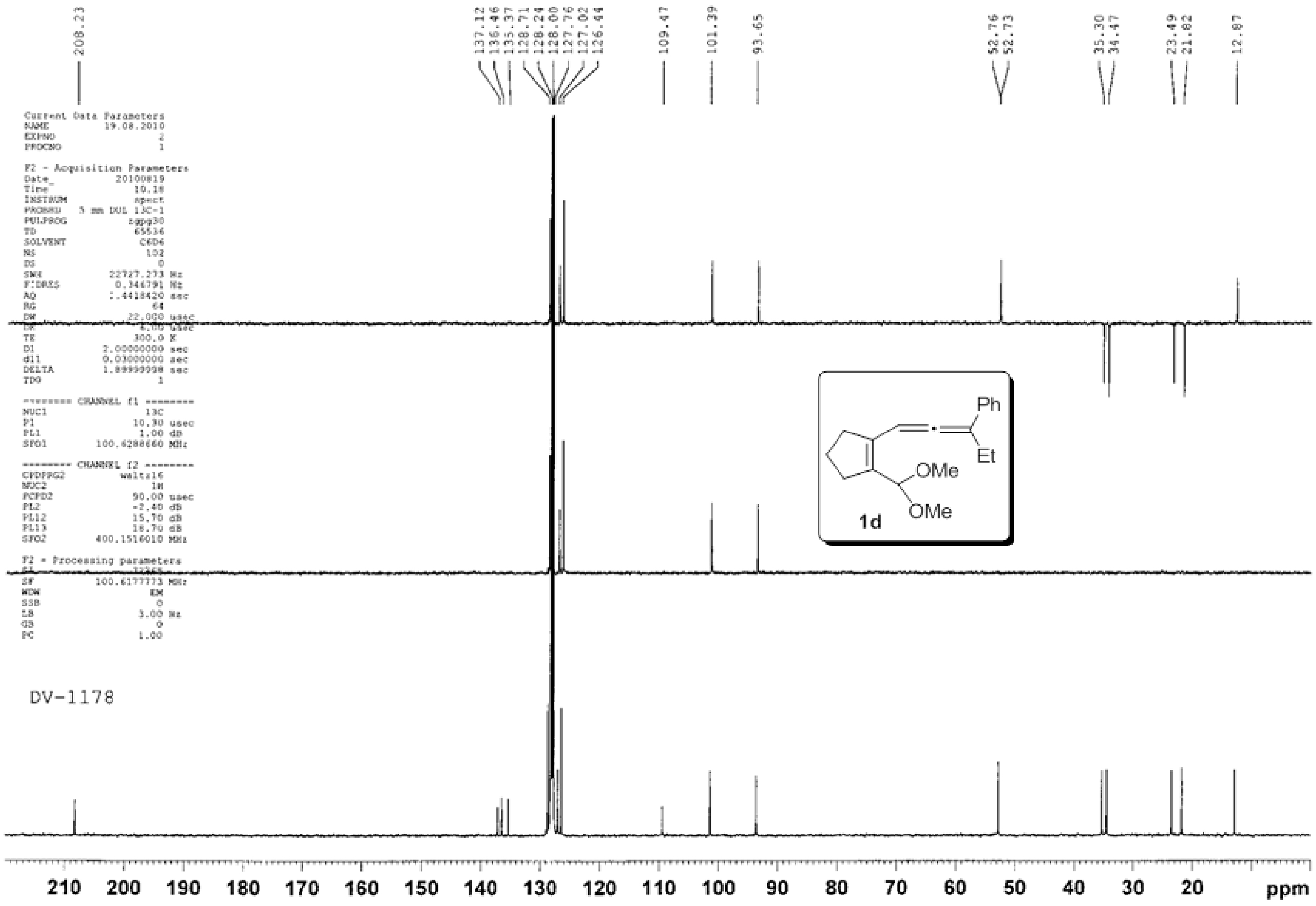
208.23
137.12
136.46
135.37
128.71
128.24
128.00
127.76
127.02
126.44
109.47
101.39
93.65
52.76
52.73
35.30
34.47
23.49
21.82
12.87
Current Data Parameters
NAME 19.08.2010
EXPNO 2
PROCNO 1
F2 - Acquisition Parameters
Date_ 20100819
SOLVENT C6D6
NS 102
SFO1 100.6288660 MHz
SFO2 400.1516010 MHz
SF 100.6177773 MHz
F2 - Processing parameters
DV-1178
Ph
Et
OMe
OMe
1d
210
200
190
180
170
160
150
140
130
120
110
100
90
80
70
60
50
40
30
20
ppm

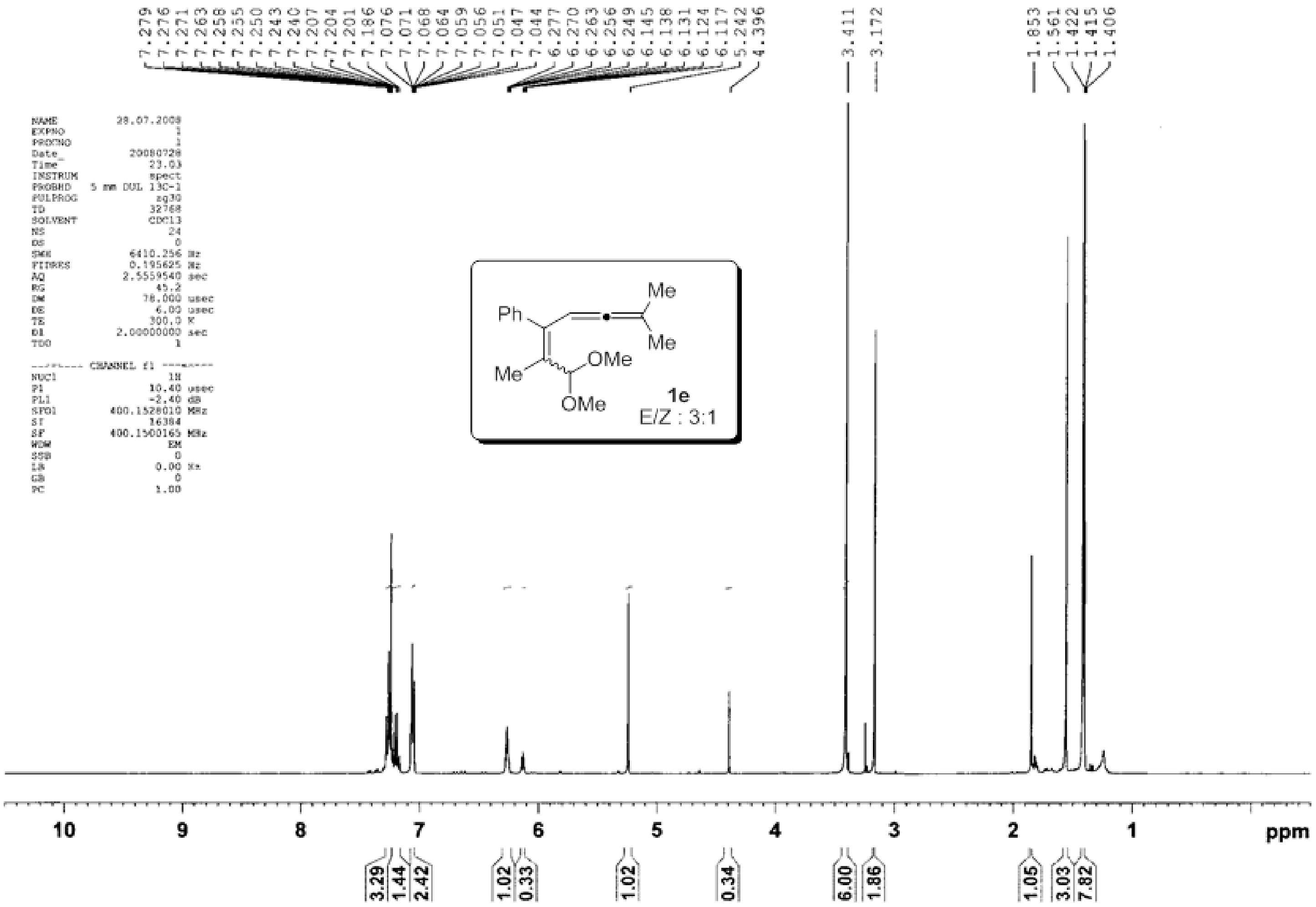
7.279 7.276 7.271 7.263 7.258 7.255 7.250 7.243 7.240 7.207 7.204 7.201 7.186 7.076 7.071 7.068 7.064 7.059 7.056 7.051 7.047 7.044 6.277 6.270 6.263 6.256 6.249 6.145 6.138 6.131 6.124 6.117 5.242 4.396
3.411 3.172
1.853 1.561 1.422 1.415 1.406
NAME 28.07.2008
EXPNO 1
PROCNO 1
Date_ 20080728
Time 23.03
INSTRUM spect
PROBHD 5 mm DUL 13C-1
PULPROG zg30
TD 32768
SOLVENT CDCl3
NS 24
DS 0
SWH 6410.256 Hz
FIDRES 0.195625 Hz
AQ 2.5559540 sec
RG 45.2
DW 78.000 usec
DE 6.00 usec
TE 300.0 K
D1 2.00000000 sec
TD0 1
CHANNEL f1
NUC1 1H
P1 10.40 usec
PL1 -2.40 dB
SFO1 400.1528010 MHz
SI 16384
SF 400.1500165 MHz
WDW EM
SSB 0
LB 0.00 Hz
GB 0
PC 1.00
Ph
Me
Me
Me
OMe
OMe
1e
E/Z : 3:1
10 9 8 7 6 5 4 3 2 1 ppm
3.29 1.44 2.42 1.02 0.33 1.02 0.34 6.00 1.86 1.05 3.03 7.82

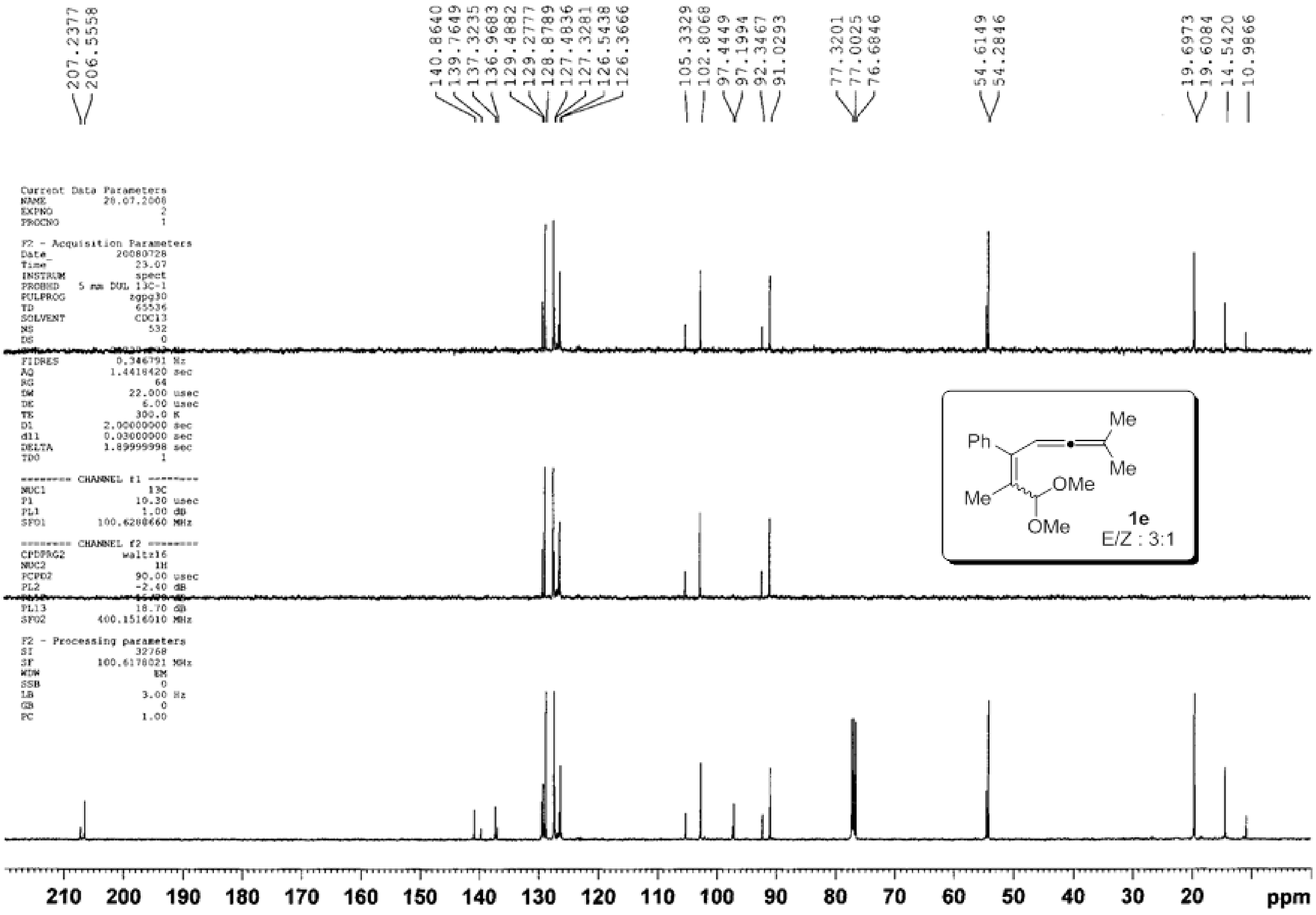
207.2377
206.5558
140.8640
139.7649
137.3235
136.9683
129.4882
129.2777
128.8789
127.4836
127.3281
126.5438
126.3666
105.3329
102.8068
97.4449
97.1994
92.3467
91.0293
77.3201
77.0025
76.6846
54.6149
54.2846
19.6973
19.6084
14.5420
10.9866
Current Data Parameters
NAME 28.07.2008
EXPNO 2
PROCNO 1
F2 - Acquisition Parameters
Date_ 20080728
Time 23.07
INSTRUM spect
PROBHD 5 mm DUL 13C-1
PULPROG zgpg30
TD 65536
SOLVENT CDCl3
NS 532
DS 0
FIDRES 0.346791 Hz
AQ 1.4418420 sec
RG 64
DW 22.000 usec
DE 6.00 usec
TE 300.0 K
D1 2.00000000 sec
d11 0.03000000 sec
DELTA 1.89999998 sec
TD0 1
======== CHANNEL f1 ========
NUC1 13C
P1 10.30 usec
PL1 1.00 dB
SFO1 100.6288660 MHz
======== CHANNEL f2 ========
CPDPRG2 waltz16
NUC2 1H
PCPD2 90.00 usec
PL2 -2.40 dB
PL13 18.70 dB
SFO2 400.1516010 MHz
F2 - Processing parameters
SI 32768
SF 100.6178021 MHz
WDW EM
SSB 0
LB 3.00 Hz
GB 0
PC 1.00
Ph
Me
Me
Me
OMe
OMe
1e
E/Z : 3:1
210 200 190 180 170 160 150 140 130 120 110 100 90 80 70 60 50 40 30 20 ppm

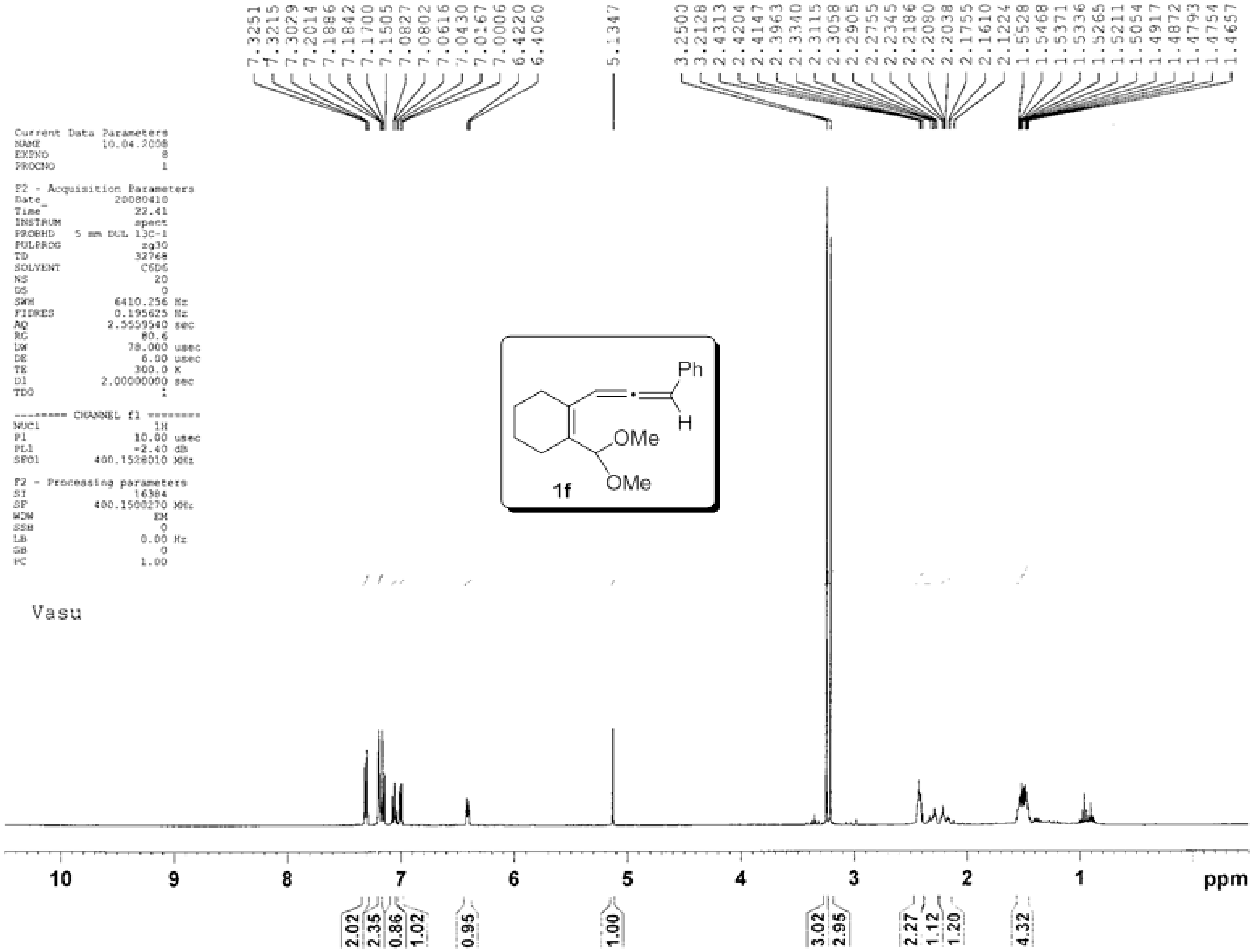

Current Data Parameters
NAME 10.04.2008
EXPNO 8
PROCNO 1
F2 - Acquisition Parameters
Date_ 20080410
Time 22.41
INSTRUM spect
PROBHD 5 mm DUL 13C-1
PULPROG zg30
TD 32768
SOLVENT C6D6
NS 20
DS 0
SWH 6410.256 Hz
FIDRES 0.195625 Hz
AQ 2.5559540 sec
RG 80.6
DW 78.000 usec
DE 6.00 usec
TE 300.0 K
D1 2.00000000 sec
TD0 1
======== CHANNEL f1 ========
NUC1 1H
P1 10.00 usec
PL1 -2.40 dB
SFO1 400.1528010 MHz
F2 - Processing parameters
SI 16384
SF 400.1500270 MHz
WDW EM
SSB 0
LB 0.00 Hz
GB 0
PC 1.00
Vasu
Ph
H
OMe
OMe
1f
ppm

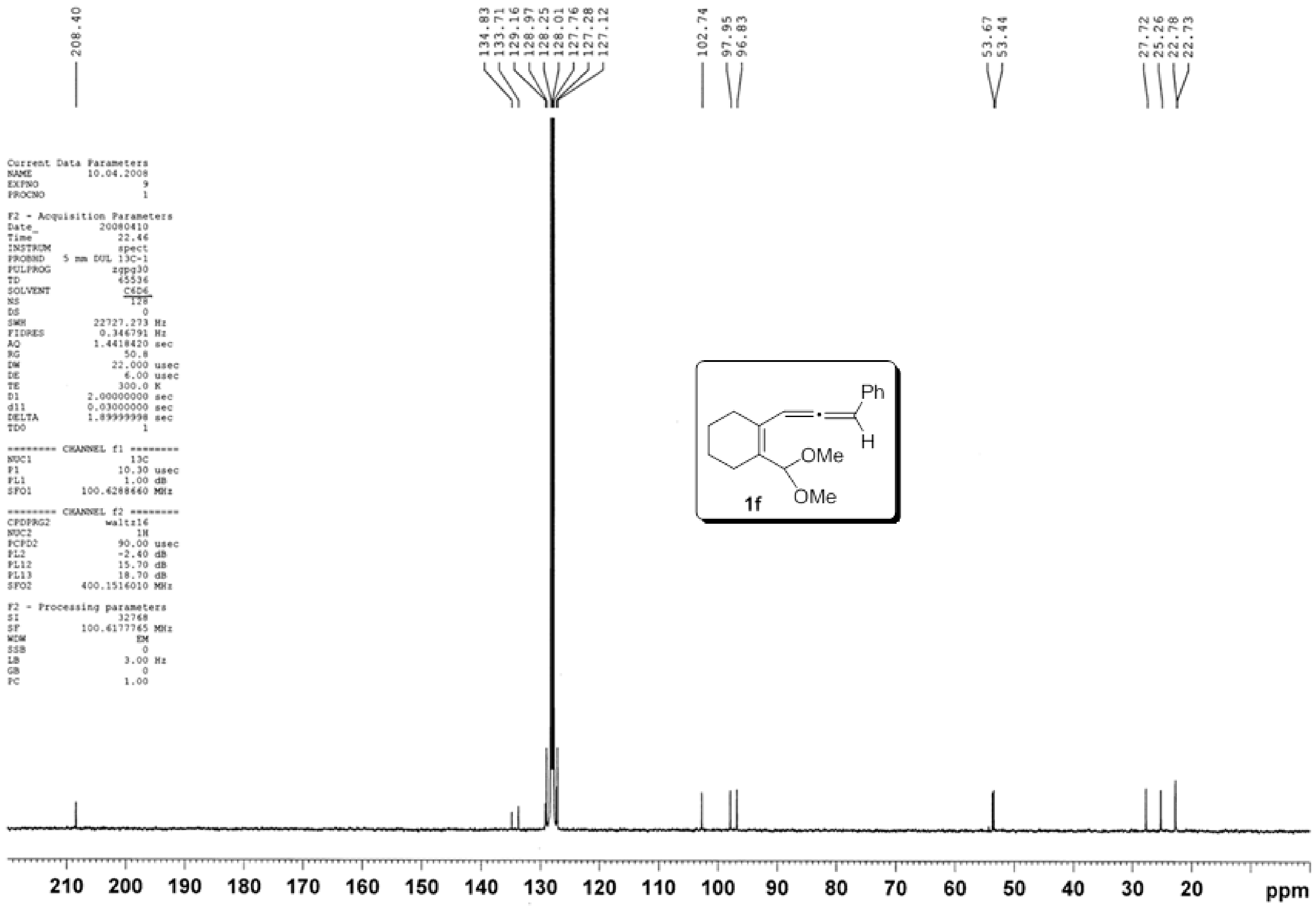

208.40
134.83
133.71
129.16
128.97
128.25
128.01
127.76
127.28
127.12
102.74
97.95
96.83
53.67
53.44
27.72
25.26
22.78
22.73
Current Data Parameters
NAME 10.04.2008
EXPNO 9
PROCNO 1
F2 - Acquisition Parameters
Date_ 20080410
Time 22.46
INSTRUM spect
PROBHD 5 mm DUL 13C-1
PULPROG zgpg30
TD 65536
SOLVENT C6D6
NS 128
DS 0
SWH 22727.273 Hz
FIDRES 0.346791 Hz
AQ 1.4418420 sec
RG 50.8
DW 22.000 usec
DE 6.00 usec
TE 300.0 K
D1 2.00000000 sec
d11 0.03000000 sec
DELTA 1.89999998 sec
TD0 1
======== CHANNEL f1 ========
NUC1 13C
P1 10.30 usec
PL1 1.00 dB
SFO1 100.6288660 MHz
======== CHANNEL f2 ========
CPDPRG2 waltz16
NUC2 1H
PCPD2 90.00 usec
PL2 -2.40 dB
PL12 15.70 dB
PL13 18.70 dB
SFO2 400.1516010 MHz
F2 - Processing parameters
SI 32768
SF 100.6177765 MHz
WDW EM
SSB 0
LB 3.00 Hz
GB 0
PC 1.00
Ph
H
OMe
OMe
1f
210 200 190 180 170 160 150 140 130 120 110 100 90 80 70 60 50 40 30 20 ppm

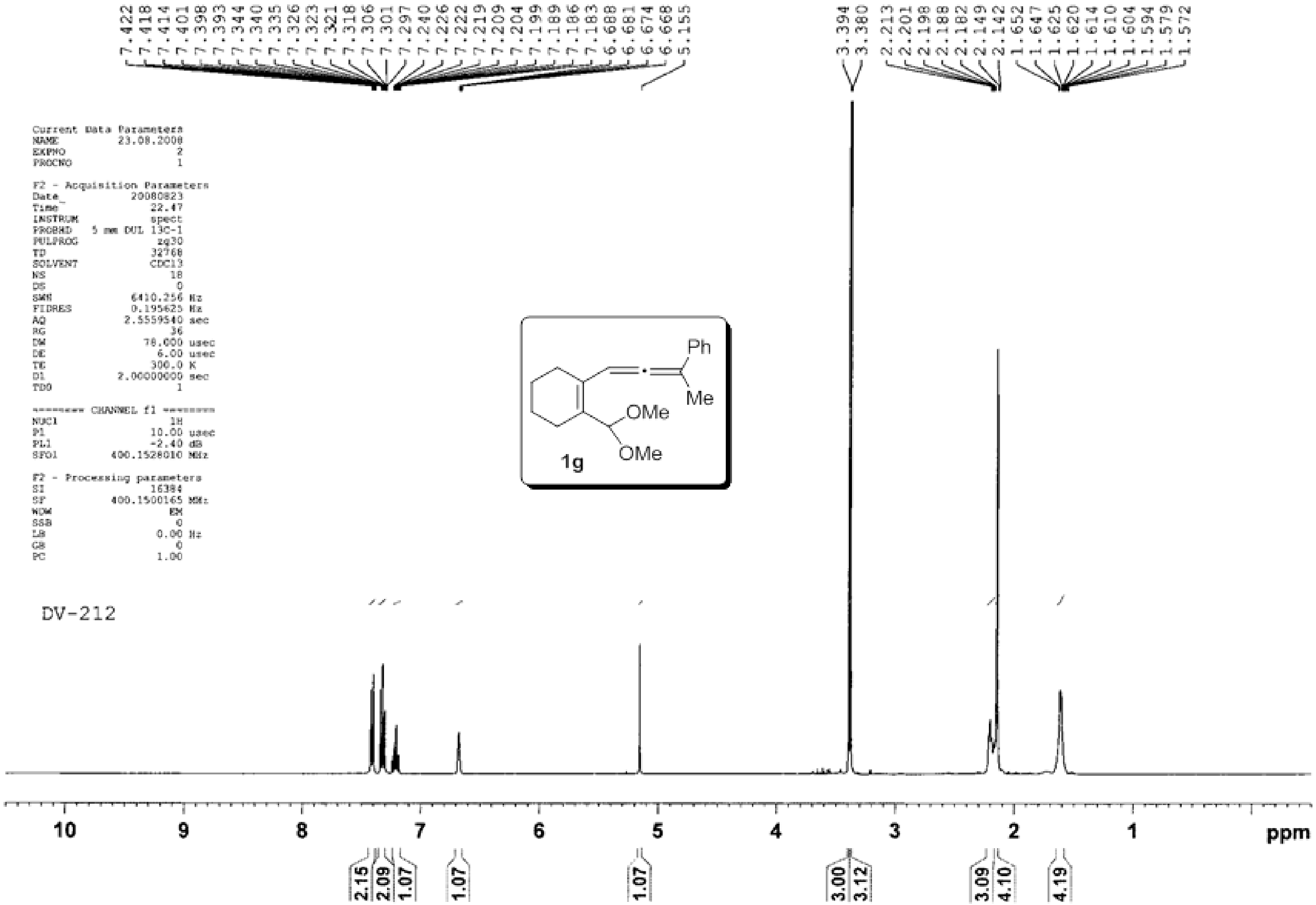

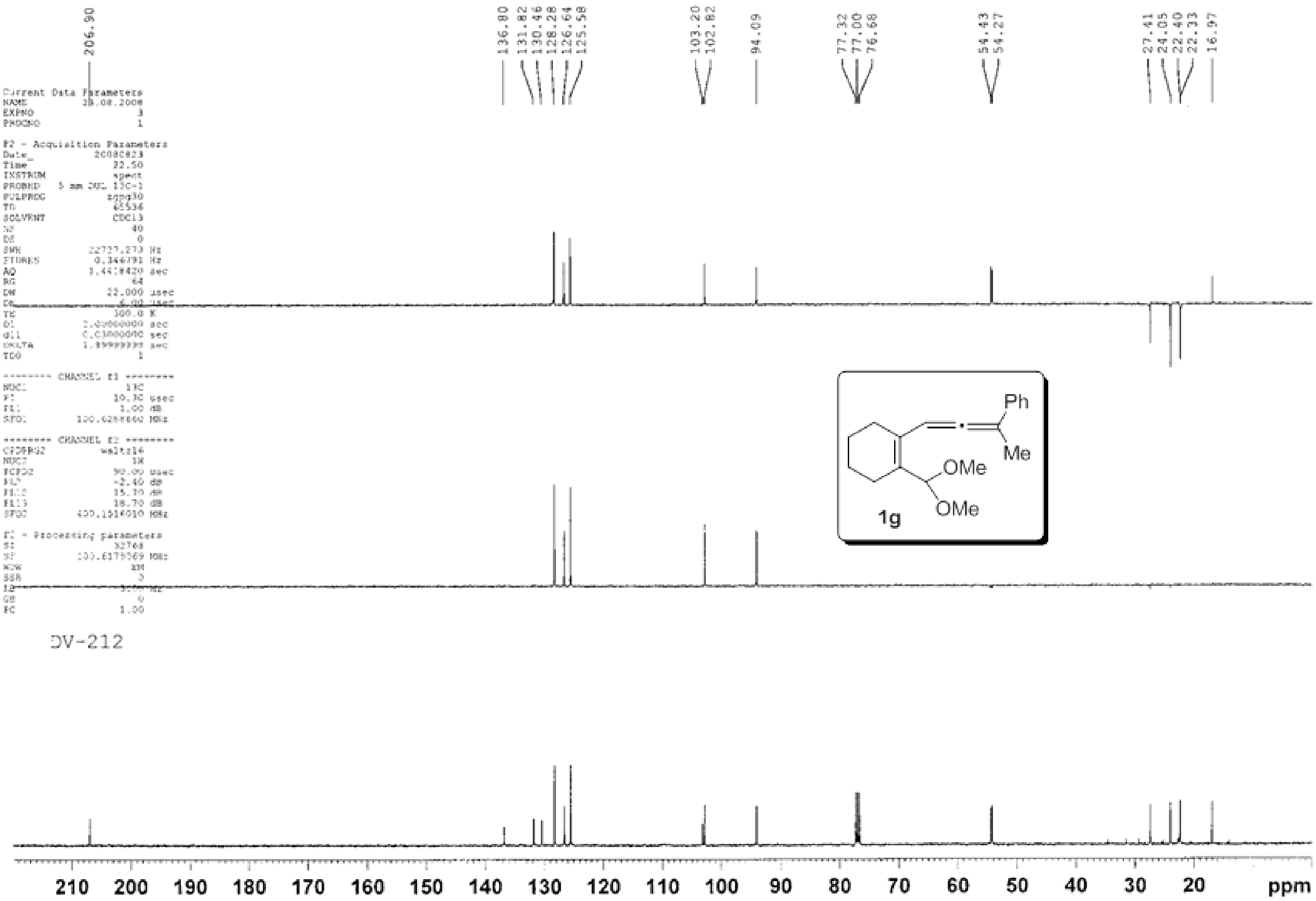
206.90
136.80
131.82
130.46
128.28
126.64
125.58
103.20
102.82
94.09
77.32
77.00
76.68
54.43
54.27
27.41
24.05
22.40
22.33
16.97
DV-212
Ph
Me
OMe
OMe
1g
210 200 190 180 170 160 150 140 130 120 110 100 90 80 70 60 50 40 30 20 ppm

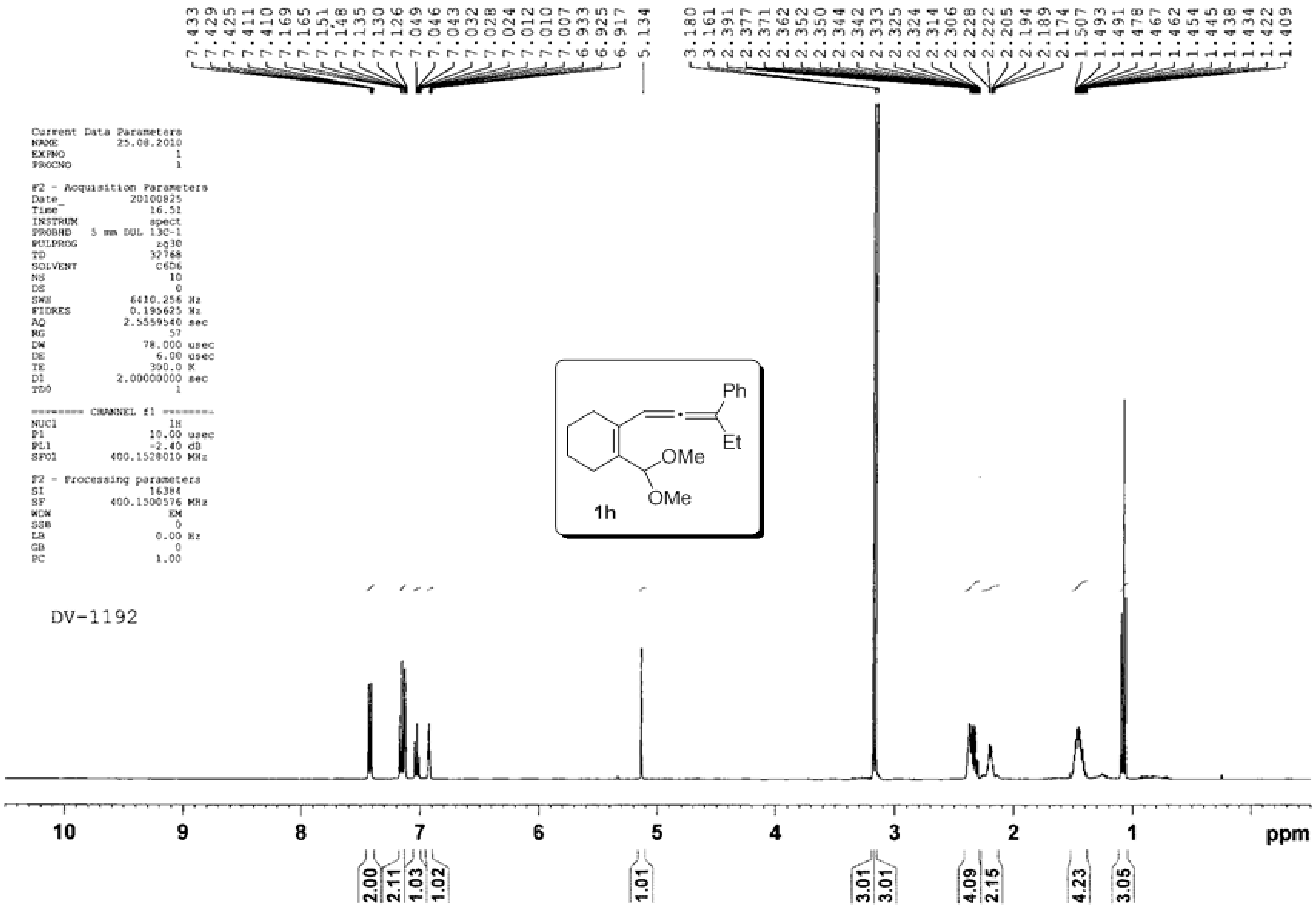
DV-1192
Ph
Et
OMe
OMe
1h
ppm

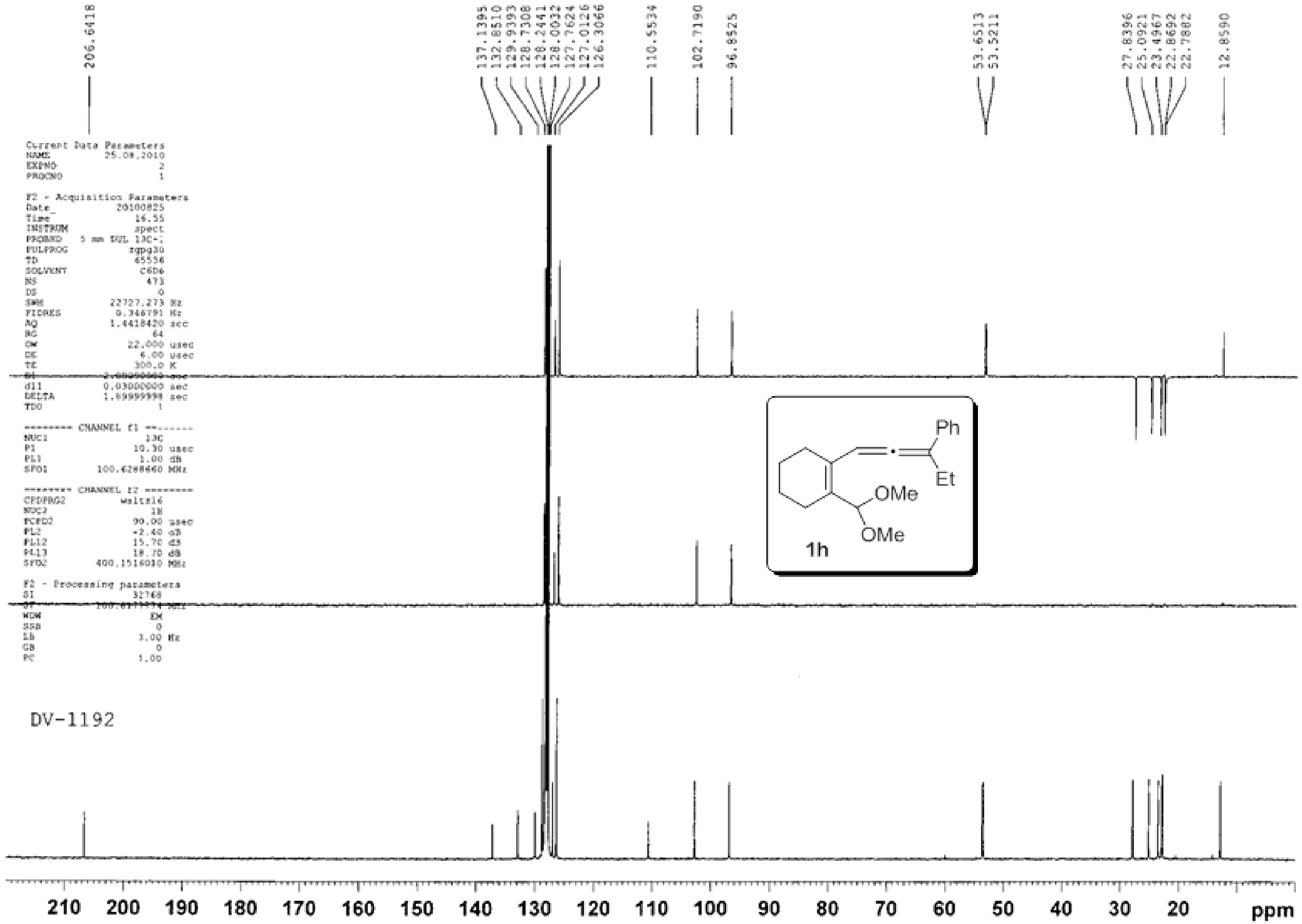
206.6418
137.1395
132.8510
129.9393
128.7308
128.2441
128.0032
127.7624
127.0126
126.3066
110.5534
102.7190
96.8525
53.6513
53.5211
27.8396
25.0921
23.4967
22.8692
22.7882
12.8590
DV-1192
Ph
Et
OMe
OMe
1h
210 200 190 180 170 160 150 140 130 120 110 100 90 80 70 60 50 40 30 20 ppm

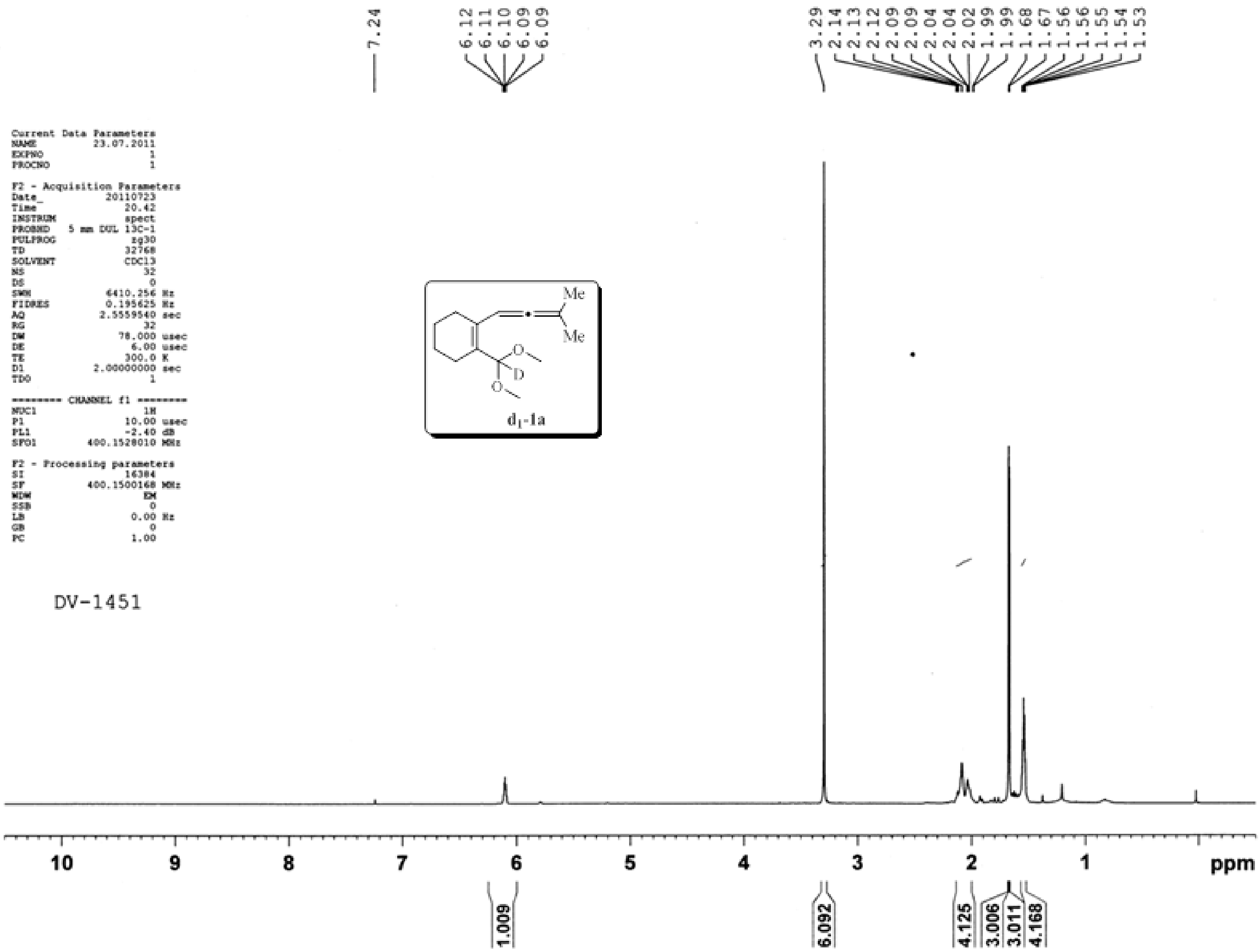
DV-1451
d1-1a
Me
Me
D
ppm

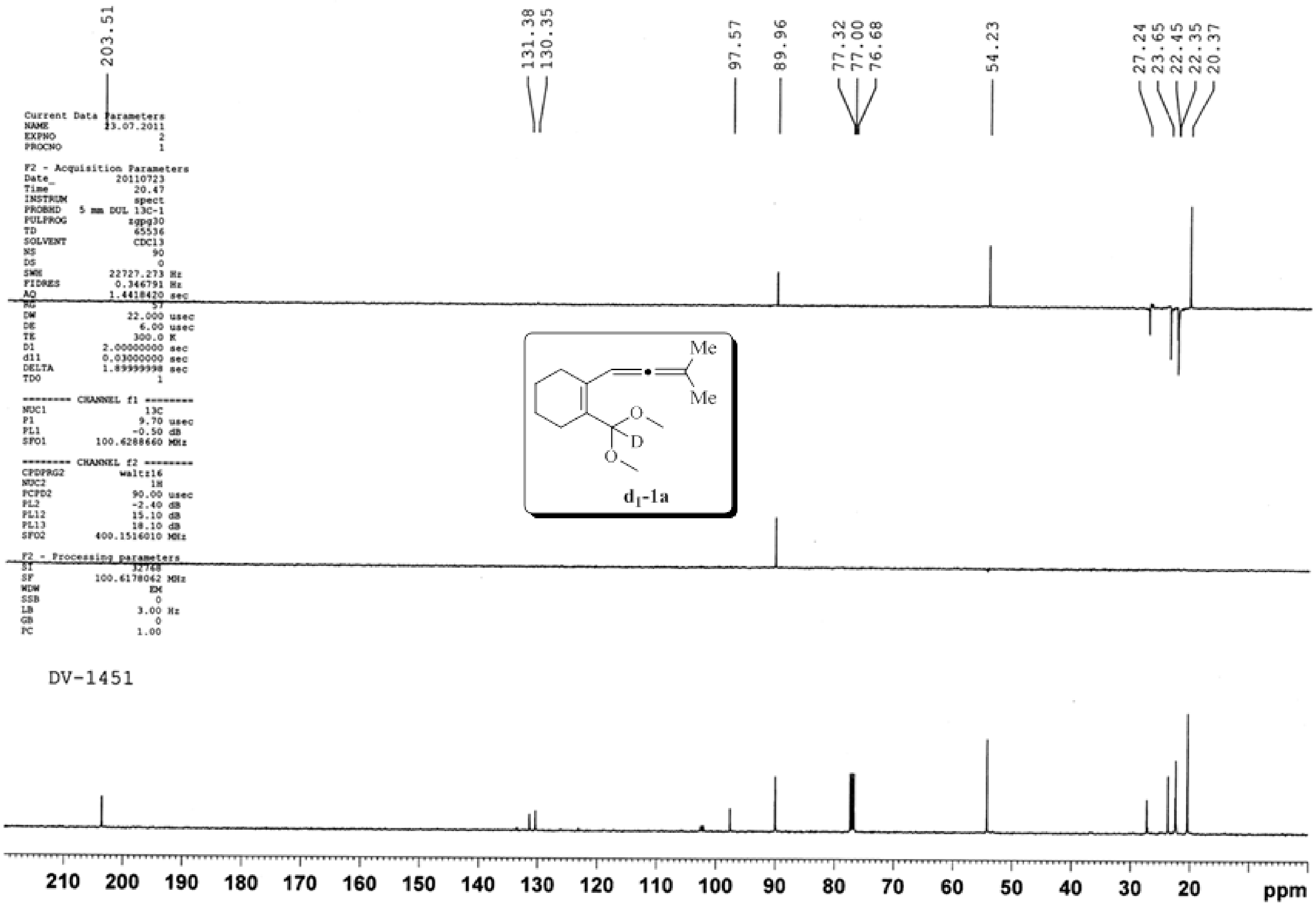

203.51
131.38
130.35
97.57
89.96
77.32
77.00
76.68
54.23
27.24
23.65
22.45
22.35
20.37
Current Data Parameters
NAME 23.07.2011
EXPNO 2
PROCNO 1
F2 - Acquisition Parameters
Date_ 20110723
Time 20.47
INSTRUM spect
PROBHD 5 mm DUL 13C-1
PULPROG zgpg30
TD 65536
SOLVENT CDCl3
NS 90
DS 0
SWH 22727.273 Hz
FIDRES 0.346791 Hz
AQ 1.4418420 sec
RG 57
DW 22.000 usec
DE 6.00 usec
TE 300.0 K
D1 2.00000000 sec
d11 0.03000000 sec
DELTA 1.89999998 sec
TD0 1
======== CHANNEL f1 ========
NUC1 13C
P1 9.70 usec
PL1 -0.50 dB
SFO1 100.6288660 MHz
======== CHANNEL f2 ========
CPDPRG2 waltz16
NUC2 1H
PCPD2 90.00 usec
PL2 -2.40 dB
PL12 15.10 dB
PL13 18.10 dB
SFO2 400.1516010 MHz
F2 - Processing parameters
SI 32768
SF 100.6178062 MHz
WDW EM
SSB 0
LB 3.00 Hz
GB 0
PC 1.00
Me
Me
O
D
O
d1-1a
DV-1451
210
200
190
180
170
160
150
140
130
120
110
100
90
80
70
60
50
40
30
20
ppm

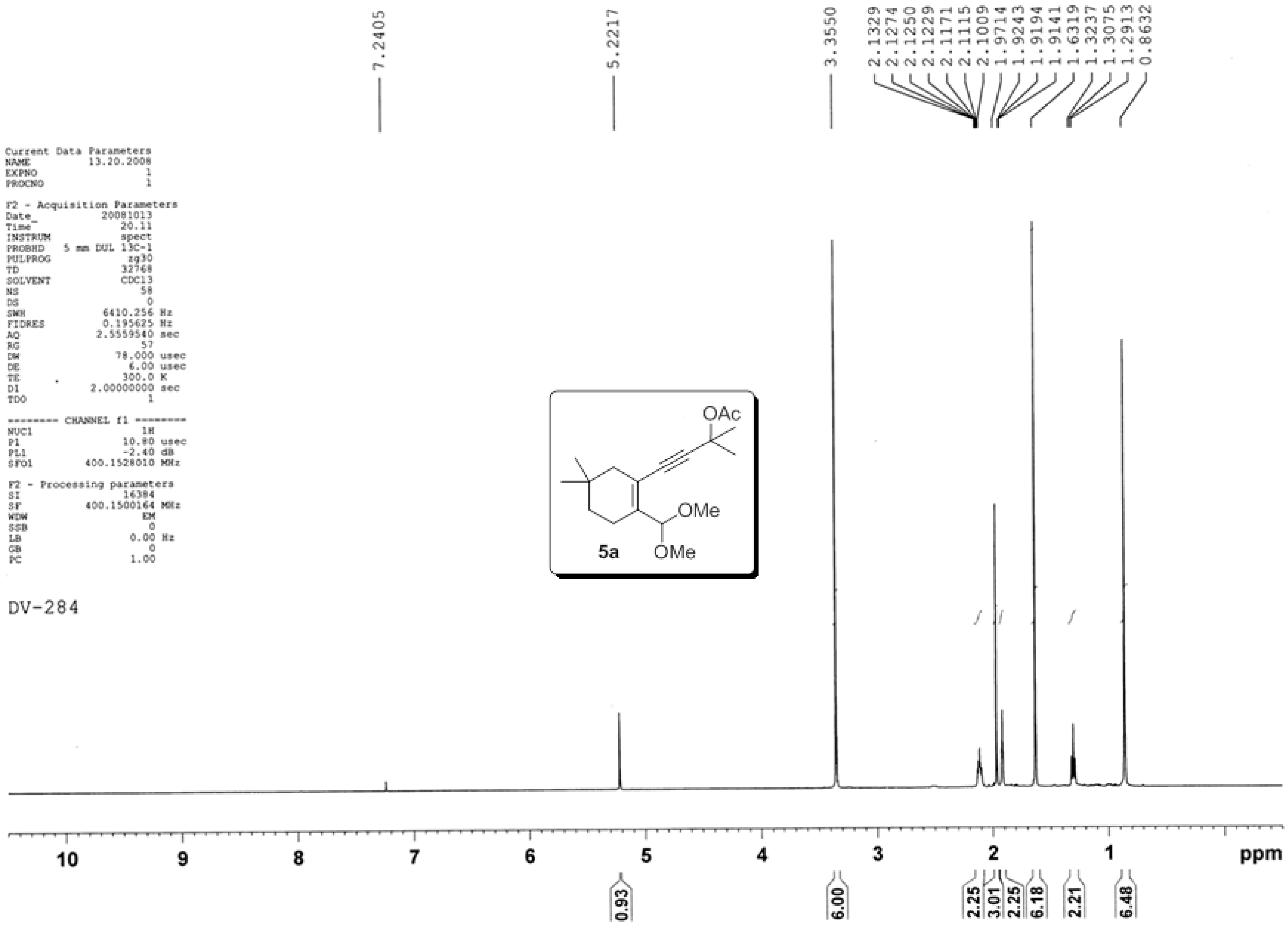
7.2405
5.2217
3.3550
2.1329
2.1274
2.1250
2.1229
2.1171
2.1115
2.1009
1.9714
1.9243
1.9194
1.9141
1.6319
1.3237
1.3075
1.2913
0.8632
Current Data Parameters
NAME 13.20.2008
EXPNO 1
PROCNO 1
F2 - Acquisition Parameters
Date_ 20081013
Time 20.11
INSTRUM spect
PROBHD 5 mm DUL 13C-1
PULPROG zg30
TD 32768
SOLVENT CDCl3
NS 58
DS 0
SWH 6410.256 Hz
FIDRES 0.195625 Hz
AQ 2.5559540 sec
RG 57
DW 78.000 usec
DE 6.00 usec
TE 300.0 K
D1 2.00000000 sec
TD0 1
======== CHANNEL f1 ========
NUC1 1H
P1 10.80 usec
PL1 -2.40 dB
SFO1 400.1528010 MHz
F2 - Processing parameters
SI 16384
SF 400.1500164 MHz
WDW EM
SSB 0
LB 0.00 Hz
GB 0
PC 1.00
DV-284
OAc
OMe
OMe
5a
10
9
8
7
6
5
4
3
2
1
ppm
0.93
6.00
2.25
3.01
2.25
6.18
2.21
6.48

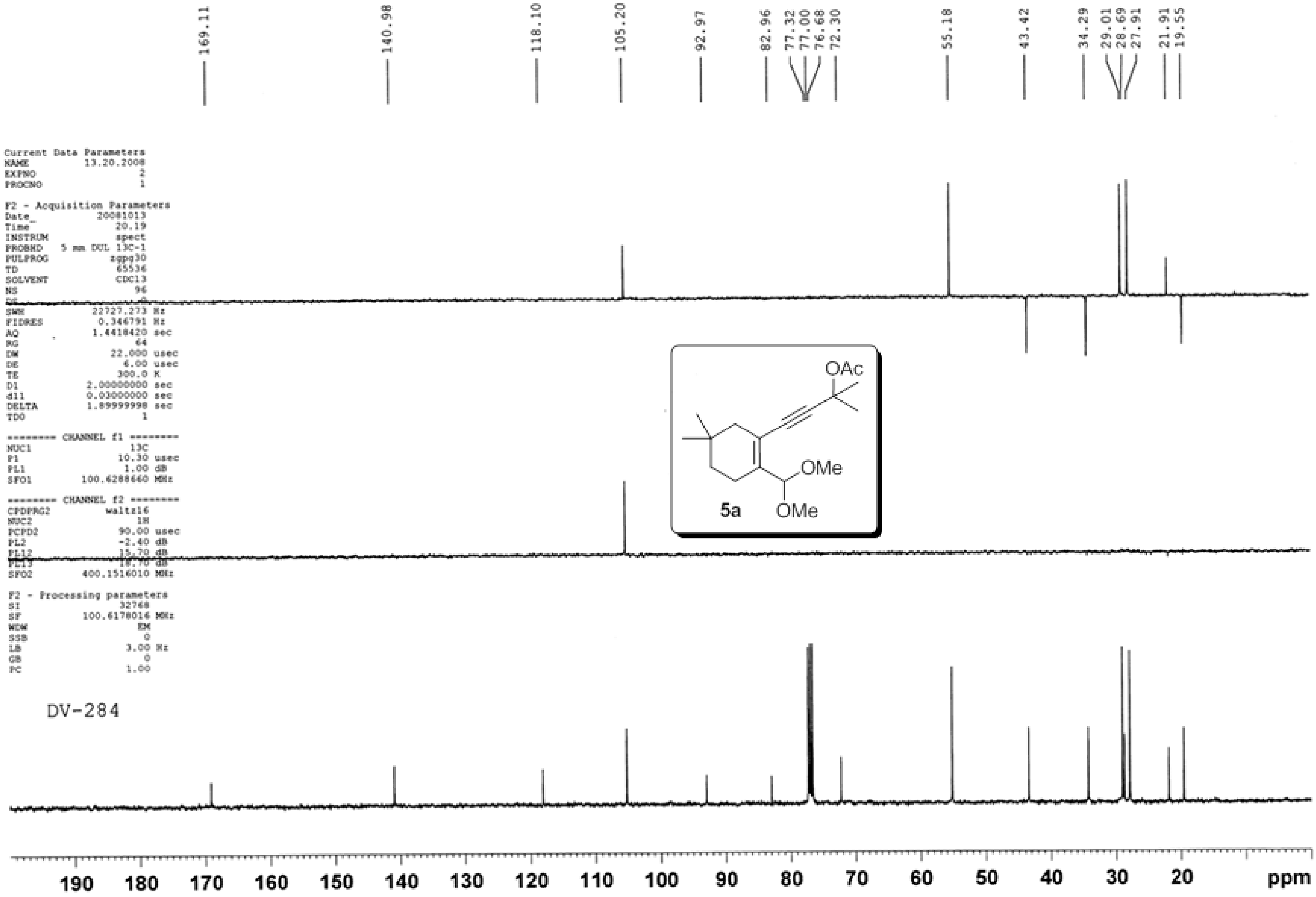
169.11
140.98
118.10
105.20
92.97
82.96
77.32
77.00
76.68
72.30
55.18
43.42
34.29
29.01
28.69
27.91
21.91
19.55
Current Data Parameters
NAME 13.20.2008
EXPNO 2
PROCNO 1
F2 - Acquisition Parameters
Date_ 20081013
Time 20.19
INSTRUM spect
PROBHD 5 mm DUL 13C-1
PULPROG zgpg30
TD 65536
SOLVENT CDCl3
NS 96
SWH 22727.273 Hz
FIDRES 0.346791 Hz
AQ 1.4418420 sec
RG 64
DW 22.000 usec
DE 6.00 usec
TE 300.0 K
D1 2.00000000 sec
d11 0.03000000 sec
DELTA 1.89999998 sec
TD0 1
======== CHANNEL f1 ========
NUC1 13C
P1 10.30 usec
PL1 1.00 dB
SFO1 100.6288660 MHz
======== CHANNEL f2 ========
CPDPRG2 waltz16
NUC2 1H
PCPD2 90.00 usec
PL2 -2.40 dB
PL12 15.70 dB
SFO2 400.1516010 MHz
F2 - Processing parameters
SI 32768
SF 100.6178016 MHz
WDW EM
SSB 0
LB 3.00 Hz
GB 0
PC 1.00
DV-284
OAc
OMe
OMe
5a
190
180
170
160
150
140
130
120
110
100
90
80
70
60
50
40
30
20
ppm

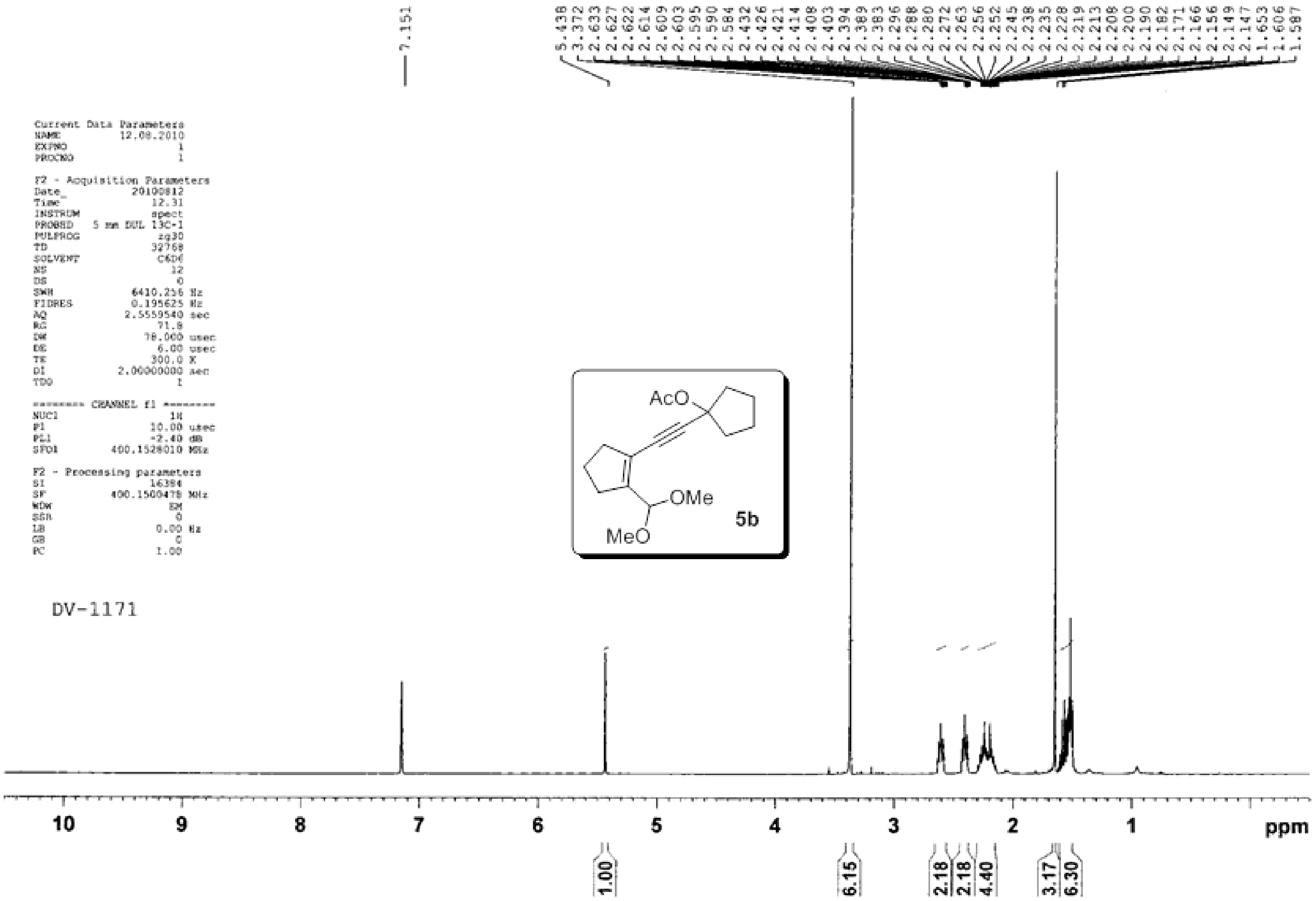

7.151
5.438
3.372
Current Data Parameters
NAME 12.08.2010
EXPNO 1
PROCNO 1
F2 - Acquisition Parameters
Date_ 20100812
Time 12.31
INSTRUM spect
PROBHD 5 mm DUL 13C-1
PULPROG zg30
TD 32768
SOLVENT C6D6
NS 12
DS 0
SWH 6410.256 Hz
FIDRES 0.195625 Hz
AQ 2.5559540 sec
RG 71.8
DW 78.000 usec
DE 6.00 usec
TE 300.0 K
D1 2.00000000 sec
TD0 1
======== CHANNEL f1 ========
NUC1 1H
P1 10.00 usec
PL1 -2.40 dB
SFO1 400.1528010 MHz
F2 - Processing parameters
SI 16384
SF 400.1500478 MHz
WDW EM
SSB 0
LB 0.00 Hz
GB 0
PC 1.00
DV-1171
AcO
OMe
MeO
5b
ppm
1.00
6.15
2.18
2.18
4.40
3.17
6.30

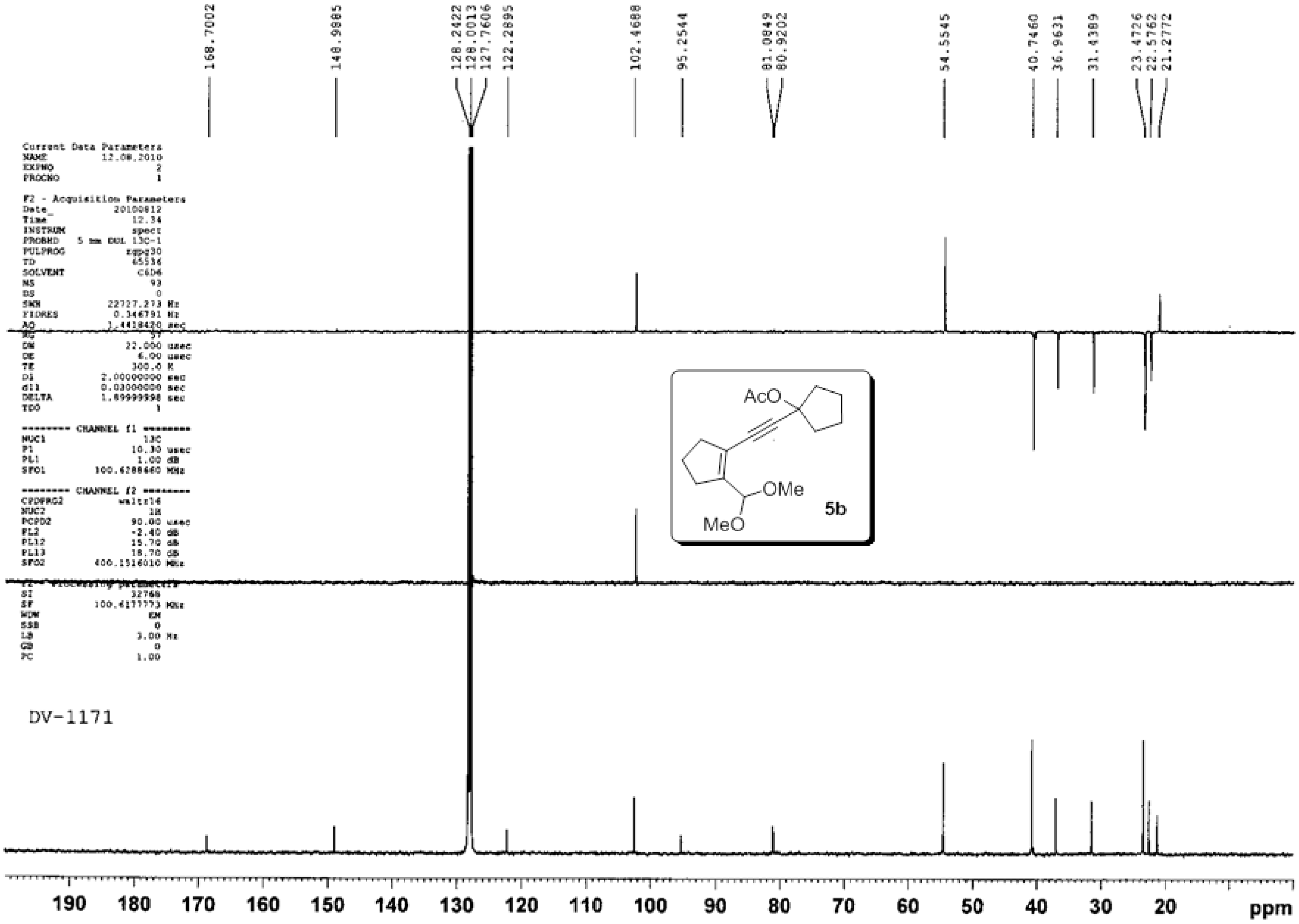
168.7002
148.9885
128.2422
128.0013
127.7606
122.2895
102.4688
95.2544
81.0849
80.9202
54.5545
40.7460
36.9631
31.4389
23.4726
22.5762
21.2772
Current Data Parameters
NAME 12.08.2010
EXPNO 2
PROCNO 1
F2 - Acquisition Parameters
Date_ 20100812
Time 12.34
INSTRUM spect
PROBHD 5 mm DUL 13C-1
PULPROG zgpg30
TD 65536
SOLVENT C6D6
NS 93
DS 0
SWH 22727.273 Hz
FIDRES 0.346791 Hz
AQ 1.4418420 sec
DW 22.000 usec
DE 6.00 usec
TE 300.0 K
D1 2.00000000 sec
d11 0.03000000 sec
DELTA 1.89999998 sec
TD0 1
======== CHANNEL f1 ========
NUC1 13C
P1 10.30 usec
PL1 1.00 dB
SFO1 100.6288660 MHz
======== CHANNEL f2 ========
CPDPRG2 waltz16
NUC2 1H
PCPD2 90.00 usec
PL2 -2.40 dB
PL12 15.70 dB
PL13 18.70 dB
SFO2 400.1516010 MHz
SI 32768
SF 100.6177773 MHz
WDW EM
SSB 0
LB 3.00 Hz
GB 0
PC 1.00
AcO
OMe
MeO
5b
DV-1171
190 180 170 160 150 140 130 120 110 100 90 80 70 60 50 40 30 20 ppm

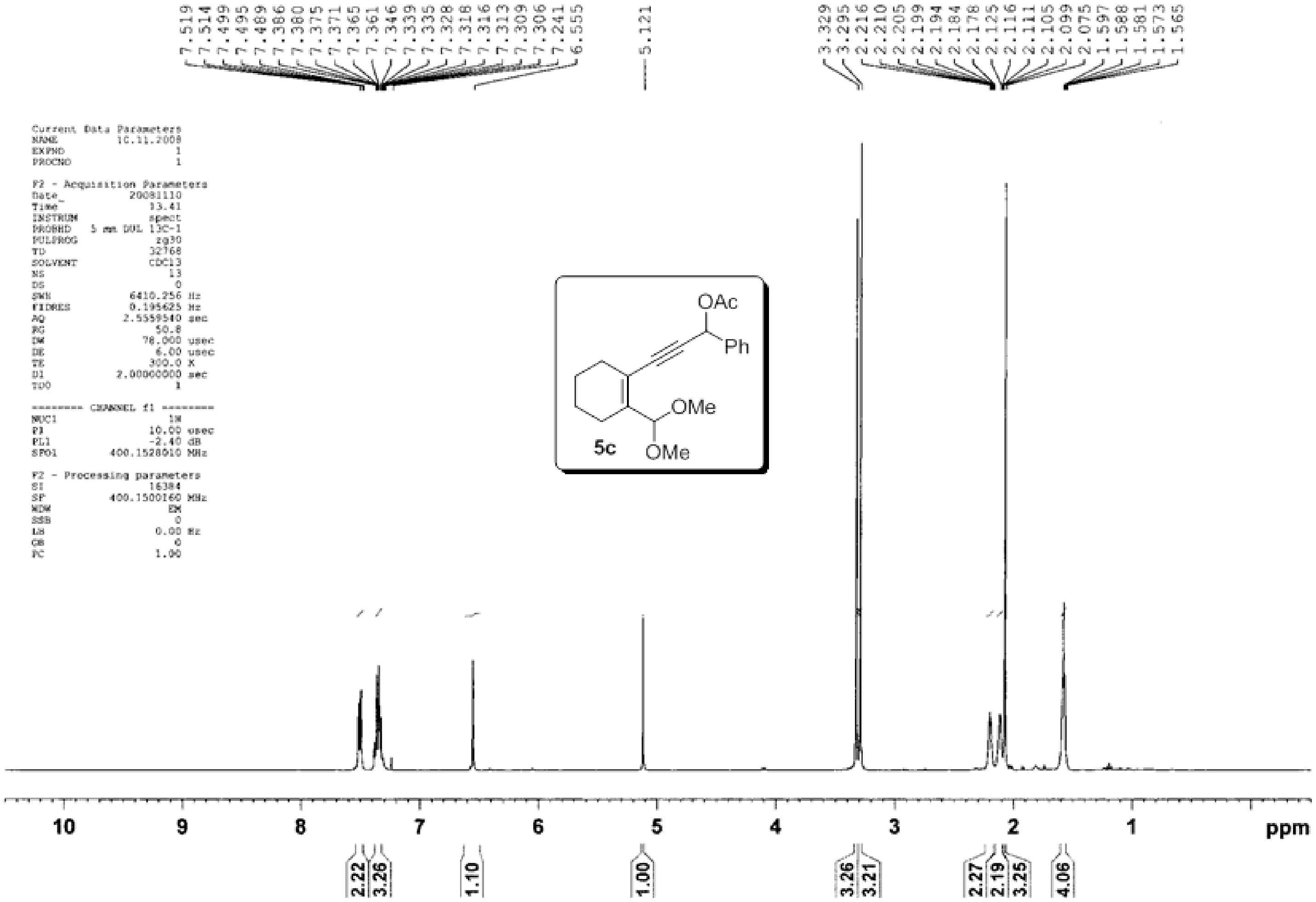

7.519 7.514 7.499 7.495 7.489 7.386 7.380 7.375 7.371 7.365 7.361 7.346 7.339 7.335 7.328 7.318 7.316 7.313 7.309 7.306 7.241 6.555
5.121
3.329 3.295 2.216 2.210 2.205 2.199 2.194 2.184 2.178 2.125 2.116 2.111 2.105 2.099 2.075 1.597 1.588 1.581 1.573 1.565
OAc
Ph
OMe
OMe
5c
10 9 8 7 6 5 4 3 2 1 ppm
2.22 3.26 1.10 1.00 3.26 3.21 2.27 2.19 3.25 4.06

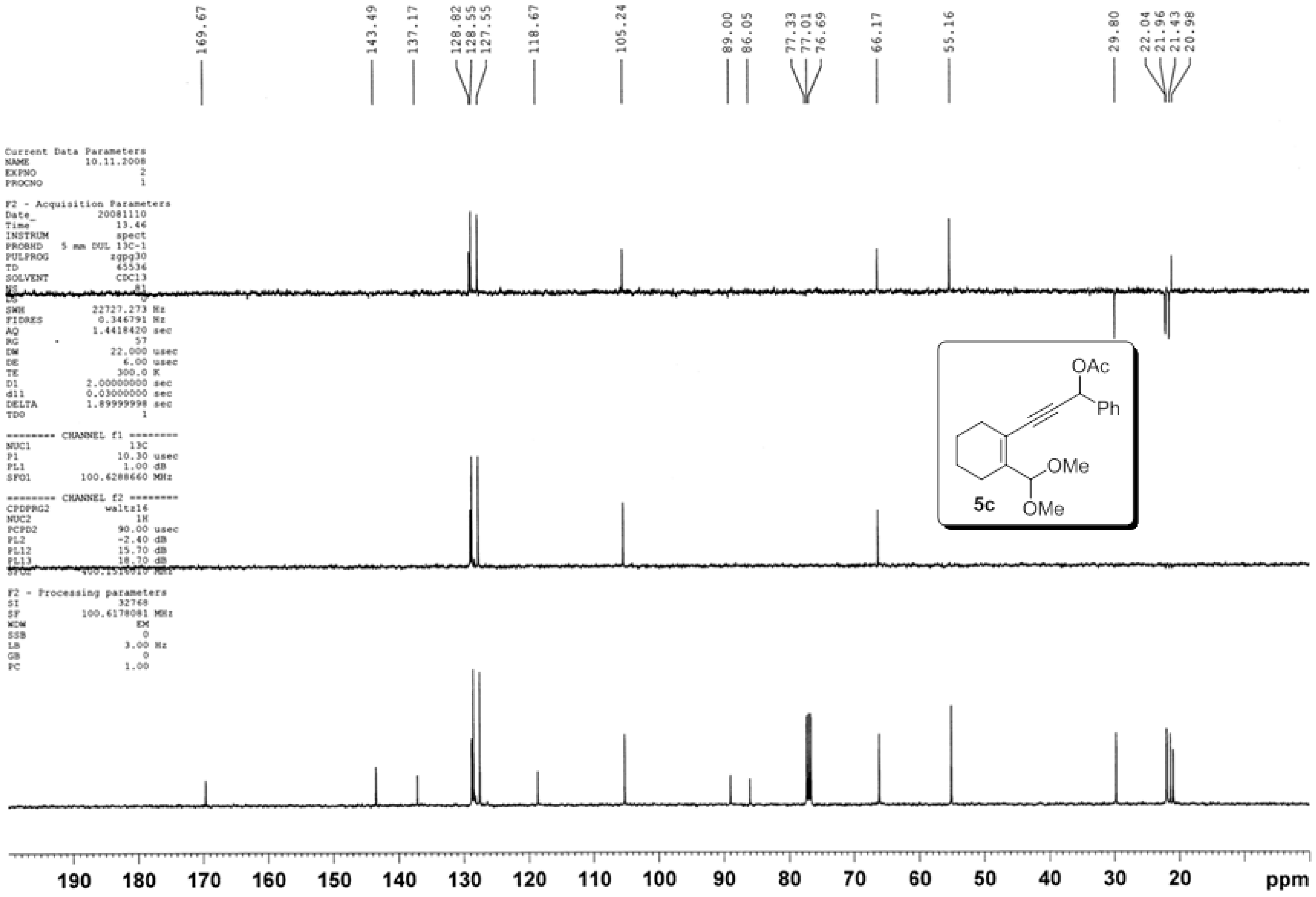

169.67
143.49
137.17
128.82
128.55
127.55
118.67
105.24
89.00
86.05
77.33
77.01
76.69
66.17
55.16
29.80
22.04
21.96
21.43
20.98
Current Data Parameters
NAME 10.11.2008
EXPNO 2
PROCNO 1
F2 - Acquisition Parameters
Date_ 20081110
Time 13.46
INSTRUM spect
PROBHD 5 mm DUL 13C-1
PULPROG zgpg30
TD 65536
SOLVENT CDCl3
SWH 22727.273 Hz
FIDRES 0.346791 Hz
AQ 1.4418420 sec
RG 57
DW 22.000 usec
DE 6.00 usec
TE 300.0 K
D1 2.00000000 sec
d11 0.03000000 sec
DELTA 1.89999998 sec
TD0 1
======== CHANNEL f1 ========
NUC1 13C
P1 10.30 usec
PL1 1.00 dB
SFO1 100.6288660 MHz
======== CHANNEL f2 ========
CPDPRG2 waltz16
NUC2 1H
PCPD2 90.00 usec
PL2 -2.40 dB
PL12 15.70 dB
PL13 18.70 dB
F2 - Processing parameters
SI 32768
SF 100.6178081 MHz
WDW EM
SSB 0
LB 3.00 Hz
GB 0
PC 1.00
OAc
Ph
OMe
OMe
5c
190 180 170 160 150 140 130 120 110 100 90 80 70 60 50 40 30 20 ppm

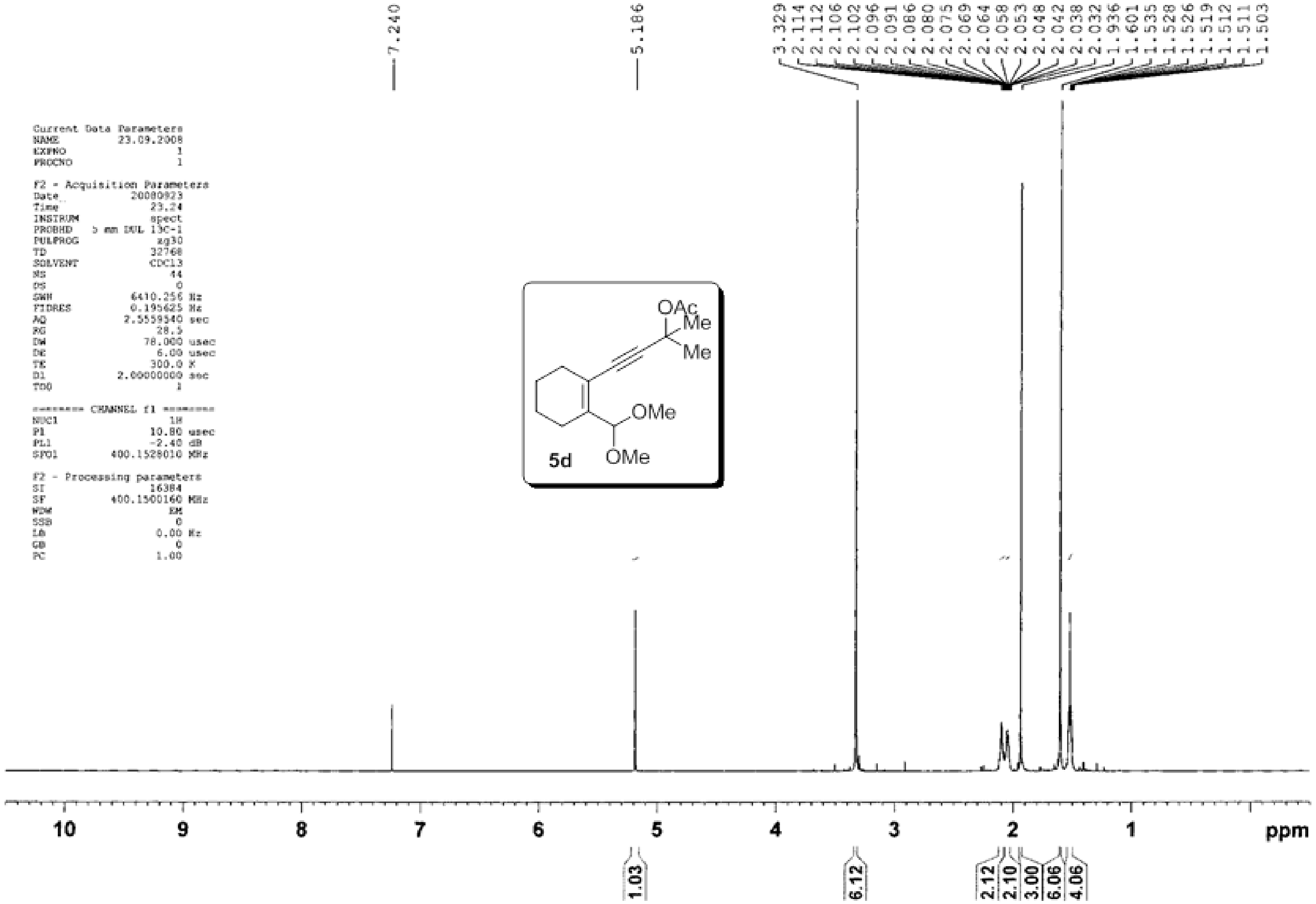
Current Data Parameters
NAME 23.09.2008
EXPNO 1
PROCNO 1
F2 - Acquisition Parameters
Date_ 20080923
Time 23.24
INSTRUM spect
PROBHD 5 mm DUL 13C-1
PULPROG zg30
TD 32768
SOLVENT CDCl3
NS 44
DS 0
SWH 6410.256 Hz
FIDRES 0.195625 Hz
AQ 2.5559540 sec
RG 28.5
DW 78.000 usec
DE 6.00 usec
TE 300.0 K
D1 2.00000000 sec
TD0 1
======== CHANNEL f1 ========
NUC1 1H
P1 10.80 usec
PL1 -2.40 dB
SFO1 400.1528010 MHz
F2 - Processing parameters
SI 16384
SF 400.1500160 MHz
WDW EM
SSB 0
LB 0.00 Hz
GB 0
PC 1.00
OAc
Me
Me
OMe
OMe
5d
7.240
5.186
3.329
2.114
2.112
2.106
2.102
2.096
2.091
2.086
2.080
2.075
2.069
2.064
2.058
2.053
2.048
2.042
2.038
2.032
1.936
1.601
1.535
1.528
1.526
1.519
1.512
1.511
1.503
10
9
8
7
6
5
4
3
2
1
ppm
1.03
6.12
2.12
2.10
3.00
6.06
4.06

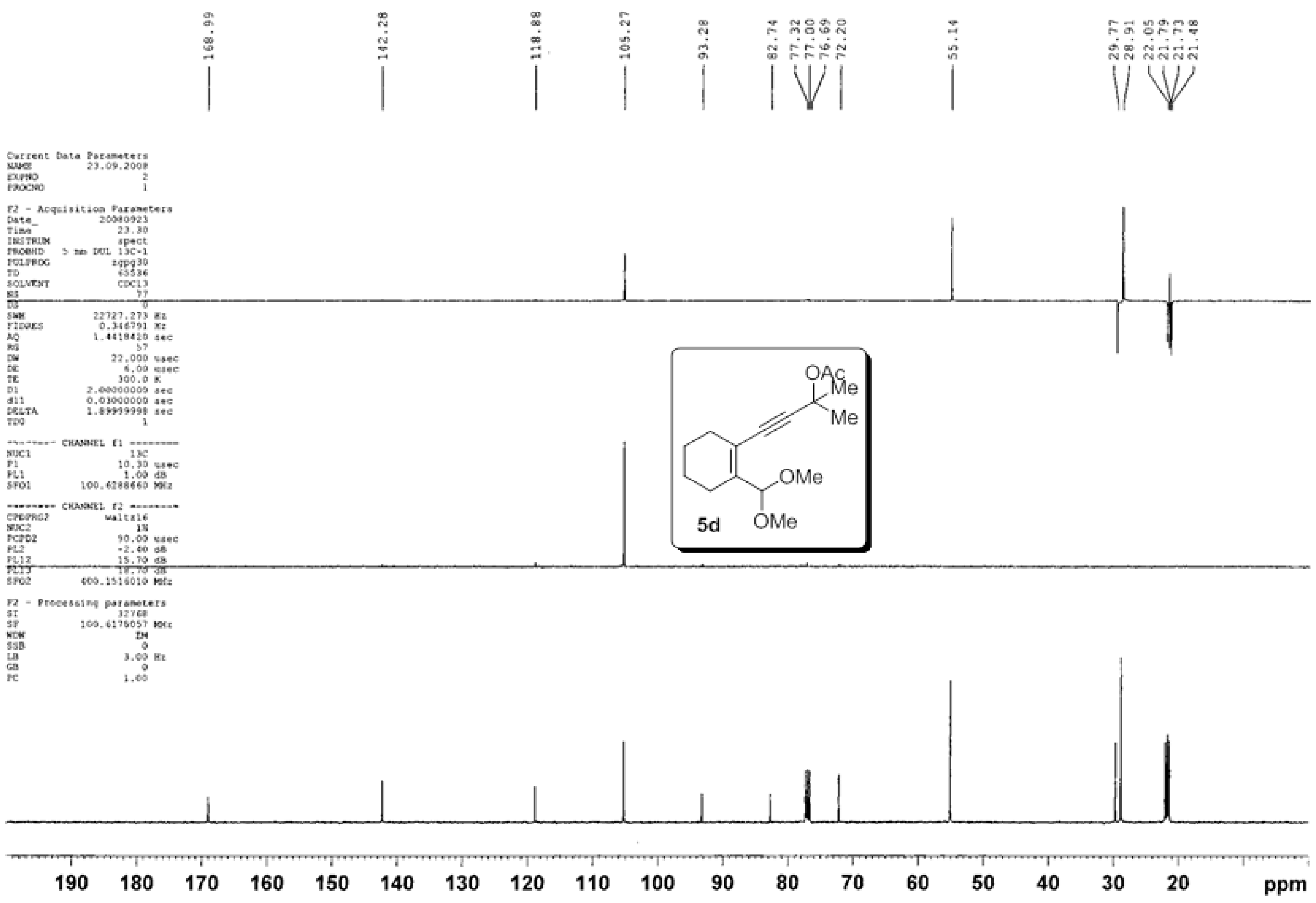
168.99
142.28
118.88
105.27
93.28
82.74
77.32
77.00
76.69
72.20
55.14
29.77
28.91
22.05
21.79
21.73
21.48
OAc
Me
Me
OMe
OMe
5d
190 180 170 160 150 140 130 120 110 100 90 80 70 60 50 40 30 20 ppm

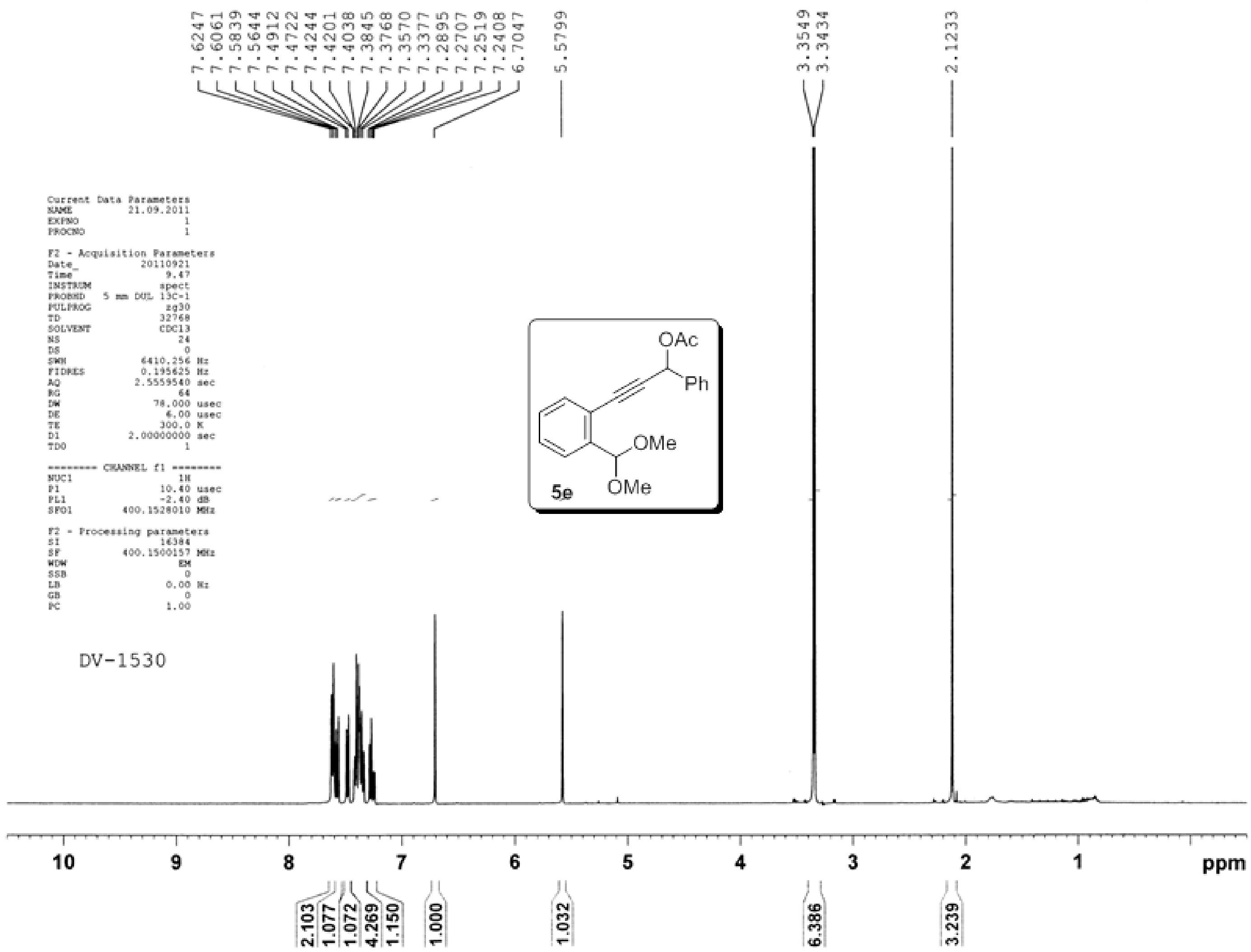
7.6247
7.6061
7.5839
7.5644
7.4912
7.4722
7.4244
7.4201
7.4038
7.3845
7.3768
7.3570
7.3377
7.2895
7.2707
7.2519
7.2408
6.7047
5.5799
3.3549
3.3434
2.1233
Current Data Parameters
NAME 21.09.2011
EXPNO 1
PROCNO 1
F2 - Acquisition Parameters
Date_ 20110921
Time 9.47
INSTRUM spect
PROBHD 5 mm DUL 13C-1
PULPROG zg30
TD 32768
SOLVENT CDCl3
NS 24
DS 0
SWH 6410.256 Hz
FIDRES 0.195625 Hz
AQ 2.5559540 sec
RG 64
DW 78.000 usec
DE 6.00 usec
TE 300.0 K
D1 2.00000000 sec
TD0 1
======== CHANNEL f1 ========
NUC1 1H
P1 10.40 usec
PL1 -2.40 dB
SFO1 400.1528010 MHz
F2 - Processing parameters
SI 16384
SF 400.1500157 MHz
WDW EM
SSB 0
LB 0.00 Hz
GB 0
PC 1.00
DV-1530
OAc
Ph
OMe
OMe
5e
10
9
8
7
6
5
4
3
2
1
ppm
2.103
1.077
1.072
4.269
1.150
1.000
1.032
6.386
3.239

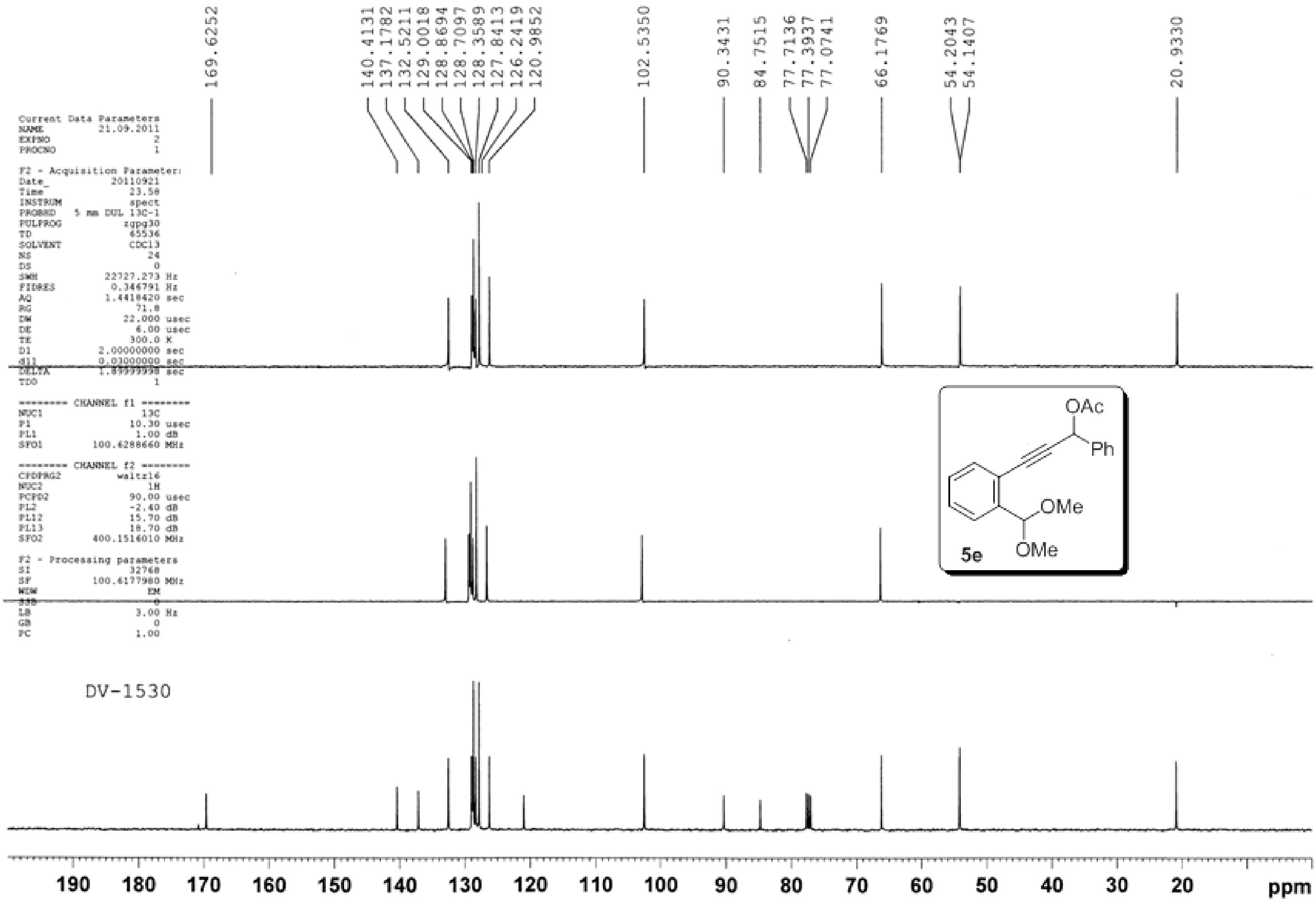
169.6252
140.4131
137.1782
132.5211
129.0018
128.8694
128.7097
128.3589
127.8413
126.2419
120.9852
102.5350
90.3431
84.7515
77.7136
77.3937
77.0741
66.1769
54.2043
54.1407
20.9330
Current Data Parameters
NAME 21.09.2011
EXPNO 2
PROCNO 1
F2 - Acquisition Parameters
Date_ 20110921
Time 23.58
INSTRUM spect
PROBHD 5 mm DUL 13C-1
PULPROG zgpg30
TD 65536
SOLVENT CDCl3
NS 24
DS 0
SWH 22727.273 Hz
FIDRES 0.346791 Hz
AQ 1.4418420 sec
RG 71.8
DW 22.000 usec
DE 6.00 usec
TE 300.0 K
D1 2.00000000 sec
d11 0.03000000 sec
DELTA 1.89999998 sec
TD0 1
======== CHANNEL f1 ========
NUC1 13C
P1 10.30 usec
PL1 1.00 dB
SFO1 100.6288660 MHz
======== CHANNEL f2 ========
CPDPRG2 waltz16
NUC2 1H
PCPD2 90.00 usec
PL2 -2.40 dB
PL12 15.70 dB
PL13 18.70 dB
SFO2 400.1516010 MHz
F2 - Processing parameters
SI 32768
SF 100.6177980 MHz
WDW EM
SSB 0
LB 3.00 Hz
GB 0
PC 1.00
DV-1530
OAc
Ph
OMe
OMe
5e
190 180 170 160 150 140 130 120 110 100 90 80 70 60 50 40 30 20 ppm

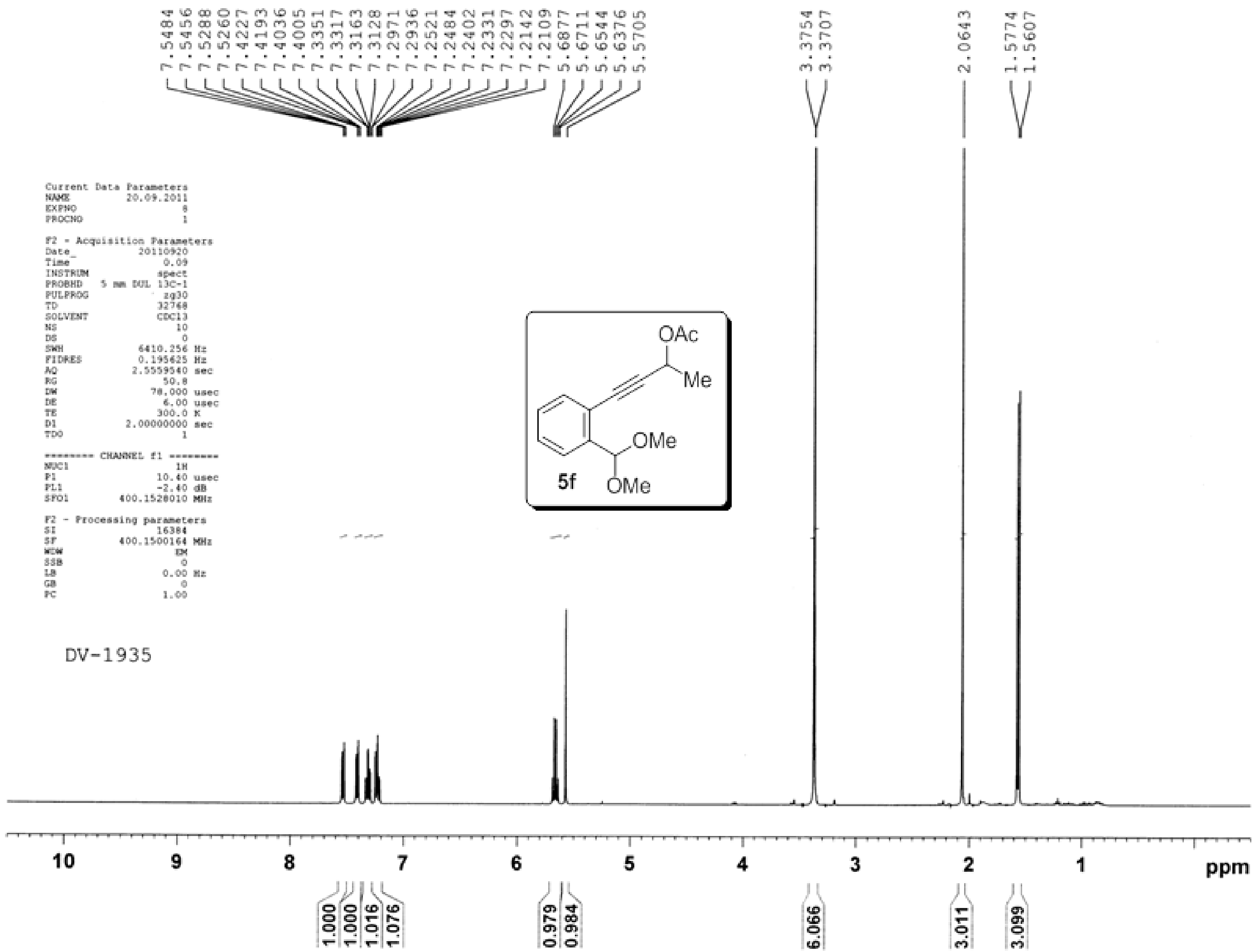

7.5484
7.5456
7.5288
7.5260
7.4227
7.4193
7.4036
7.4005
7.3351
7.3317
7.3163
7.3128
7.2971
7.2936
7.2521
7.2484
7.2402
7.2331
7.2297
7.2142
7.2109
5.6877
5.6711
5.6544
5.6376
5.5705
3.3754
3.3707
2.0643
1.5774
1.5607
Current Data Parameters
NAME 20.09.2011
EXPNO 8
PROCNO 1
F2 - Acquisition Parameters
Date_ 20110920
Time 0.09
INSTRUM spect
PROBHD 5 mm DUL 13C-1
PULPROG zg30
TD 32768
SOLVENT CDCl3
NS 10
DS 0
SWH 6410.256 Hz
FIDRES 0.195625 Hz
AQ 2.5559540 sec
RG 50.8
DW 78.000 usec
DE 6.00 usec
TE 300.0 K
D1 2.00000000 sec
TD0 1
======== CHANNEL f1 ========
NUC1 1H
P1 10.40 usec
PL1 -2.40 dB
SFO1 400.1528010 MHz
F2 - Processing parameters
SI 16384
SF 400.1500164 MHz
WDW EM
SSB 0
LB 0.00 Hz
GB 0
PC 1.00
DV-1935
OAc
Me
OMe
OMe
5f
10
9
8
7
6
5
4
3
2
1
ppm
1.000
1.000
1.016
1.076
0.979
0.984
6.066
3.011
3.099

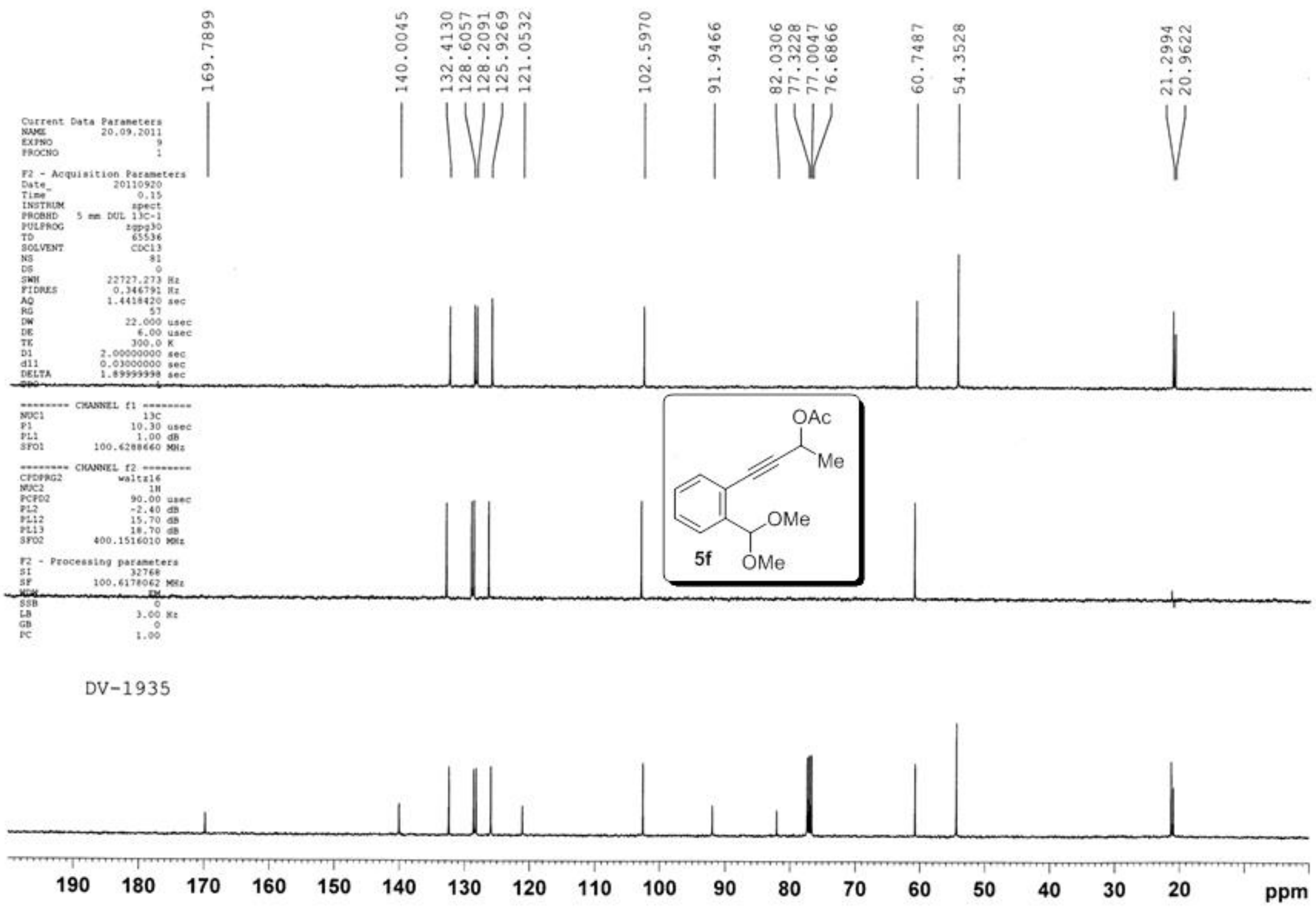
169.7899
140.0045
132.4130
128.6057
128.2091
125.9269
121.0532
102.5970
91.9466
82.0306
77.3228
77.0047
76.6866
60.7487
54.3528
21.2994
20.9622
Current Data Parameters
NAME 20.09.2011
EXPNO 9
PROCNO 1
F2 - Acquisition Parameters
Date_ 20110920
Time 0.15
INSTRUM spect
PROBHD 5 mm DUL 13C-1
PULPROG zgpg30
TD 65536
SOLVENT CDCl3
NS 81
DS 0
SWH 22727.273 Hz
FIDRES 0.346791 Hz
AQ 1.4418420 sec
RG 57
DW 22.000 usec
DE 6.00 usec
TE 300.0 K
D1 2.00000000 sec
d11 0.03000000 sec
DELTA 1.89999998 sec
======== CHANNEL f1 ========
NUC1 13C
P1 10.30 usec
PL1 1.00 dB
SFO1 100.6288660 MHz
======== CHANNEL f2 ========
CPDPRG2 waltz16
NUC2 1H
PCPD2 90.00 usec
PL2 -2.40 dB
PL12 15.70 dB
PL13 18.70 dB
SFO2 400.1516010 MHz
F2 - Processing parameters
SI 32768
SF 100.6178062 MHz
SSB 0
LB 3.00 Hz
GB 0
PC 1.00
OAc
Me
OMe
OMe
5f
DV-1935
190 180 170 160 150 140 130 120 110 100 90 80 70 60 50 40 30 20 ppm

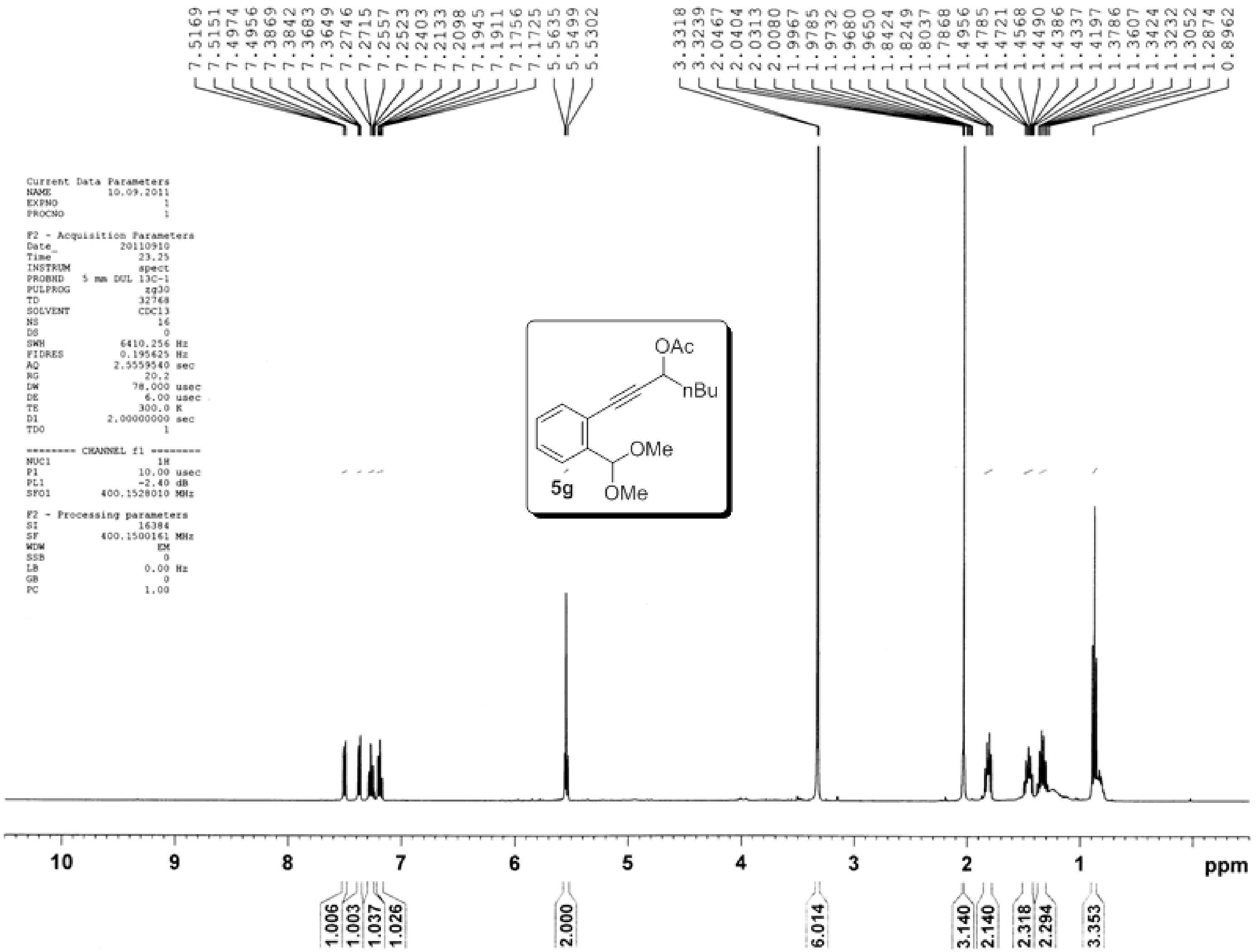
7.5169 7.5151 7.4974 7.4956 7.3869 7.3842 7.3683 7.3649 7.2746 7.2715 7.2557 7.2523 7.2403 7.2133 7.2098 7.1945 7.1911 7.1756 7.1725 5.5635 5.5499 5.5302
3.3318 3.3239 2.0467 2.0404 2.0313 2.0080 1.9967 1.9785 1.9732 1.9680 1.9650 1.8424 1.8249 1.8037 1.7868 1.4956 1.4785 1.4721 1.4568 1.4490 1.4386 1.4337 1.4197 1.3786 1.3607 1.3424 1.3232 1.3052 1.2874 0.8962
Current Data Parameters
NAME 10.09.2011
EXPNO 1
PROCNO 1
F2 - Acquisition Parameters
Date_ 20110910
Time 23.25
INSTRUM spect
PROBHD 5 mm DUL 13C-1
PULPROG zg30
TD 32768
SOLVENT CDCl3
NS 16
DS 0
SWH 6410.256 Hz
FIDRES 0.195625 Hz
AQ 2.5559540 sec
RG 20.2
DW 78.000 usec
DE 6.00 usec
TE 300.0 K
D1 2.00000000 sec
TD0 1
======== CHANNEL f1 ========
NUC1 1H
P1 10.00 usec
PL1 -2.40 dB
SFO1 400.1528010 MHz
F2 - Processing parameters
SI 16384
SF 400.1500161 MHz
WDW EM
SSB 0
LB 0.00 Hz
GB 0
PC 1.00
OAc
nBu
OMe
5g
OMe
10 9 8 7 6 5 4 3 2 1 ppm
1.006 1.003 1.037 1.026
2.000
6.014
3.140 2.140
2.318 2.294
3.353

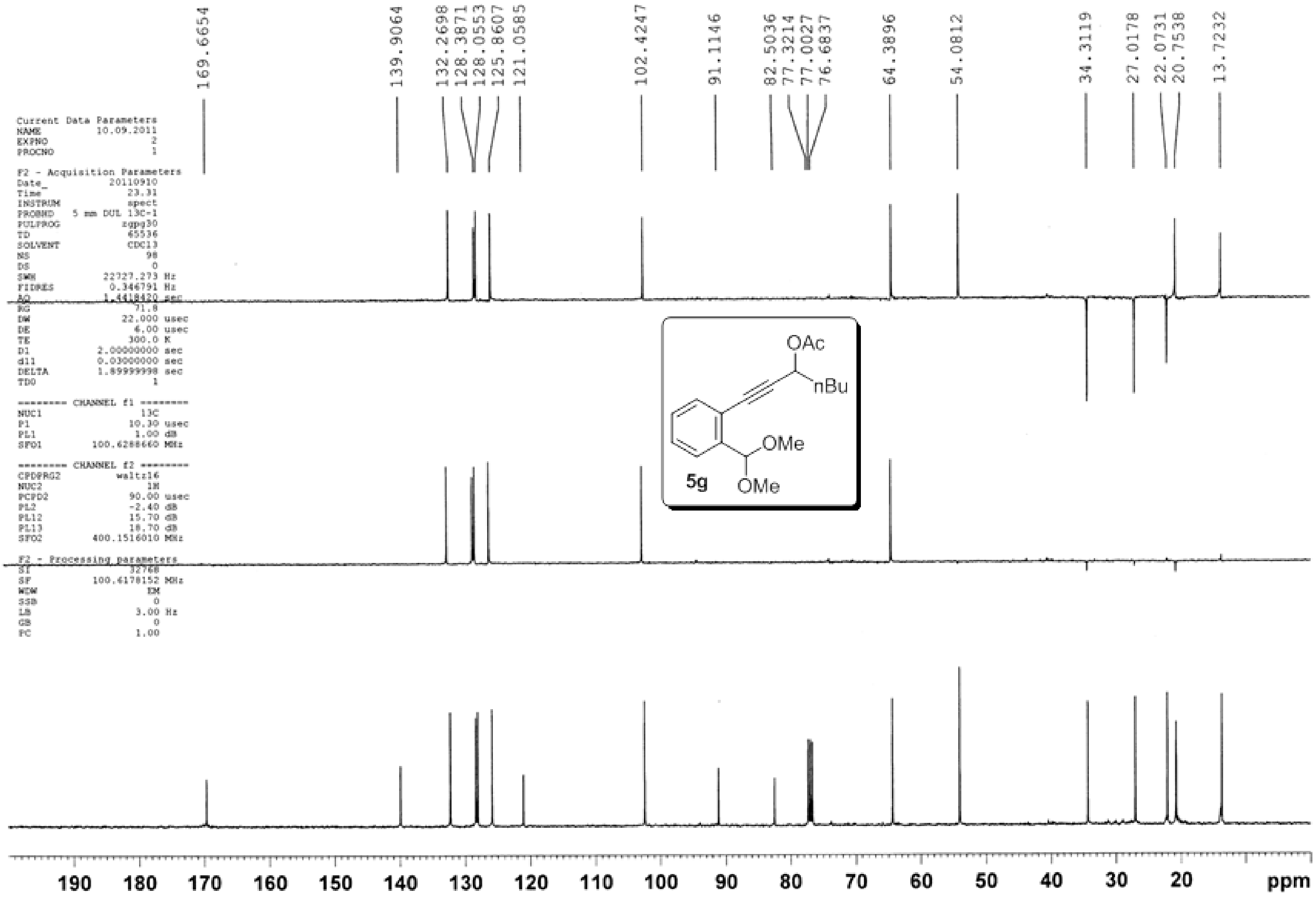
169.6654
139.9064
132.2698
128.3871
128.0553
125.8607
121.0585
102.4247
91.1146
82.5036
77.3214
77.0027
76.6837
64.3896
54.0812
34.3119
27.0178
22.0731
20.7538
13.7232
Current Data Parameters
NAME 10.09.2011
EXPNO 2
PROCNO 1
F2 - Acquisition Parameters
Date_ 20110910
Time 23.31
INSTRUM spect
PROBHD 5 mm DUL 13C-1
PULPROG zgpg30
TD 65536
SOLVENT CDCl3
NS 98
DS 0
SWH 22727.273 Hz
FIDRES 0.346791 Hz
AQ 1.4418420 sec
RG 71.8
DW 22.000 usec
DE 6.00 usec
TE 300.0 K
D1 2.00000000 sec
d11 0.03000000 sec
DELTA 1.89999998 sec
TD0 1
======== CHANNEL f1 ========
NUC1 13C
P1 10.30 usec
PL1 1.00 dB
SFO1 100.6288660 MHz
======== CHANNEL f2 ========
CPDPRG2 waltz16
NUC2 1H
PCPD2 90.00 usec
PL2 -2.40 dB
PL12 15.70 dB
PL13 18.70 dB
SFO2 400.1516010 MHz
F2 - Processing parameters
SI 32768
SF 100.6178152 MHz
WDW EM
SSB 0
LB 3.00 Hz
GB 0
PC 1.00
OAc
nBu
OMe
5g
OMe
190 180 170 160 150 140 130 120 110 100 90 80 70 60 50 40 30 20 ppm

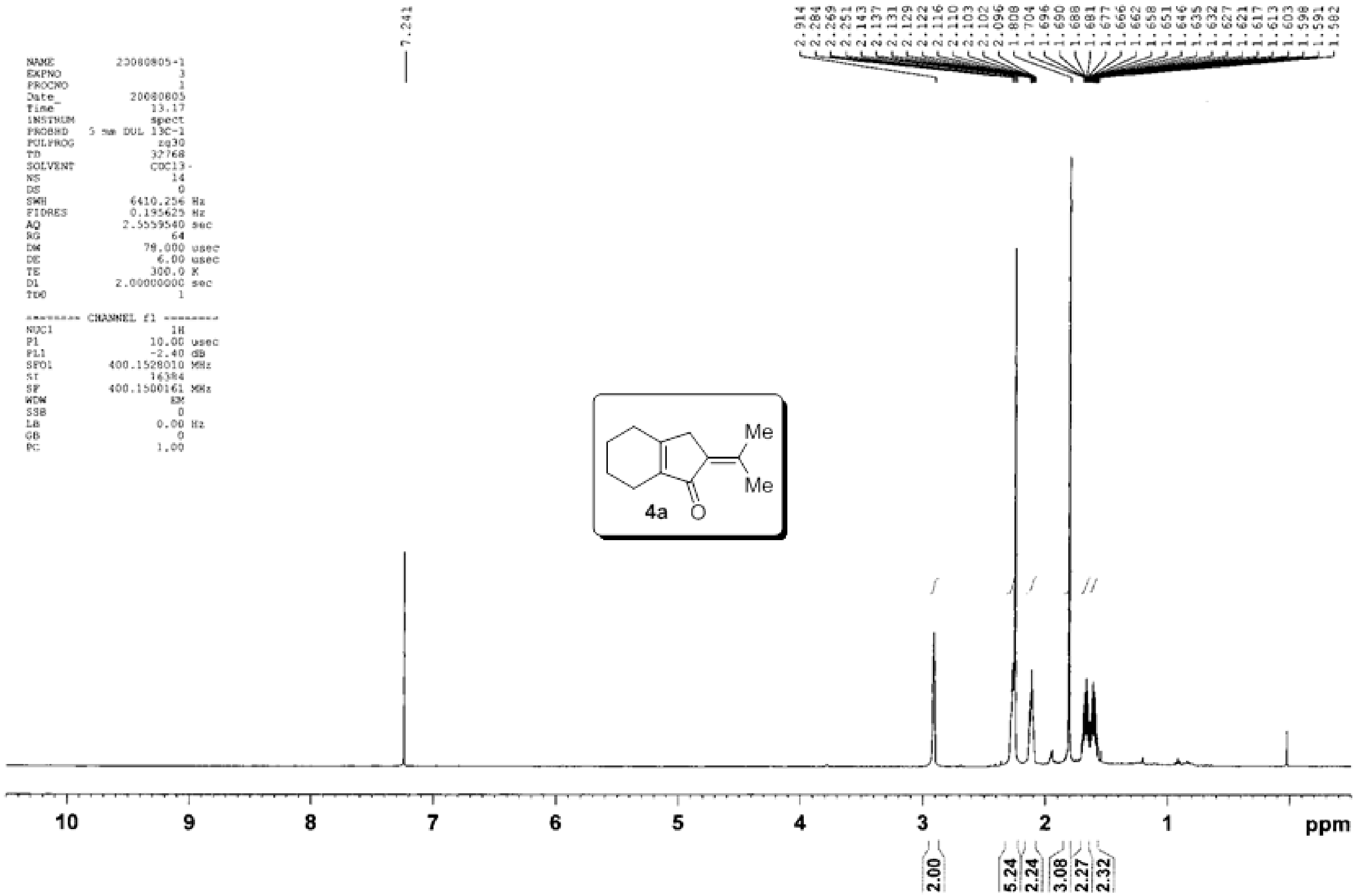

Me
Me
4a
ppm

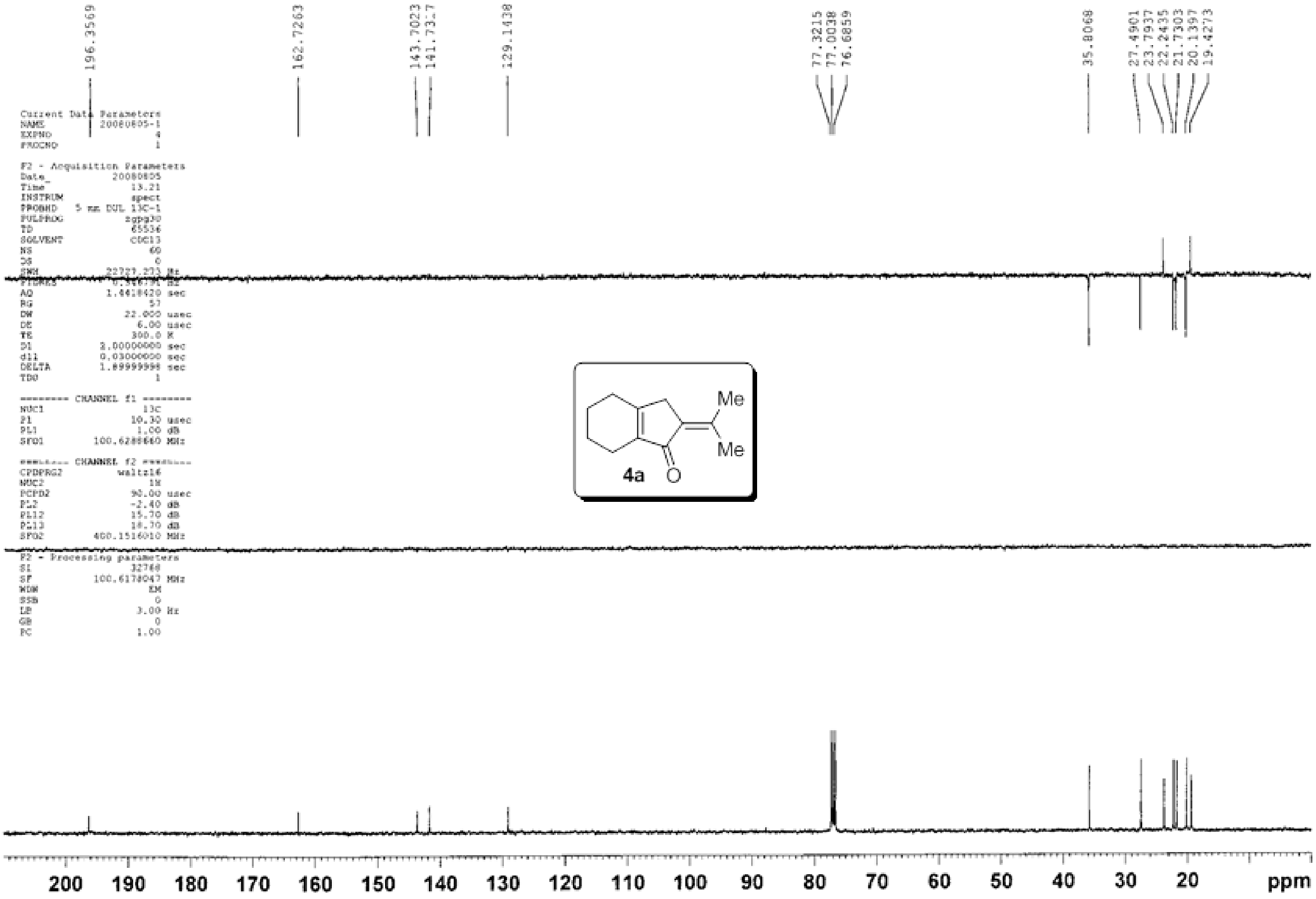
196.3569
162.7263
143.7023
141.7317
129.1438
77.3215
77.0038
76.6859
35.8068
27.4901
23.7937
22.2435
21.7303
20.1397
19.4273
Me
Me
4a
O
200 190 180 170 160 150 140 130 120 110 100 90 80 70 60 50 40 30 20 ppm

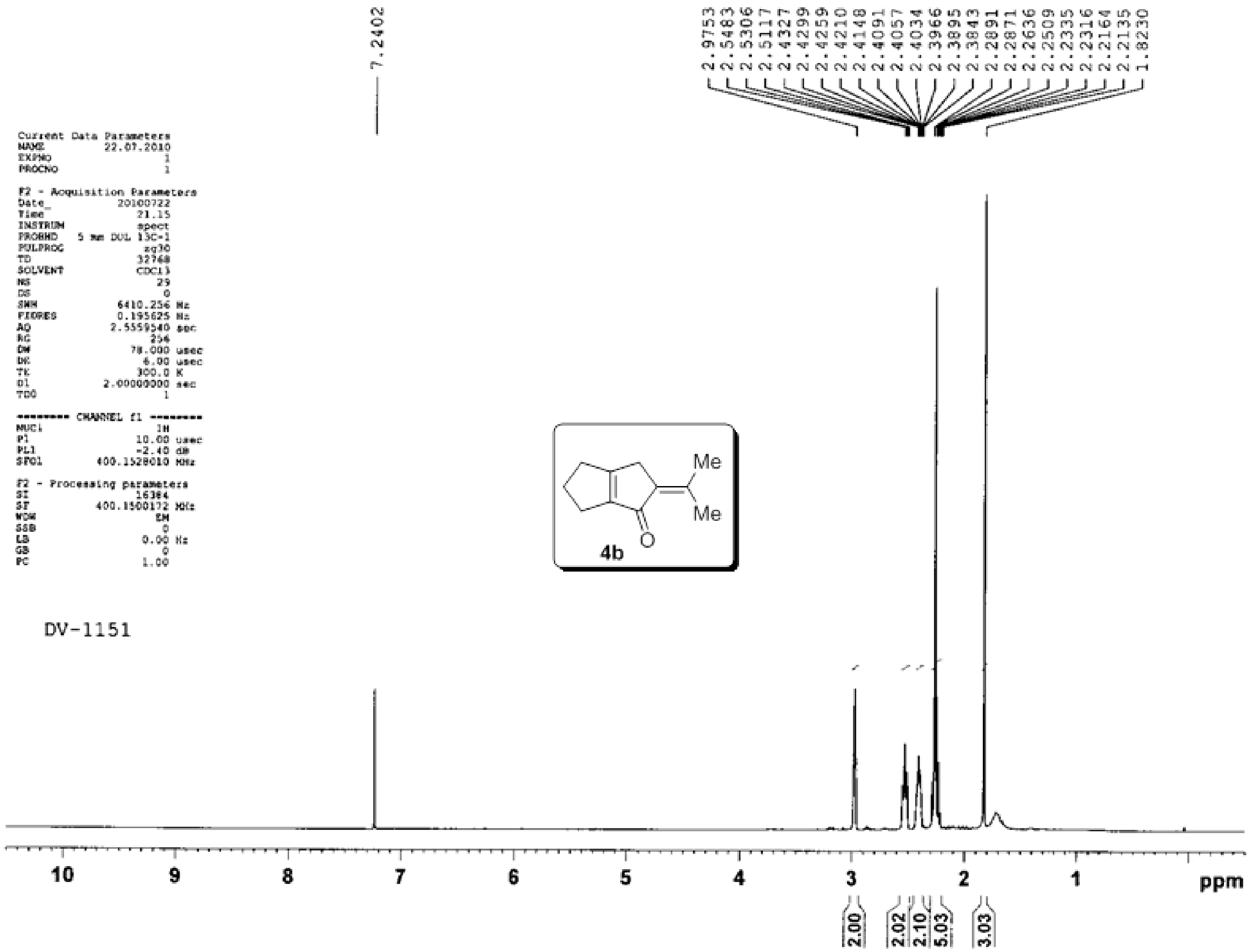
Current Data Parameters
NAME 22.07.2010
EXPNO 1
PROCNO 1
F2 - Acquisition Parameters
Date_ 20100722
Time 21.15
INSTRUM spect
PROBHD 5 mm DUL 13C-1
PULPROG zg30
TD 32768
SOLVENT CDCl3
NS 29
DS 0
SWH 6410.256 Hz
FIDRES 0.195625 Hz
AQ 2.5559540 sec
RG 256
DW 78.000 usec
DE 6.00 usec
TE 300.0 K
D1 2.00000000 sec
TD0 1
======== CHANNEL f1 ========
NUC1 1H
P1 10.00 usec
PL1 -2.40 dB
SFO1 400.1528010 MHz
F2 - Processing parameters
SI 16384
SF 400.1500172 MHz
WDW EM
SSB 0
LB 0.00 Hz
GB 0
PC 1.00
DV-1151
7.2402
2.9753
2.5483
2.5306
2.5117
2.4327
2.4299
2.4259
2.4210
2.4148
2.4091
2.4057
2.4034
2.3966
2.3895
2.3843
2.2891
2.2871
2.2636
2.2509
2.2335
2.2316
2.2164
2.2135
1.8230
Me
Me
O
4b
10
9
8
7
6
5
4
3
2
1
ppm
2.00
2.02
2.10
5.03
3.03

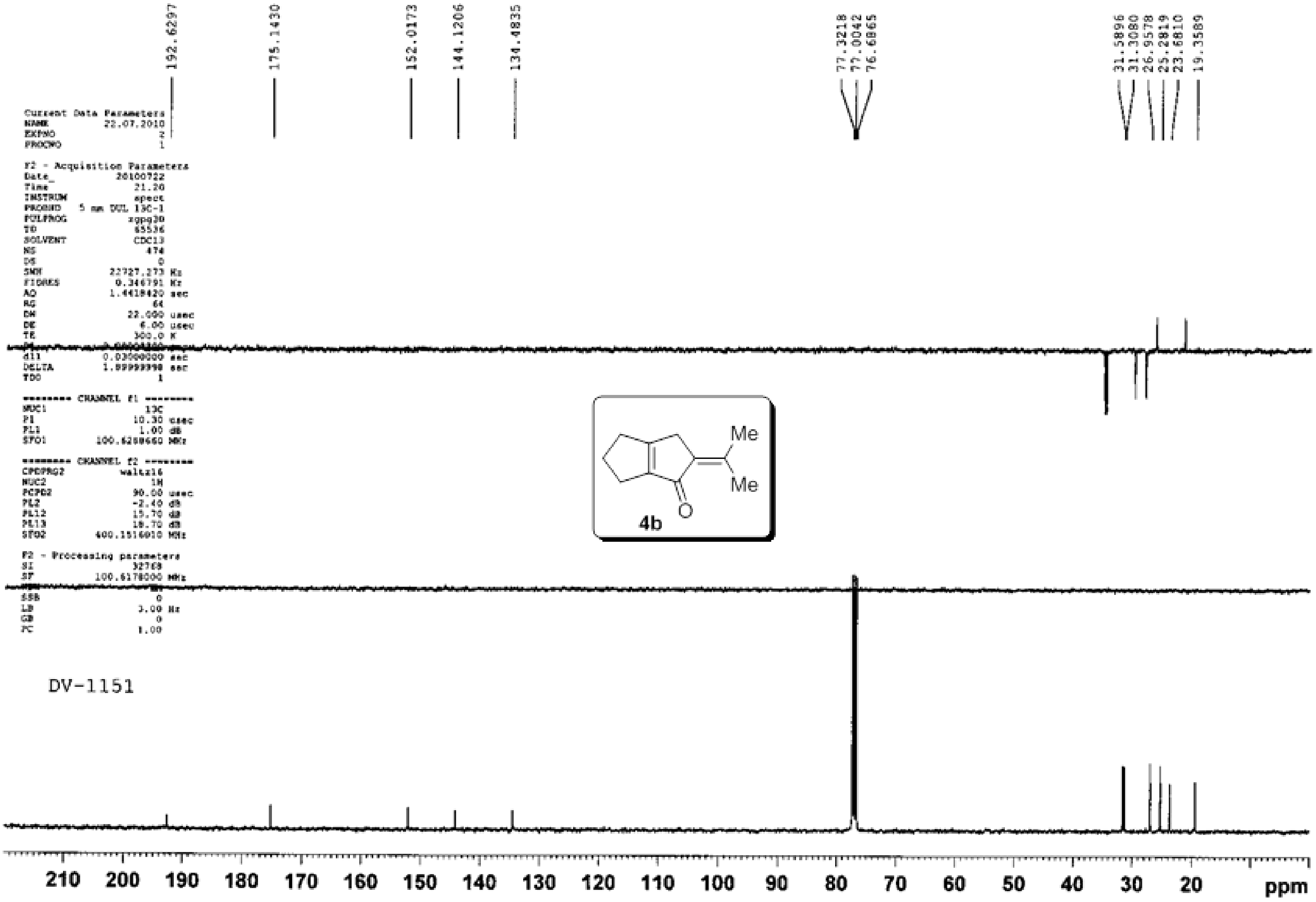
192.6297
175.1430
152.0173
144.1206
134.4835
77.3218
77.0042
76.6865
31.5896
31.3080
26.9578
25.2819
23.6810
19.3589
Me
Me
O
4b
DV-1151
210 200 190 180 170 160 150 140 130 120 110 100 90 80 70 60 50 40 30 20 ppm

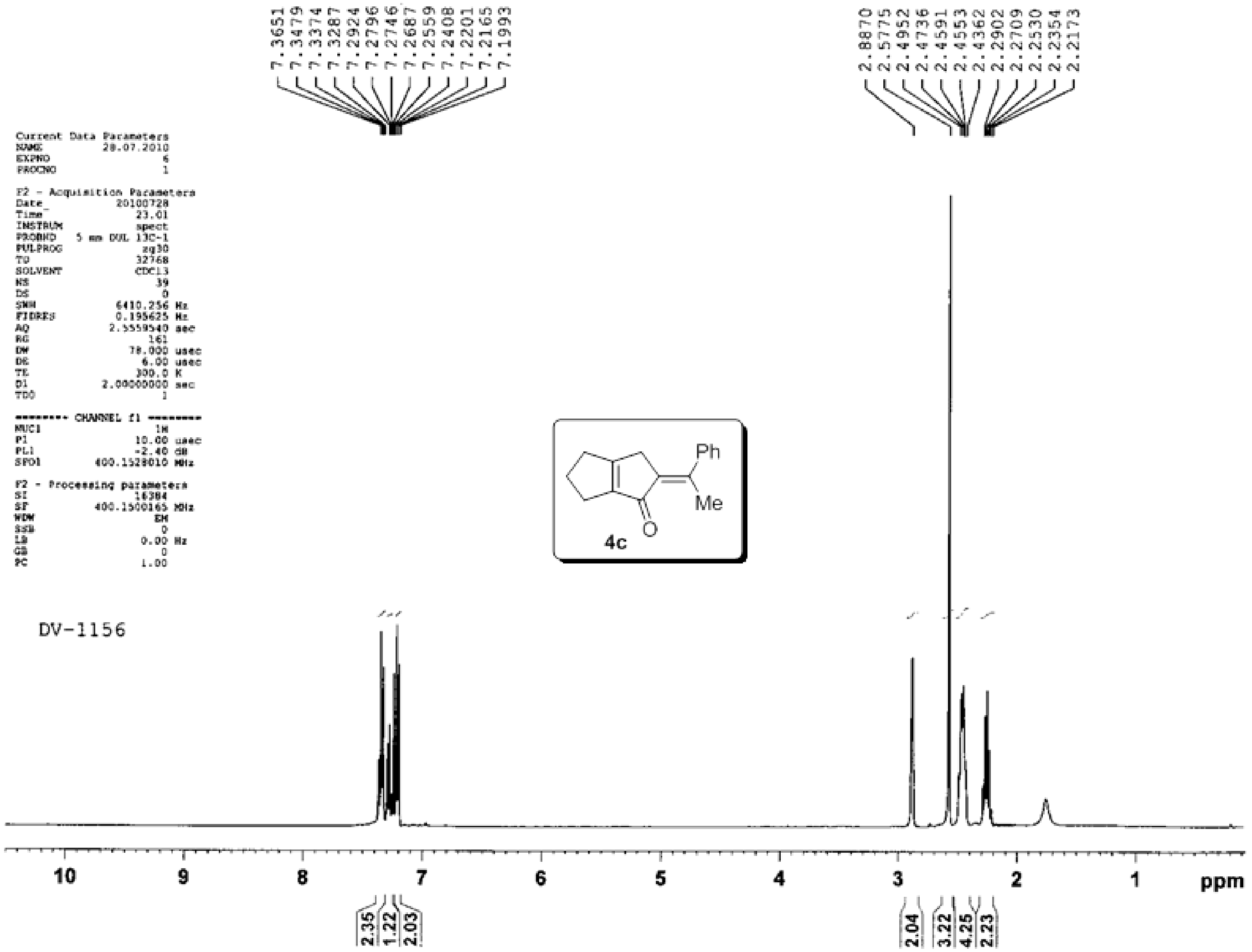

7.3651 7.3479 7.3374 7.3287 7.2924 7.2796 7.2746 7.2687 7.2559 7.2408 7.2201 7.2165 7.1993
2.8870 2.5775 2.4952 2.4736 2.4591 2.4553 2.4362 2.2902 2.2709 2.2530 2.2354 2.2173
Ph
Me
O
4c
DV-1156
10 9 8 7 6 5 4 3 2 1 ppm
2.35 1.22 2.03
2.04 3.22 4.25 2.23

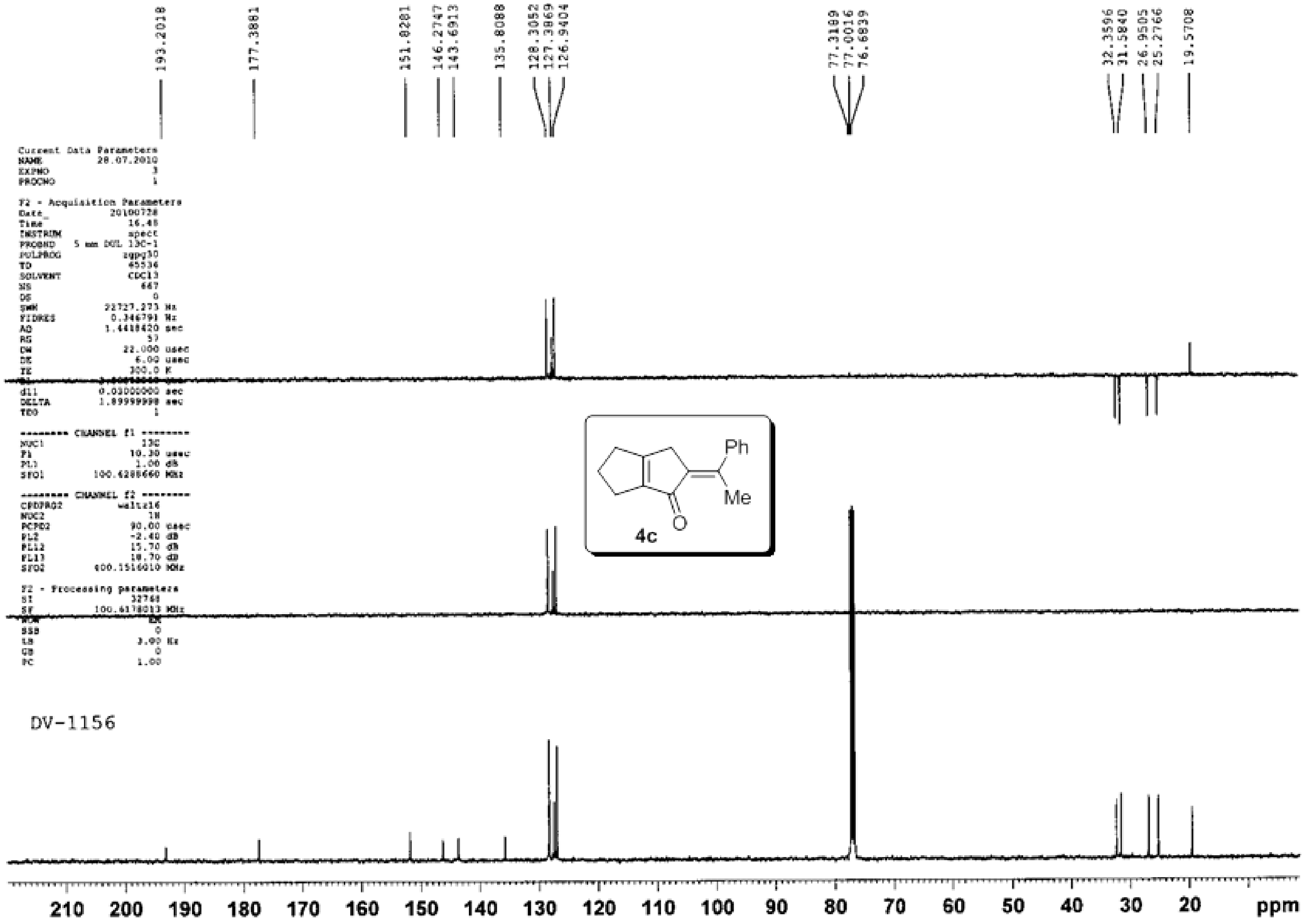
193.2018
177.3881
151.8281
146.2747
143.6913
135.8088
128.3052
127.3869
126.9404
77.3189
77.0016
76.6839
32.3596
31.5840
26.9505
25.2766
19.5708
Current Data Parameters
NAME 28.07.2010
EXPNO 3
PROCNO 1
F2 - Acquisition Parameters
Date_ 20100728
Time 16.48
INSTRUM spect
PULPROG zgpg30
TD 65536
SOLVENT CDCl3
NS 667
DS 0
SWH 22727.273 Hz
FIDRES 0.346791 Hz
AQ 1.4418420 sec
RG 57
DW 22.000 usec
DE 6.00 usec
TE 300.0 K
d11 0.03000000 sec
DELTA 1.89999998 sec
TD0 1
======== CHANNEL f1 ========
NUC1 13C
P1 10.30 usec
PL1 1.00 dB
SFO1 100.6288660 MHz
======== CHANNEL f2 ========
CPDPRG2 waltz16
NUC2 1H
PCPD2 90.00 usec
PL2 -2.40 dB
PL12 15.70 dB
PL13 18.70 dB
SFO2 400.1516010 MHz
F2 - Processing parameters
SI 32768
SF 100.6178013 MHz
SSB 0
LB 3.00 Hz
GB 0
PC 1.00
DV-1156
Ph
Me
O
4c
210 200 190 180 170 160 150 140 130 120 110 100 90 80 70 60 50 40 30 20 ppm

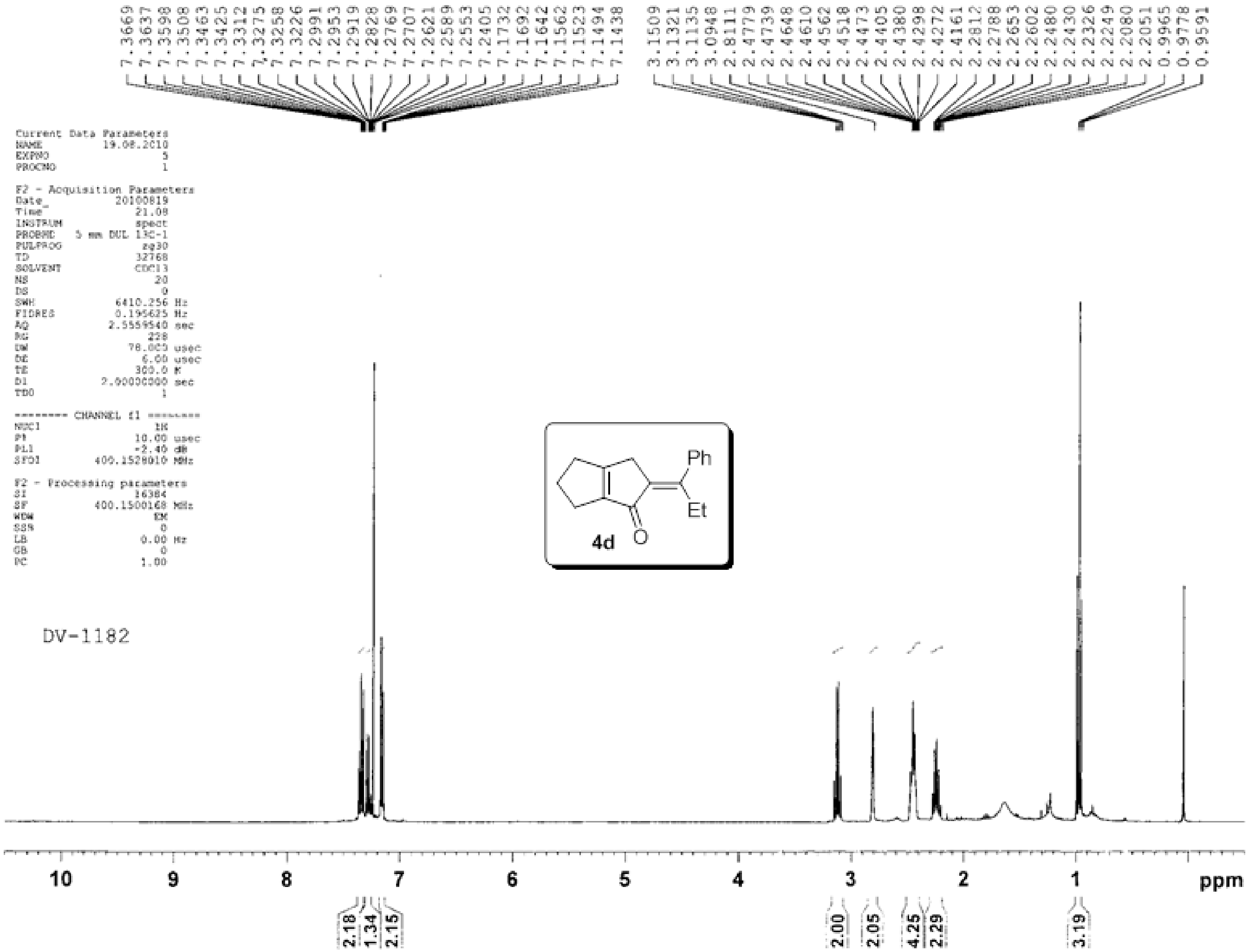
Current Data Parameters
NAME 19.08.2010
EXPNO 5
PROCNO 1
F2 - Acquisition Parameters
Date_ 20100819
Time 21.08
INSTRUM spect
PROBHD 5 mm DUL 13C-1
PULPROG zg30
TD 32768
SOLVENT CDCl3
NS 20
DS 0
SWH 6410.256 Hz
FIDRES 0.195625 Hz
AQ 2.5559540 sec
RG 228
DW 78.000 usec
DE 6.00 usec
TE 300.0 K
D1 2.00000000 sec
TD0 1
======== CHANNEL f1 ========
NUC1 1H
P1 10.00 usec
PL1 -2.40 dB
SFO1 400.1528010 MHz
F2 - Processing parameters
SI 16384
SF 400.1500168 MHz
WDW EM
SSB 0
LB 0.00 Hz
GB 0
PC 1.00
DV-1182
Ph
Et
O
4d
7.3669
7.3637
7.3598
7.3508
7.3463
7.3425
7.3312
7.3275
7.3258
7.3226
7.2991
7.2953
7.2919
7.2828
7.2769
7.2707
7.2621
7.2589
7.2553
7.2405
7.1732
7.1692
7.1642
7.1562
7.1523
7.1494
7.1438
3.1509
3.1321
3.1135
3.0948
2.8111
2.4779
2.4739
2.4648
2.4610
2.4562
2.4518
2.4473
2.4405
2.4380
2.4298
2.4272
2.4161
2.2812
2.2788
2.2653
2.2602
2.2480
2.2430
2.2326
2.2249
2.2080
2.2051
0.9965
0.9778
0.9591
10
9
8
7
6
5
4
3
2
1
ppm
2.18
1.34
2.15
2.00
2.05
4.25
2.29
3.19

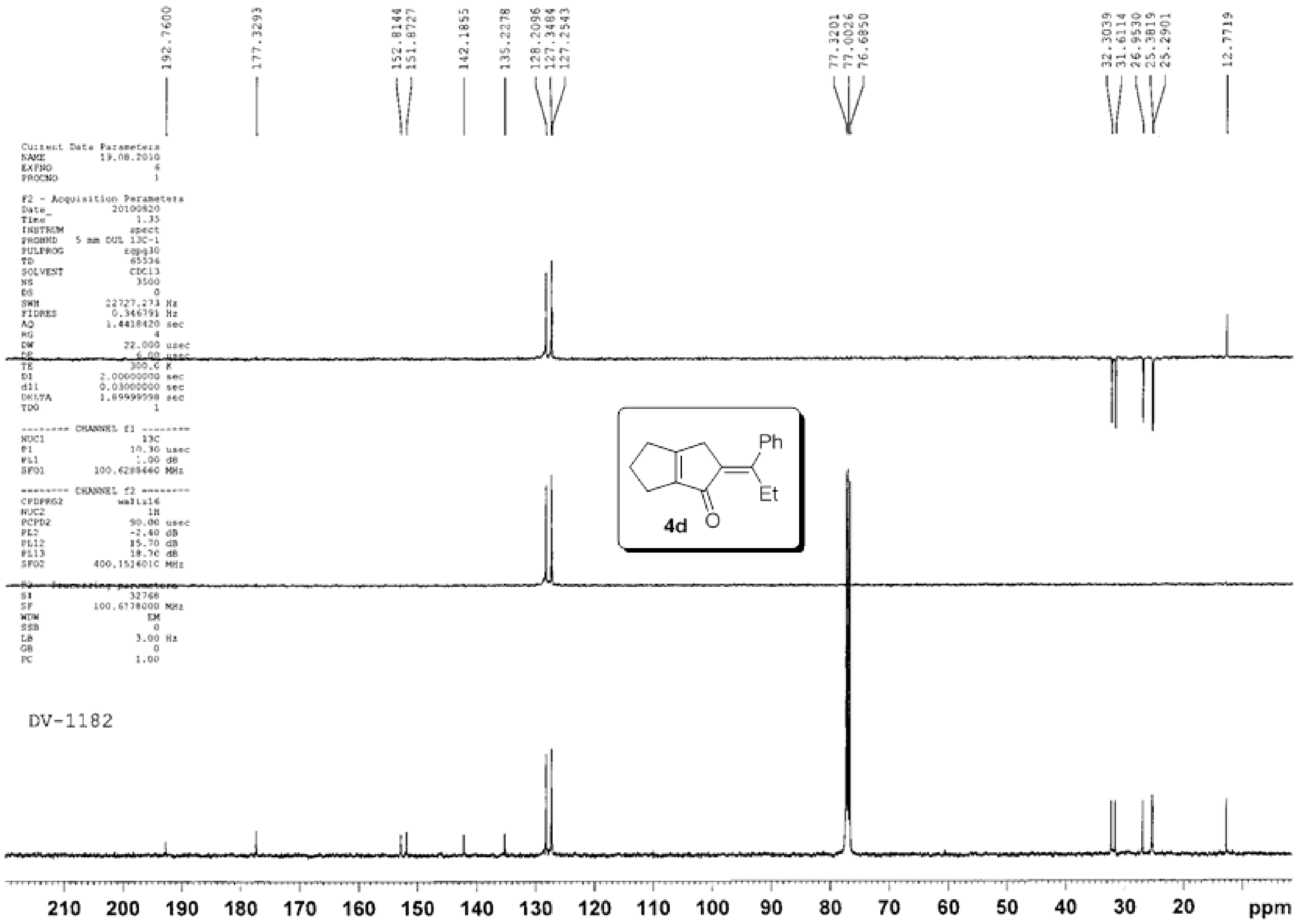

192.7600
177.3293
152.8144
151.8727
142.1855
135.2278
128.2096
127.3484
127.2543
77.3201
77.0026
76.6850
32.3039
31.6114
26.9530
25.3819
25.2901
12.7719
Current Data Parameters
NAME 19.08.2010
EXPNO 6
PROCNO 1
F2 - Acquisition Parameters
Date_ 20100820
Time 1.35
INSTRUM spect
PROBHD 5 mm DUL 13C-1
TD 65536
SOLVENT CDCl3
NS 3500
DS 0
SWH 22727.273 Hz
AQ 1.4418420 sec
RG 4
DW 22.000 usec
TE 300.0 K
D1 2.00000000 sec
d11 0.03000000 sec
TD0 1
CHANNEL f1
NUC1 13C
P1 10.30 usec
SFO1 100.6288440 MHz
CHANNEL f2
NUC2 1H
PCPD2 90.00 usec
PL2 -2.40 dB
SFO2 400.1514010 MHz
SI 32768
SF 100.6178000 MHz
WDW EM
SSB 0
LB 3.00 Hz
GB 0
PC 1.00
Ph
Et
O
4d
DV-1182
210
200
190
180
170
160
150
140
130
120
110
100
90
80
70
60
50
40
30
20
ppm

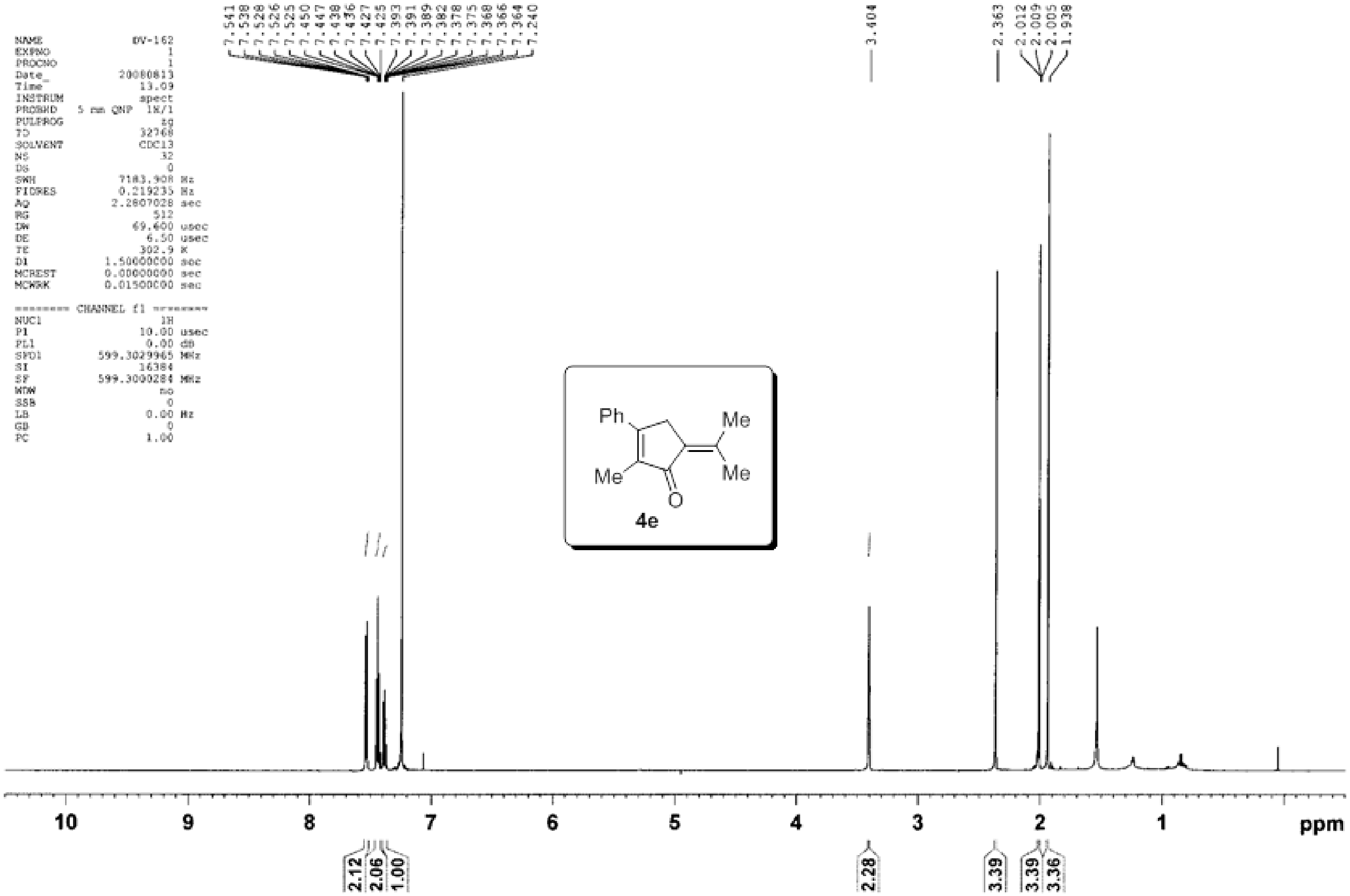

NAME DV-162
EXPNO 1
PROCNO 1
Date_ 20080813
Time 13.09
INSTRUM spect
PROBHD 5 mm QNP 1H/1
PULPROG zg
TD 32768
SOLVENT CDCl3
NS 32
DS 0
SWH 7183.908 Hz
FIDRES 0.219235 Hz
AQ 2.2807028 sec
RG 512
DW 69.600 usec
DE 6.50 usec
TE 302.9 K
D1 1.50000000 sec
MCREST 0.00000000 sec
MCWRK 0.01500000 sec
======== CHANNEL f1 ========
NUC1 1H
P1 10.00 usec
PL1 0.00 dB
SFO1 599.3029965 MHz
SI 16384
SF 599.3000284 MHz
WDW no
SSB 0
LB 0.00 Hz
GB 0
PC 1.00
7.541 7.538 7.528 7.526 7.525 7.450 7.447 7.438 7.436 7.427 7.425 7.393 7.391 7.389 7.382 7.378 7.375 7.368 7.366 7.364 7.240
3.404
2.363 2.012 2.009 2.005 1.938
Ph
Me
Me
Me
O
4e
10 9 8 7 6 5 4 3 2 1 ppm
2.12 2.06 1.00
2.28
3.39 3.39 3.36

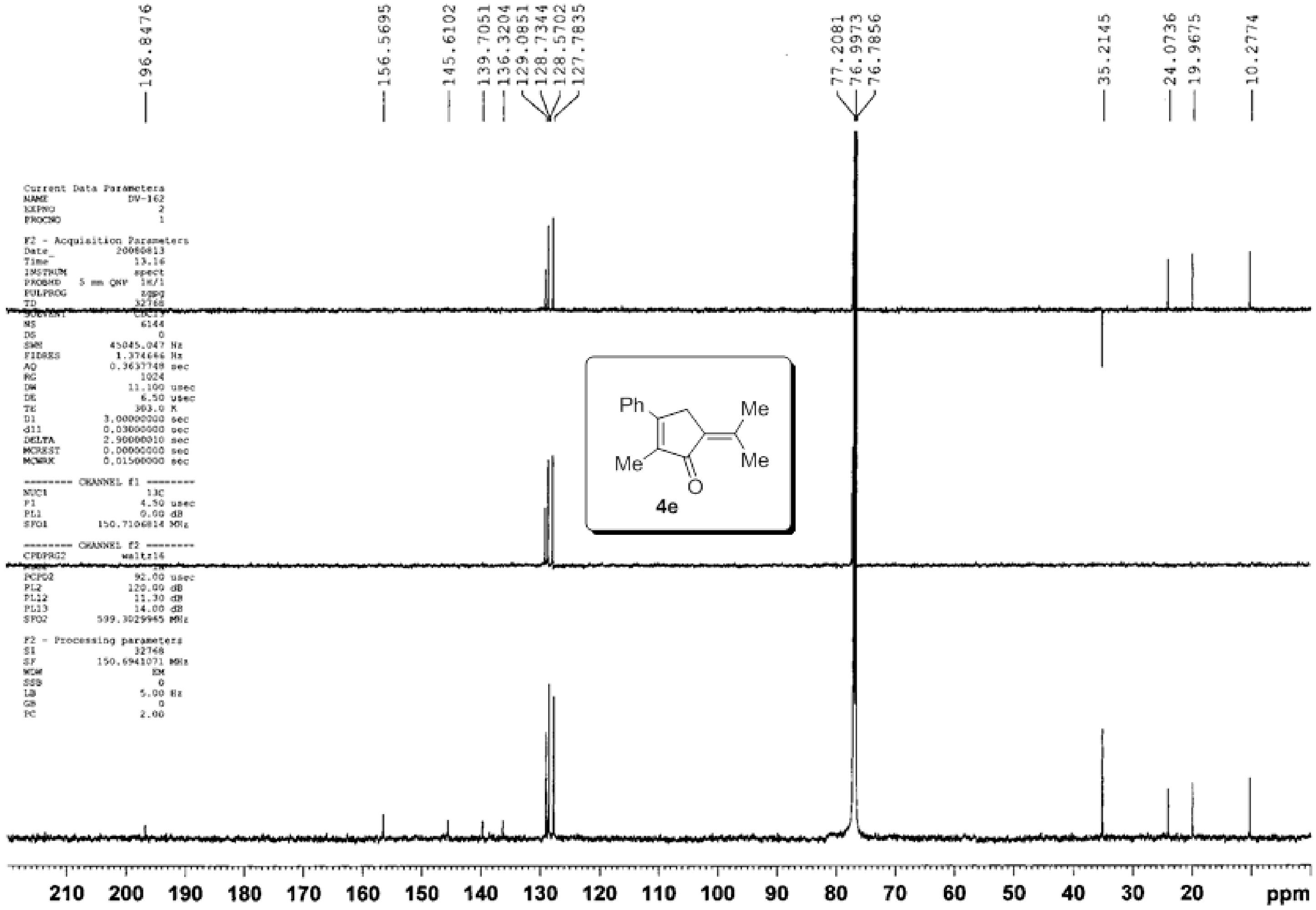
196.8476
156.5695
145.6102
139.7051
136.3204
129.0851
128.7344
128.5702
127.7835
77.2081
76.9973
76.7856
35.2145
24.0736
19.9675
10.2774
Current Data Parameters
NAME DV-162
EXPNO 2
PROCNO 1
F2 - Acquisition Parameters
Date_ 20080813
Time 13.16
INSTRUM spect
PROBHD 5 mm QNP 1H/1
PULPROG zgpg
TD 32768
NS 6144
DS 0
SWH 45045.047 Hz
FIDRES 1.374666 Hz
AQ 0.3637748 sec
RG 1024
DW 11.100 usec
DE 6.50 usec
TE 303.0 K
D1 3.00000000 sec
d11 0.03000000 sec
DELTA 2.90000010 sec
MCREST 0.00000000 sec
MCWRK 0.01500000 sec
======== CHANNEL f1 ========
NUC1 13C
P1 4.50 usec
PL1 0.00 dB
SFO1 150.7106814 MHz
======== CHANNEL f2 ========
CPDPRG2 waltz16
PCPD2 92.00 usec
PL2 120.00 dB
PL12 11.30 dB
PL13 14.00 dB
SFO2 599.3029965 MHz
F2 - Processing parameters
SI 32768
SF 150.6941071 MHz
WDW EM
SSB 0
LB 5.00 Hz
GB 0
PC 2.00
Ph
Me
Me
Me
O
4e
210 200 190 180 170 160 150 140 130 120 110 100 90 80 70 60 50 40 30 20 ppm

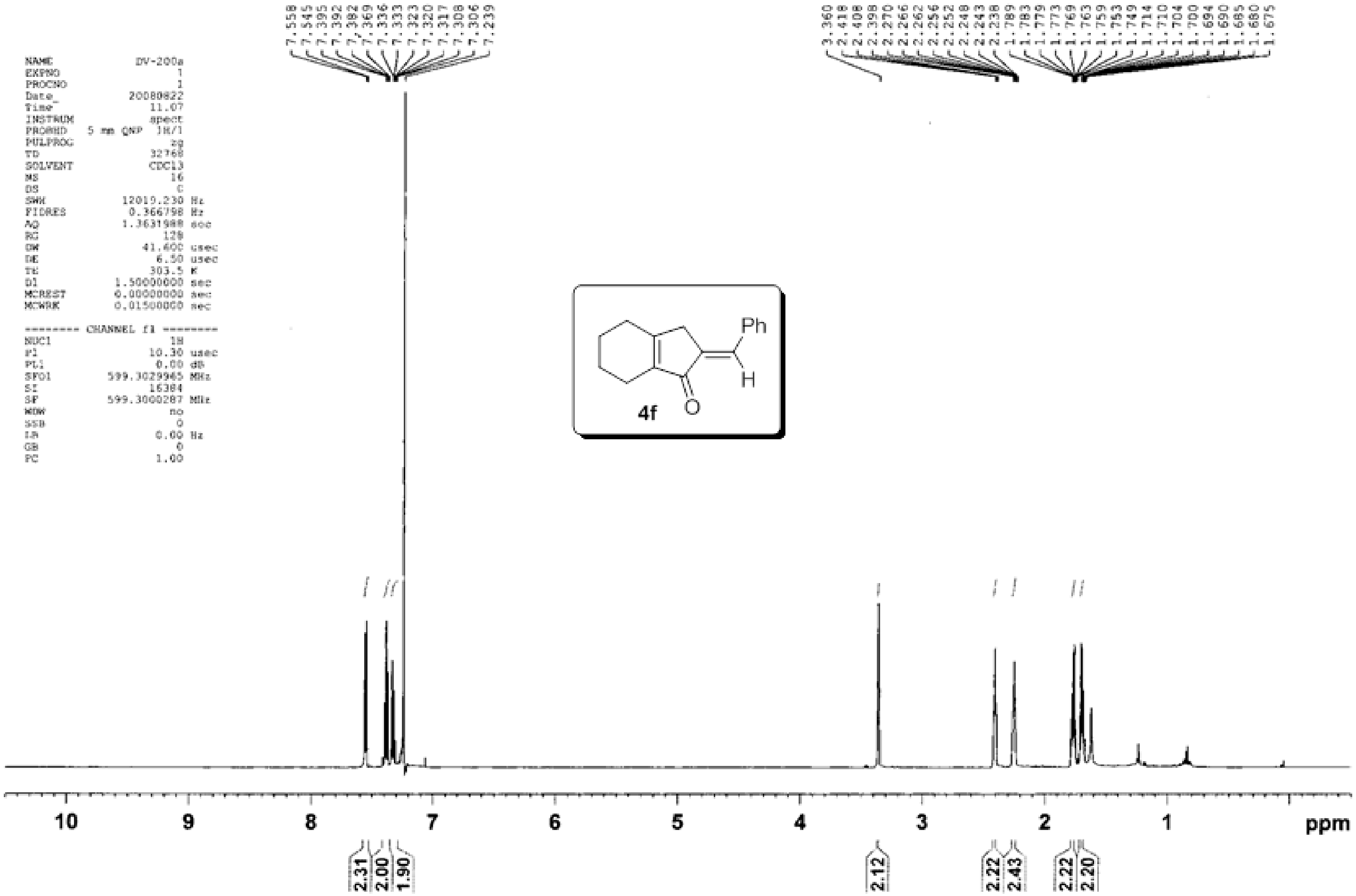
Ph
H
O
4f
10
9
8
7
6
5
4
3
2
1
ppm
2.31
2.00
1.90
2.12
2.22
2.43
2.22
2.20

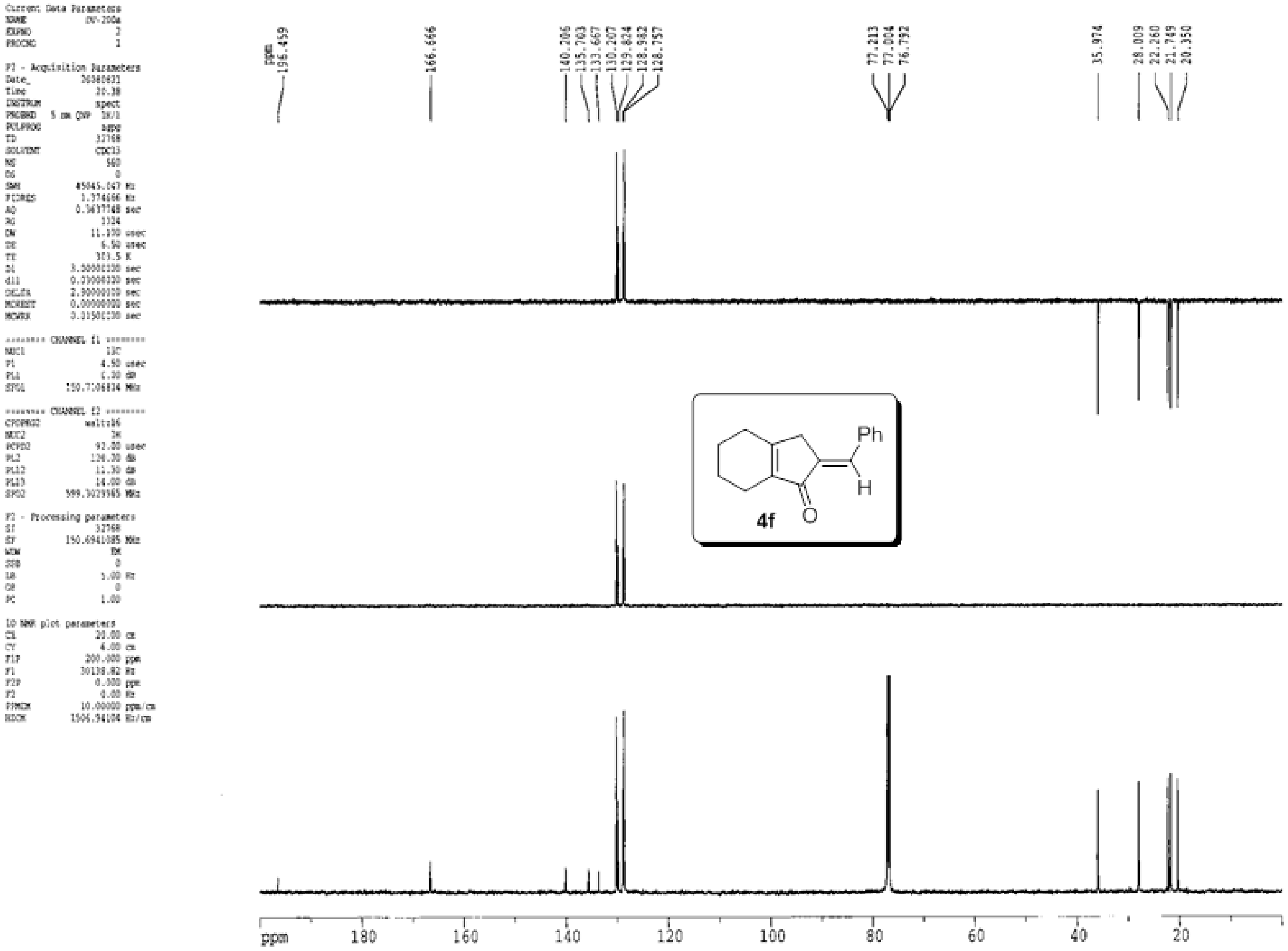

Current Data Parameters
ppm
196.459
166.666
140.206
135.703
133.667
130.207
129.824
128.982
128.757
77.213
77.004
76.792
35.974
28.009
22.260
21.749
20.350
Ph
H
O
4f
ppm
180
160
140
120
100
80
60
40
20

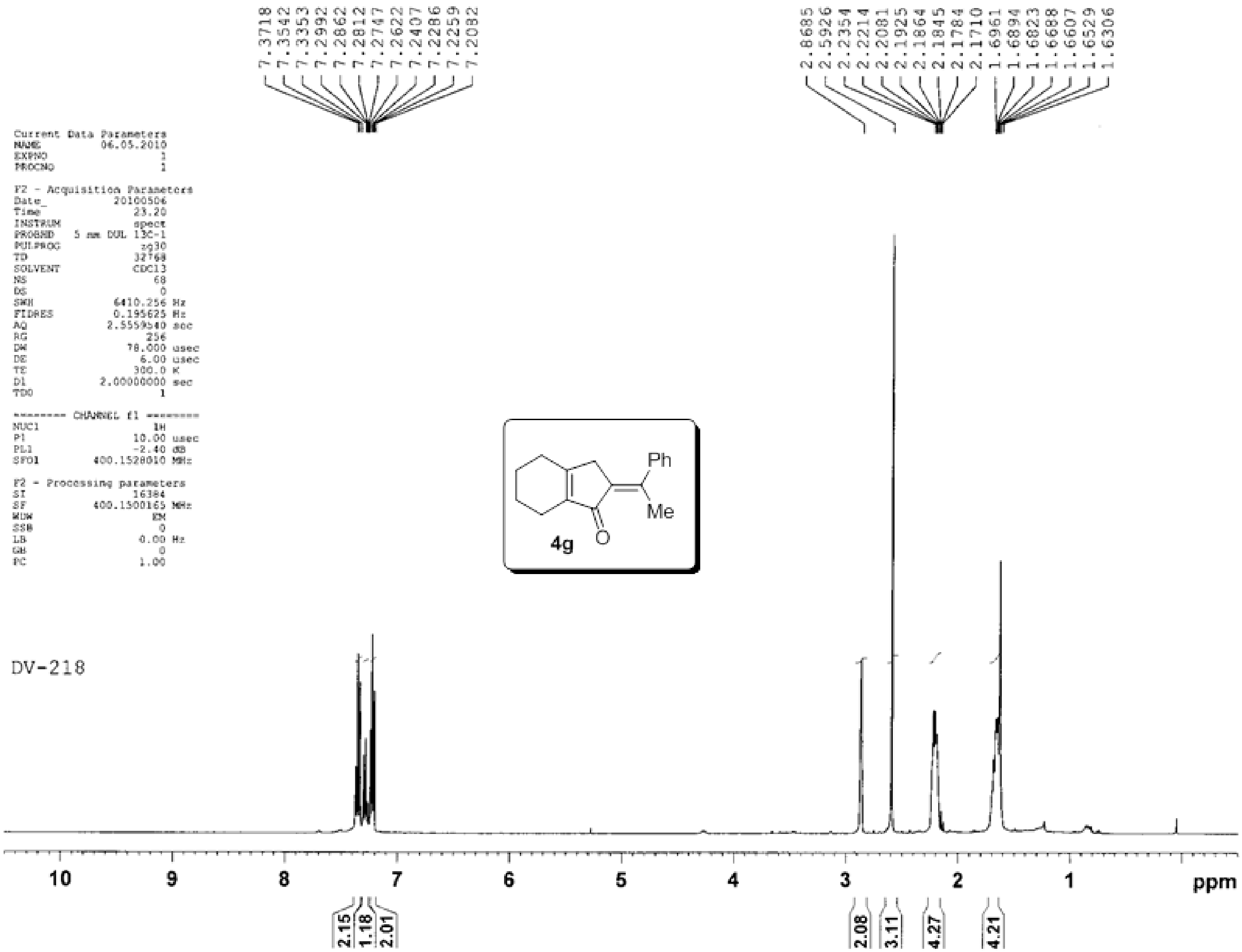

7.3718
7.3542
7.3353
7.2992
7.2862
7.2812
7.2747
7.2622
7.2407
7.2286
7.2259
7.2082
2.8685
2.5926
2.2354
2.2214
2.2081
2.1925
2.1864
2.1845
2.1784
2.1710
1.6961
1.6894
1.6823
1.6688
1.6607
1.6529
1.6306
Current Data Parameters
NAME 06.05.2010
EXPNO 1
PROCNO 1
F2 - Acquisition Parameters
Date_ 20100506
Time 23.20
INSTRUM spect
PROBHD 5 mm DUL 13C-1
PULPROG zg30
TD 32768
SOLVENT CDCl3
NS 68
DS 0
SWH 6410.256 Hz
FIDRES 0.195625 Hz
AQ 2.5559540 sec
RG 256
DW 78.000 usec
DE 6.00 usec
TE 300.0 K
D1 2.00000000 sec
TD0 1
======== CHANNEL f1 ========
NUC1 1H
P1 10.00 usec
PL1 -2.40 dB
SFO1 400.1528010 MHz
F2 - Processing parameters
SI 16384
SF 400.1500165 MHz
WDW EM
SSB 0
LB 0.00 Hz
GB 0
PC 1.00
DV-218
Ph
Me
O
4g
10
9
8
7
6
5
4
3
2
1
ppm
2.15
1.18
2.01
2.08
3.11
4.27
4.21

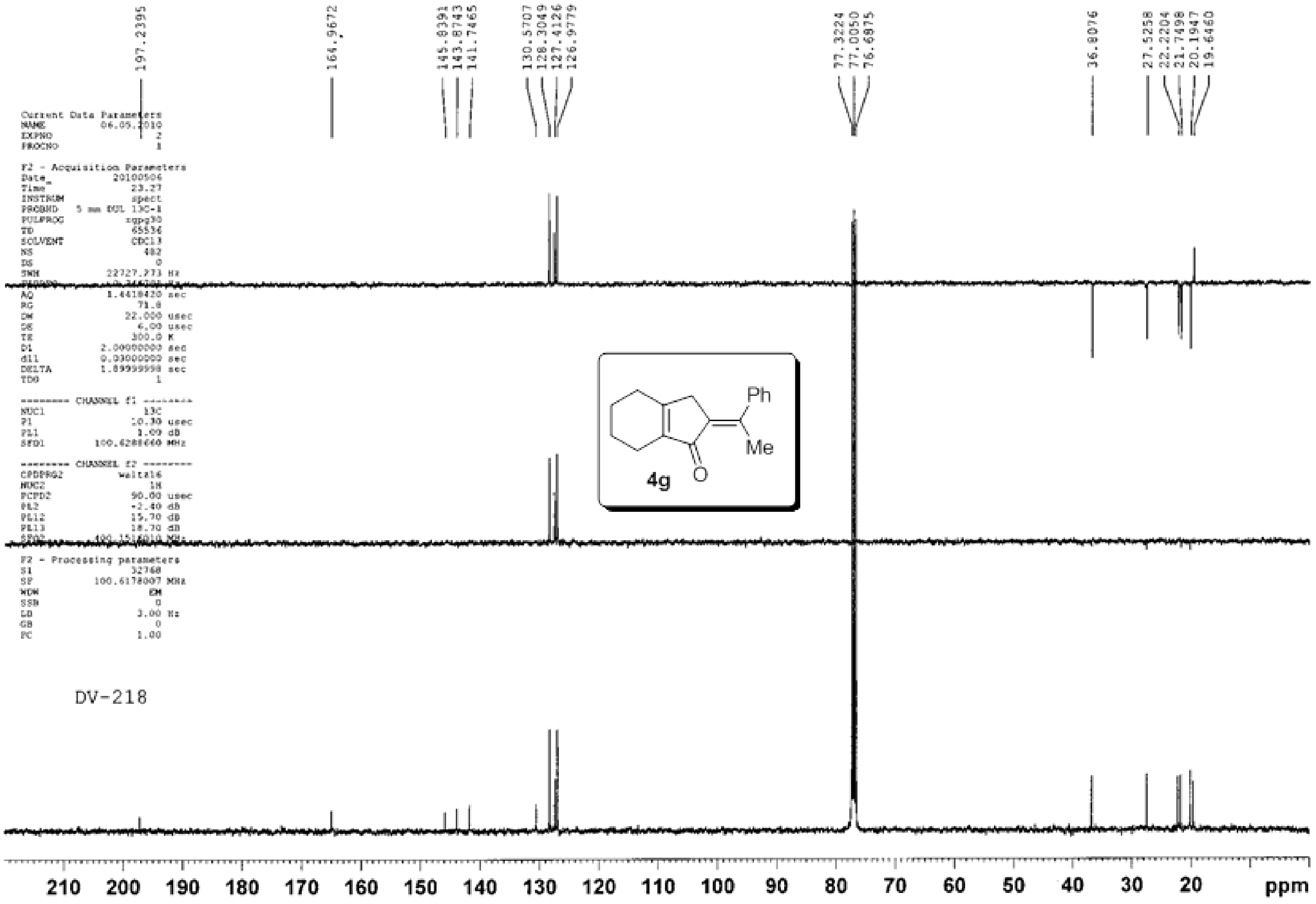
197.2395
164.9672
145.0391
143.8743
141.7465
130.5707
128.3049
127.4126
126.9779
77.3224
77.0050
76.6875
36.8076
27.5258
22.2204
21.7498
20.1947
19.6460
Ph
Me
O
4g
DV-218
210 200 190 180 170 160 150 140 130 120 110 100 90 80 70 60 50 40 30 20 ppm

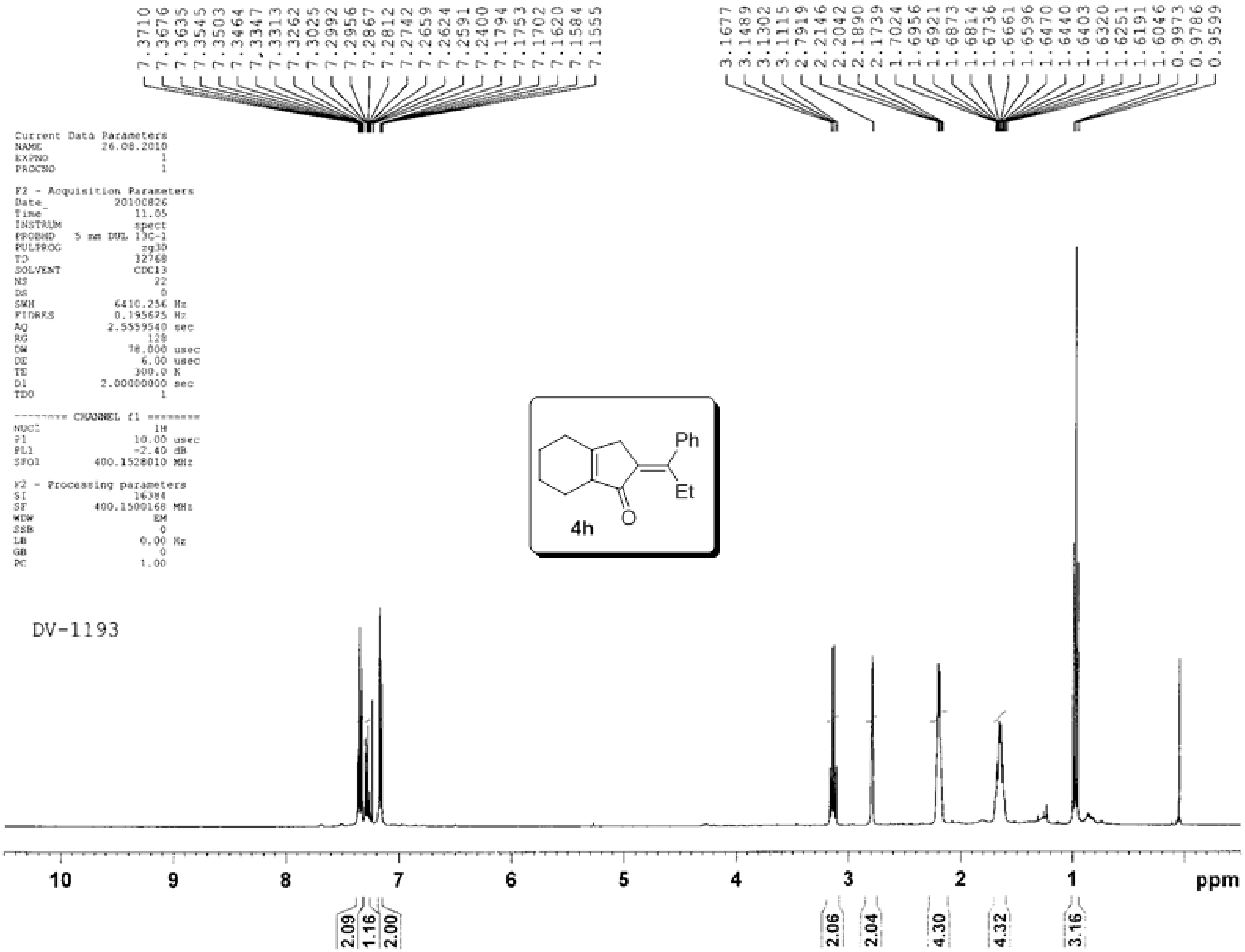
DV-1193
Ph
Et
O
4h
ppm

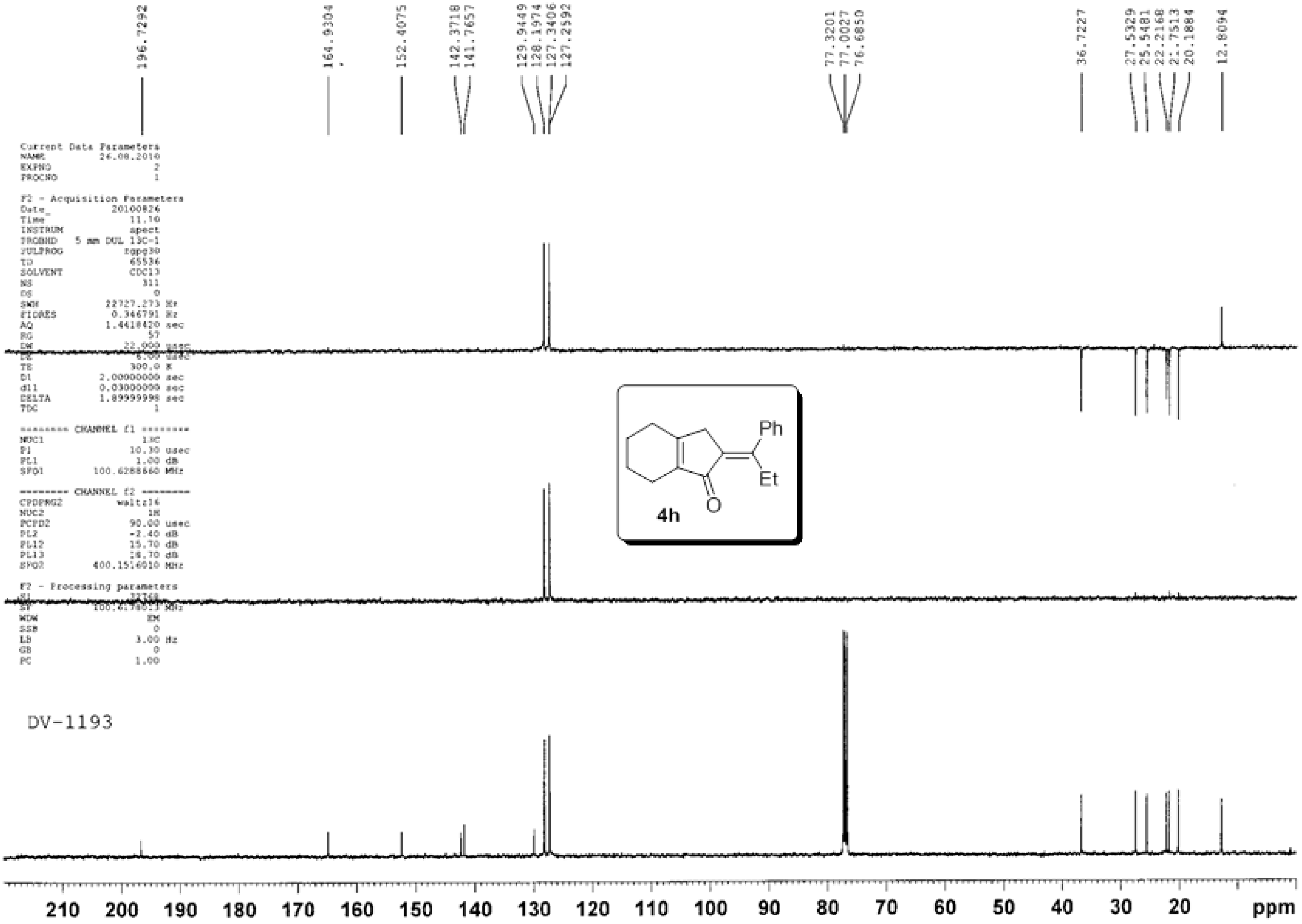
196.7292
164.9304
152.4075
142.3718
141.7657
129.9449
128.1974
127.3406
127.2592
77.3201
77.0027
76.6850
36.7227
27.5329
25.5481
22.2168
21.7513
20.1884
12.8094
Ph
Et
O
4h
DV-1193
ppm

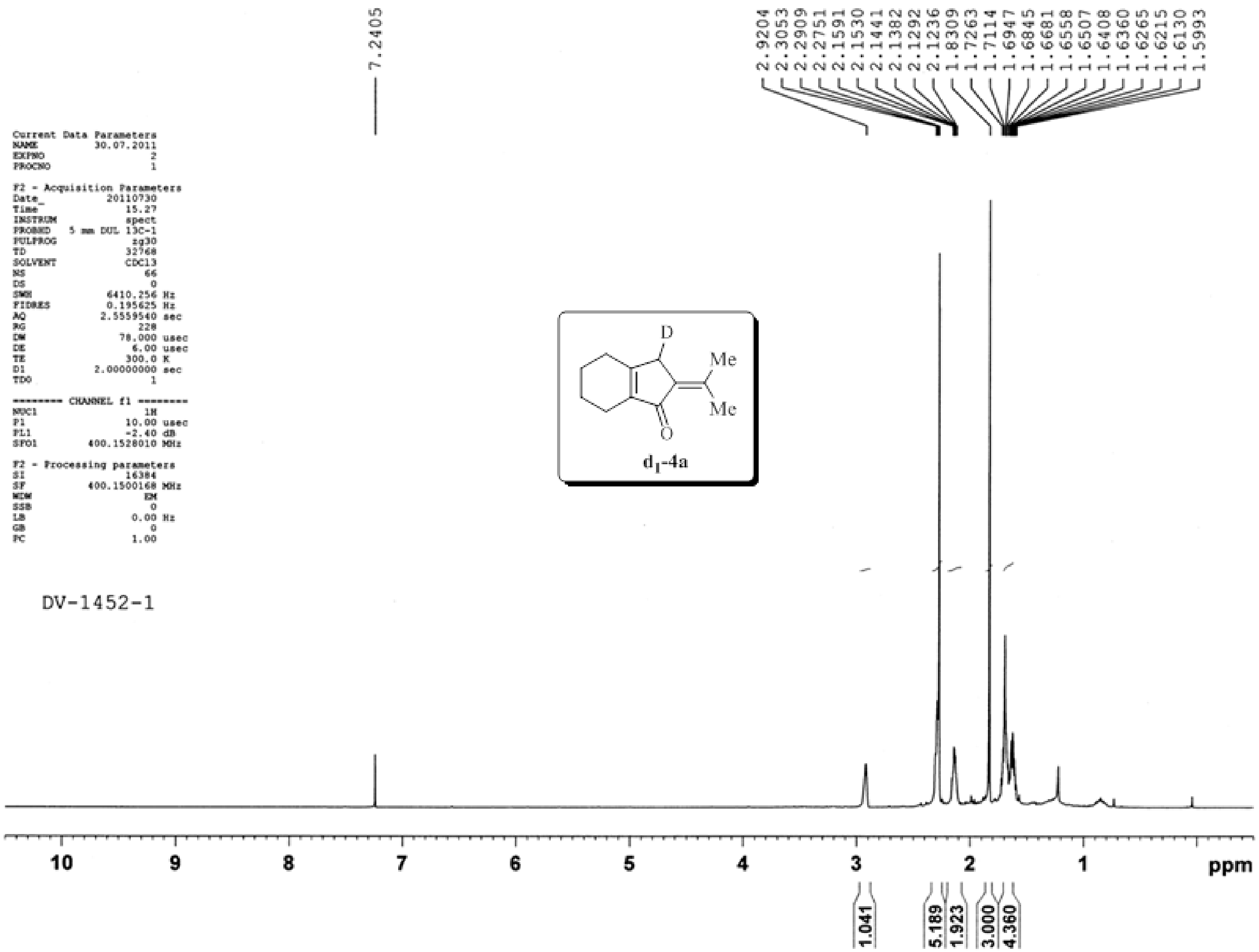
Current Data Parameters
NAME 30.07.2011
EXPNO 2
PROCNO 1
F2 - Acquisition Parameters
Date_ 20110730
Time 15.27
INSTRUM spect
PROBHD 5 mm DUL 13C-1
PULPROG zg30
TD 32768
SOLVENT CDCl3
NS 66
DS 0
SWH 6410.256 Hz
FIDRES 0.195625 Hz
AQ 2.5559540 sec
RG 228
DW 78.000 usec
DE 6.00 usec
TE 300.0 K
D1 2.00000000 sec
TD0 1
======== CHANNEL f1 ========
NUC1 1H
P1 10.00 usec
PL1 -2.40 dB
SFO1 400.1528010 MHz
F2 - Processing parameters
SI 16384
SF 400.1500168 MHz
WDW EM
SSB 0
LB 0.00 Hz
GB 0
PC 1.00
DV-1452-1
7.2405
2.9204
2.3053
2.2909
2.2751
2.1591
2.1530
2.1441
2.1382
2.1292
2.1236
1.8309
1.7263
1.7114
1.6947
1.6845
1.6681
1.6558
1.6507
1.6408
1.6360
1.6265
1.6215
1.6130
1.5993
D
Me
Me
O
d1-4a
10
9
8
7
6
5
4
3
2
1
ppm
1.041
5.189
1.923
3.000
4.360

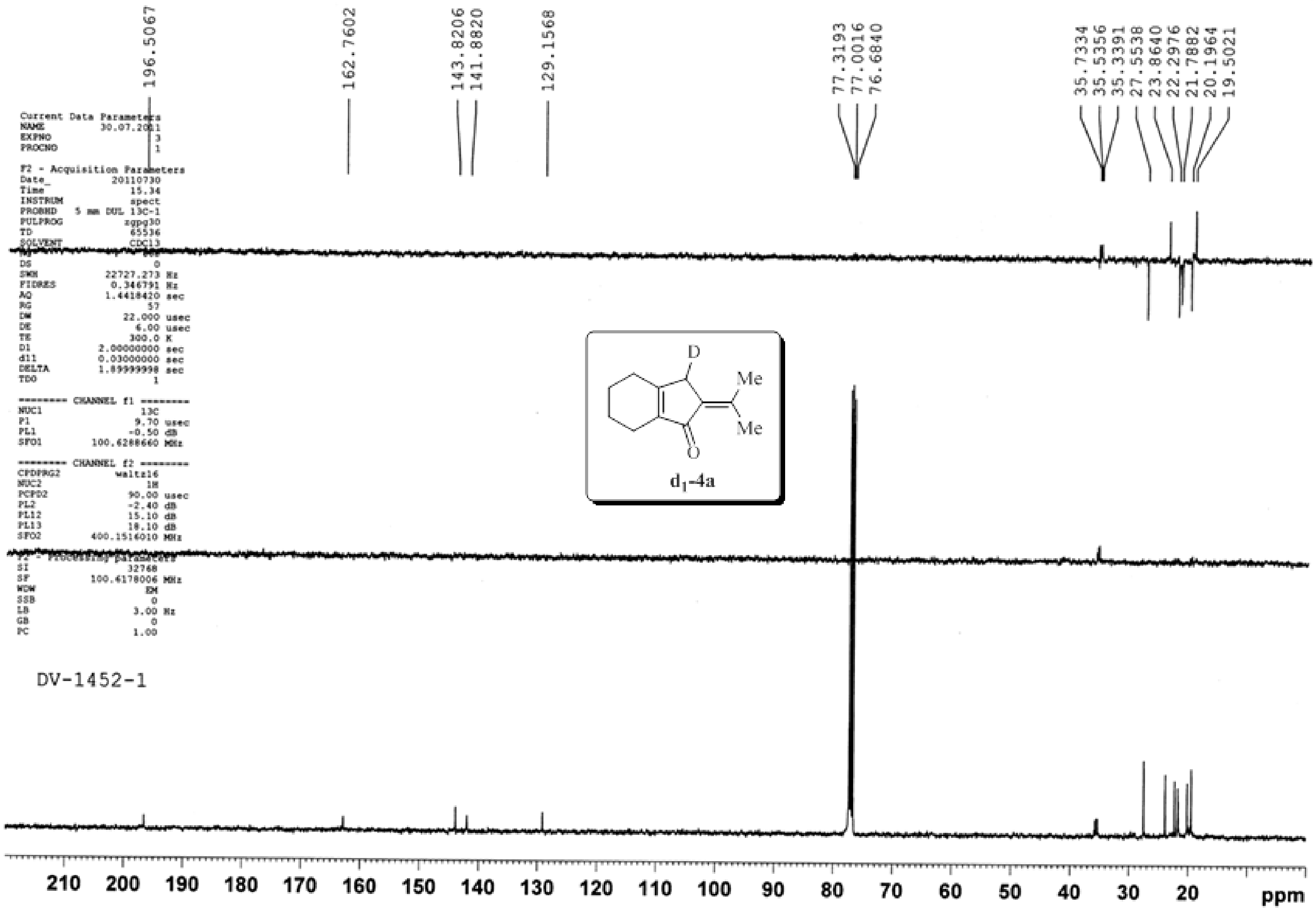
196.5067
162.7602
143.8206
141.8820
129.1568
77.3193
77.0016
76.6840
35.7334
35.5356
35.3391
27.5538
23.8640
22.2976
21.7882
20.1964
19.5021
Current Data Parameters
NAME 30.07.2011
EXPNO 3
PROCNO 1
F2 - Acquisition Parameters
Date_ 20110730
Time 15.34
INSTRUM spect
PROBHD 5 mm DUL 13C-1
PULPROG zgpg30
TD 65536
SOLVENT CDCl3
DS 0
SWH 22727.273 Hz
FIDRES 0.346791 Hz
AQ 1.4418420 sec
RG 57
DW 22.000 usec
DE 6.00 usec
TE 300.0 K
D1 2.00000000 sec
d11 0.03000000 sec
DELTA 1.89999998 sec
TD0 1
======== CHANNEL f1 ========
NUC1 13C
P1 9.70 usec
PL1 -0.50 dB
SFO1 100.6288660 MHz
======== CHANNEL f2 ========
CPDPRG2 waltz16
NUC2 1H
PCPD2 90.00 usec
PL2 -2.40 dB
PL12 15.10 dB
PL13 18.10 dB
SFO2 400.1516010 MHz
F2 - Processing parameters
SI 32768
SF 100.6178006 MHz
WDW EM
SSB 0
LB 3.00 Hz
GB 0
PC 1.00
DV-1452-1
D
Me
Me
O
d1-4a
210 200 190 180 170 160 150 140 130 120 110 100 90 80 70 60 50 40 30 20 ppm

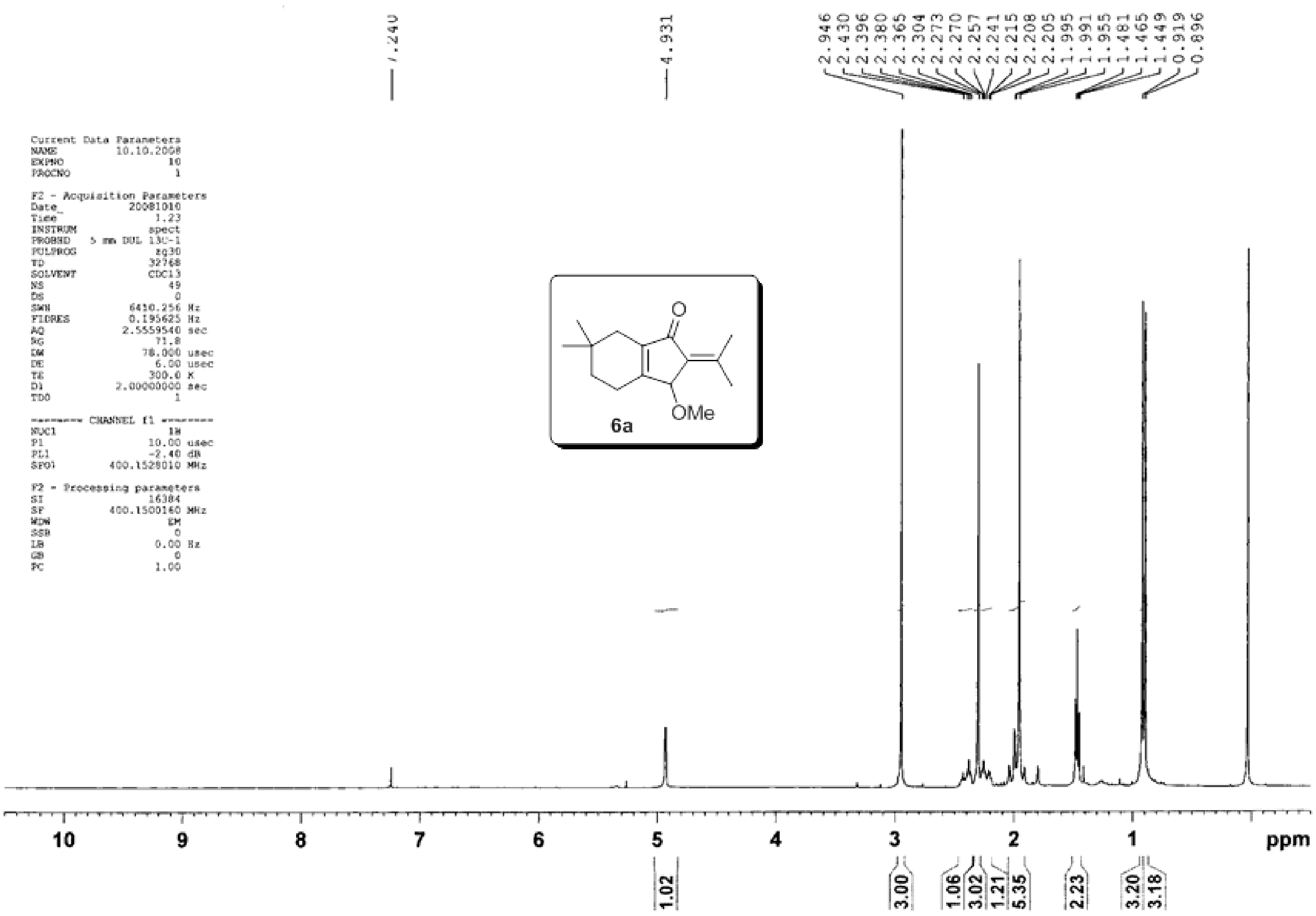

7.240
4.931
2.946
2.430
2.396
2.380
2.365
2.304
2.273
2.270
2.257
2.241
2.215
2.208
2.205
1.995
1.991
1.955
1.481
1.465
1.449
0.919
0.896
Current Data Parameters
NAME 10.10.2008
EXPNO 10
PROCNO 1
F2 - Acquisition Parameters
Date_ 20081010
Time 1.23
INSTRUM spect
PROBHD 5 mm DUL 13C-1
PULPROG zg30
TD 32768
SOLVENT CDCl3
NS 49
DS 0
SWH 6410.256 Hz
FIDRES 0.195625 Hz
AQ 2.5559540 sec
RG 71.8
DW 78.000 usec
DE 6.00 usec
TE 300.0 K
D1 2.00000000 sec
TD0 1
======== CHANNEL f1 ========
NUC1 1H
P1 10.00 usec
PL1 -2.40 dB
SFO1 400.1528010 MHz
F2 - Processing parameters
SI 16384
SF 400.1500160 MHz
WDW EM
SSB 0
LB 0.00 Hz
GB 0
PC 1.00
O
OMe
6a
10
9
8
7
6
5
4
3
2
1
ppm
1.02
3.00
1.06
3.02
1.21
5.35
2.23
3.20
3.18

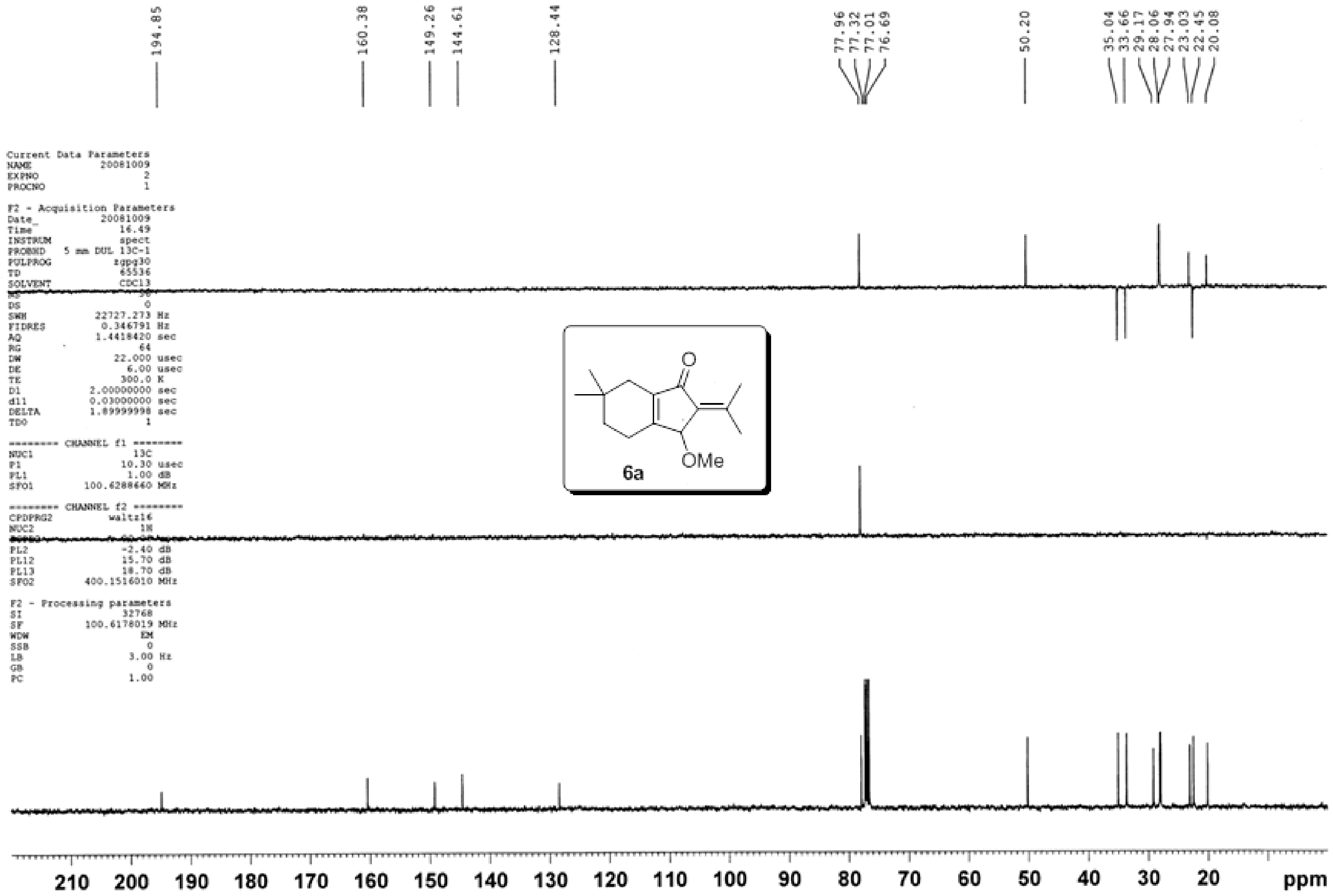
194.85
160.38
149.26
144.61
128.44
77.96
77.32
77.01
76.69
50.20
35.04
33.66
29.17
28.06
27.94
23.03
22.45
20.08
Current Data Parameters
NAME 20081009
EXPNO 2
PROCNO 1
F2 - Acquisition Parameters
Date_ 20081009
Time 16.49
INSTRUM spect
PROBHD 5 mm DUL 13C-1
PULPROG zgpg30
TD 65536
SOLVENT CDCl3
DS 0
SWH 22727.273 Hz
FIDRES 0.346791 Hz
AQ 1.4418420 sec
RG 64
DW 22.000 usec
DE 6.00 usec
TE 300.0 K
D1 2.00000000 sec
d11 0.03000000 sec
DELTA 1.89999998 sec
TD0 1
======== CHANNEL f1 ========
NUC1 13C
P1 10.30 usec
PL1 1.00 dB
SFO1 100.6288640 MHz
======== CHANNEL f2 ========
CPDPRG2 waltz16
NUC2 1H
PL2 -2.40 dB
PL12 15.70 dB
PL13 18.70 dB
SFO2 400.1516010 MHz
F2 - Processing parameters
SI 32768
SF 100.6178019 MHz
WDW EM
SSB 0
LB 3.00 Hz
GB 0
PC 1.00
O
OMe
6a
210 200 190 180 170 160 150 140 130 120 110 100 90 80 70 60 50 40 30 20 ppm

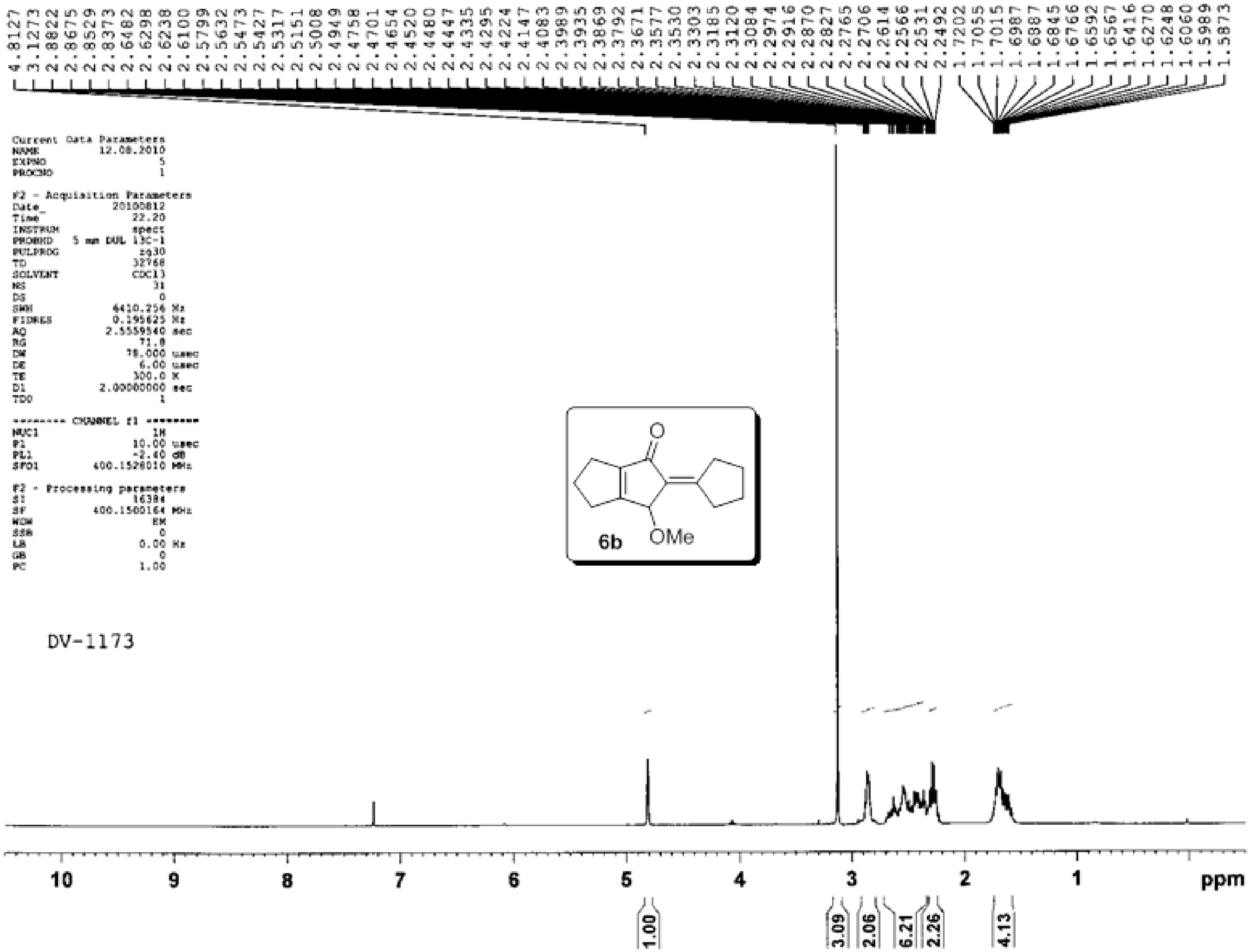

4.8127
3.1273
2.8822
2.8675
2.8529
2.8373
2.6482
2.6298
2.6238
2.6100
2.5799
2.5632
2.5473
2.5427
2.5317
2.5151
2.5008
2.4949
2.4758
2.4701
2.4654
2.4520
2.4480
2.4447
2.4335
2.4295
2.4224
2.4147
2.4083
2.3989
2.3935
2.3869
2.3792
2.3671
2.3577
2.3530
2.3303
2.3185
2.3120
2.3084
2.2974
2.2916
2.2870
2.2827
2.2765
2.2706
2.2614
2.2566
2.2531
2.2492
1.7202
1.7055
1.7015
1.6987
1.6887
1.6845
1.6766
1.6592
1.6567
1.6416
1.6270
1.6248
1.6060
1.5989
1.5873
Current Data Parameters
NAME 12.08.2010
EXPNO 5
PROCNO 1
F2 - Acquisition Parameters
Date_ 20100812
Time 22.20
INSTRUM spect
PROBHD 5 mm DUL 13C-1
PULPROG zg30
TD 32768
SOLVENT CDCl3
NS 31
DS 0
SWH 6410.256 Hz
FIDRES 0.195625 Hz
AQ 2.5559540 sec
RG 71.8
DW 78.000 usec
DE 6.00 usec
TE 300.0 K
D1 2.00000000 sec
TD0 1
======== CHANNEL f1 ========
NUC1 1H
P1 10.00 usec
PL1 -2.40 dB
SFO1 400.1528010 MHz
F2 - Processing parameters
SI 16384
SF 400.1500164 MHz
WDW EM
SSB 0
LB 0.00 Hz
GB 0
PC 1.00
DV-1173
O
6b
OMe
10
9
8
7
6
5
4
3
2
1
ppm
1.00
3.09
2.06
6.21
2.26
4.13

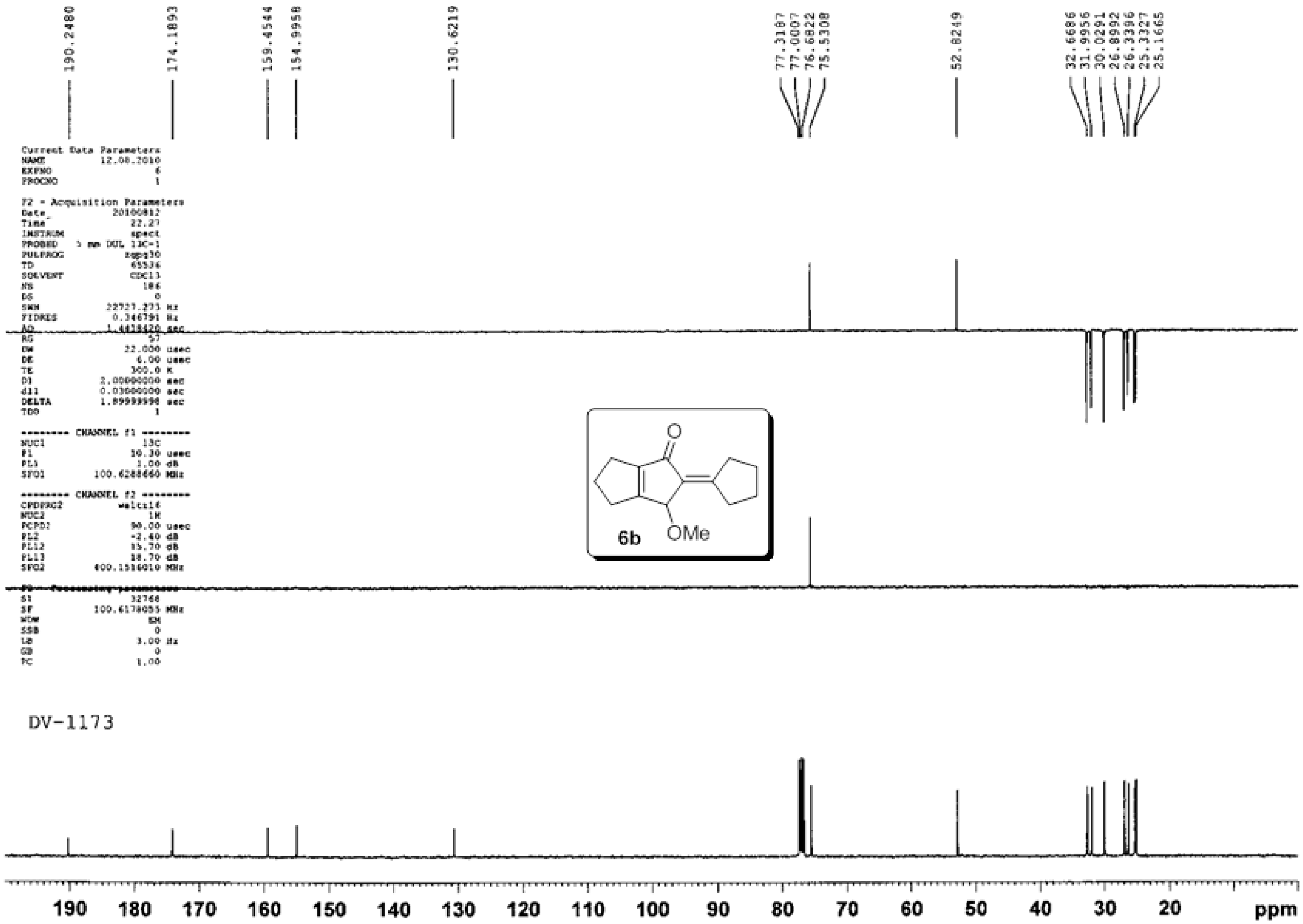
190.2480
174.1893
159.4544
154.9958
130.6219
77.3187
77.0007
76.6822
75.5308
52.8249
32.6686
31.9956
30.0291
26.8992
26.3396
25.3327
25.1665
6b
OMe
DV-1173
190 180 170 160 150 140 130 120 110 100 90 80 70 60 50 40 30 20 ppm

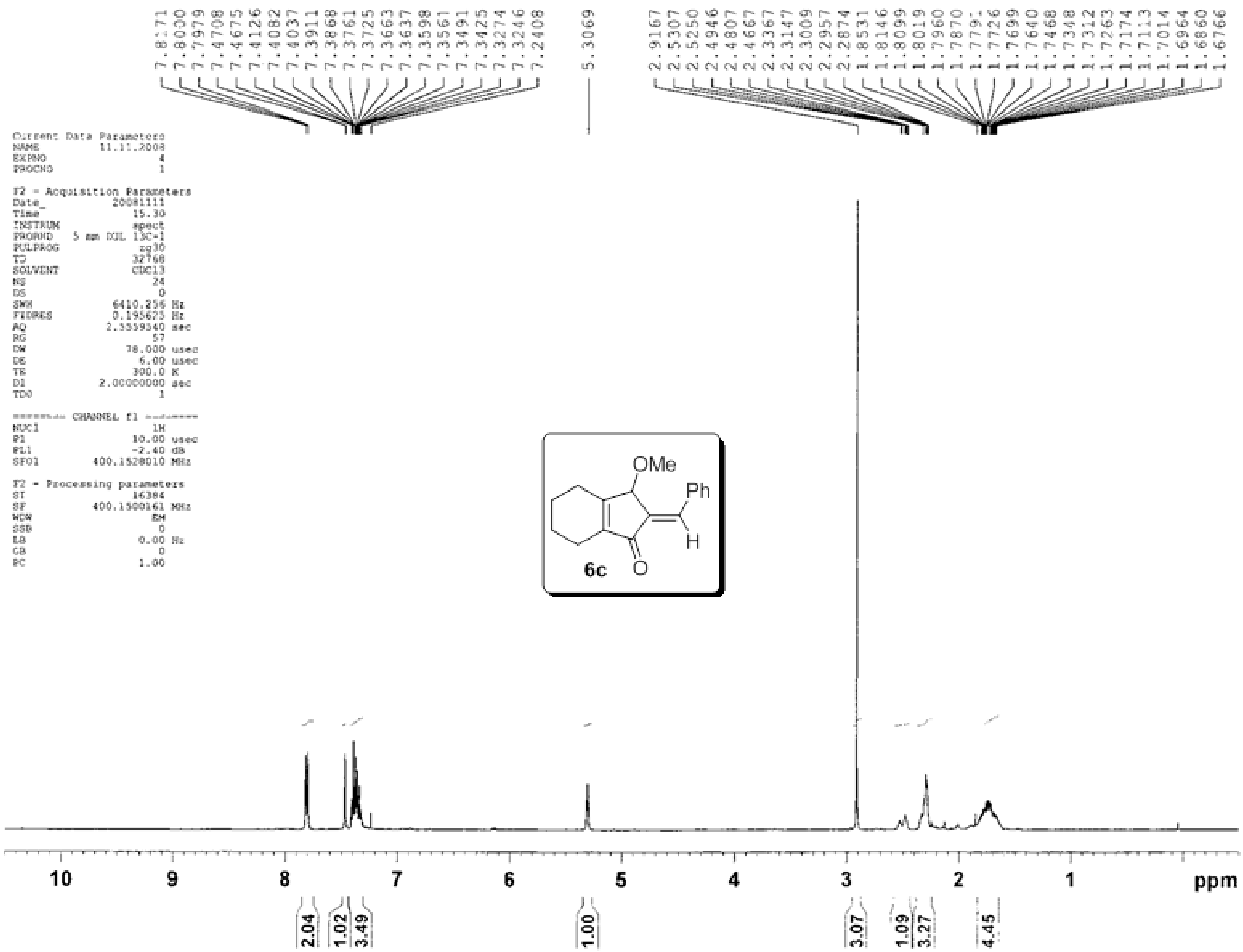
OMe
Ph
H
O
6c
ppm

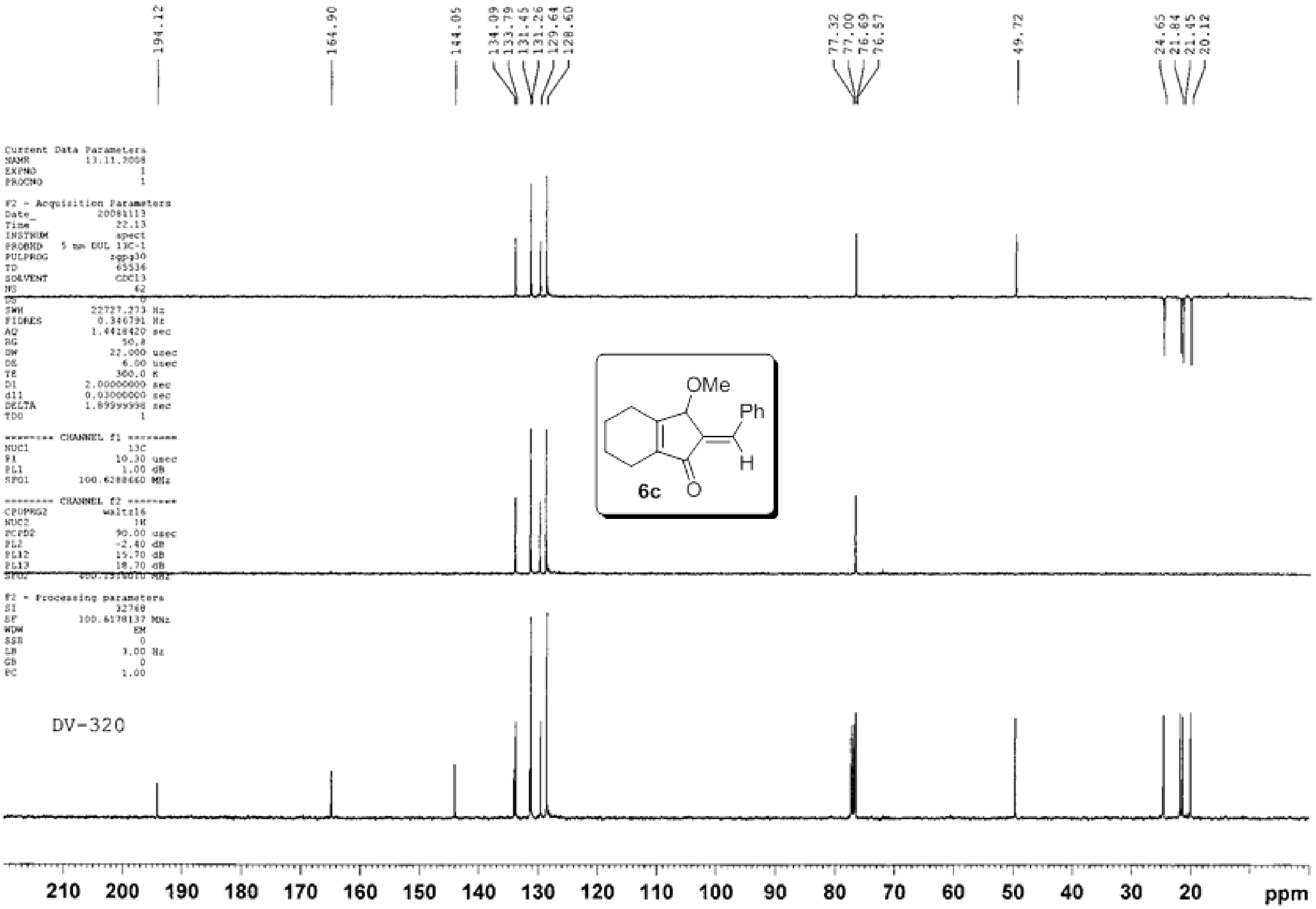

194.12
164.90
144.05
134.09
133.79
131.45
131.26
129.64
128.60
77.32
77.00
76.69
76.57
49.72
24.65
21.84
21.45
20.12
Current Data Parameters
NAME 13.11.2008
EXPNO 1
PROCNO 1
F2 - Acquisition Parameters
Date_ 20081113
Time 22.13
INSTRUM spect
PROBHD 5 mm DUL 13C-1
PULPROG zgpg30
TD 65536
SOLVENT CDCl3
NS 62
SWH 22727.273 Hz
FIDRES 0.346791 Hz
AQ 1.4418420 sec
RG 50.8
DW 22.000 usec
DE 6.00 usec
TE 300.0 K
D1 2.00000000 sec
d11 0.03000000 sec
DELTA 1.89999998 sec
TD0 1
======== CHANNEL f1 ========
NUC1 13C
P1 10.30 usec
PL1 1.00 dB
SFO1 100.6288660 MHz
======== CHANNEL f2 ========
CPDPRG2 waltz16
NUC2 1H
PCPD2 90.00 usec
PL2 -2.40 dB
PL12 15.70 dB
PL13 18.70 dB
F2 - Processing parameters
SI 32768
SF 100.6178137 MHz
WDW EM
SSB 0
LB 3.00 Hz
GB 0
PC 1.00
DV-320
OMe
Ph
H
O
6c
210 200 190 180 170 160 150 140 130 120 110 100 90 80 70 60 50 40 30 20 ppm

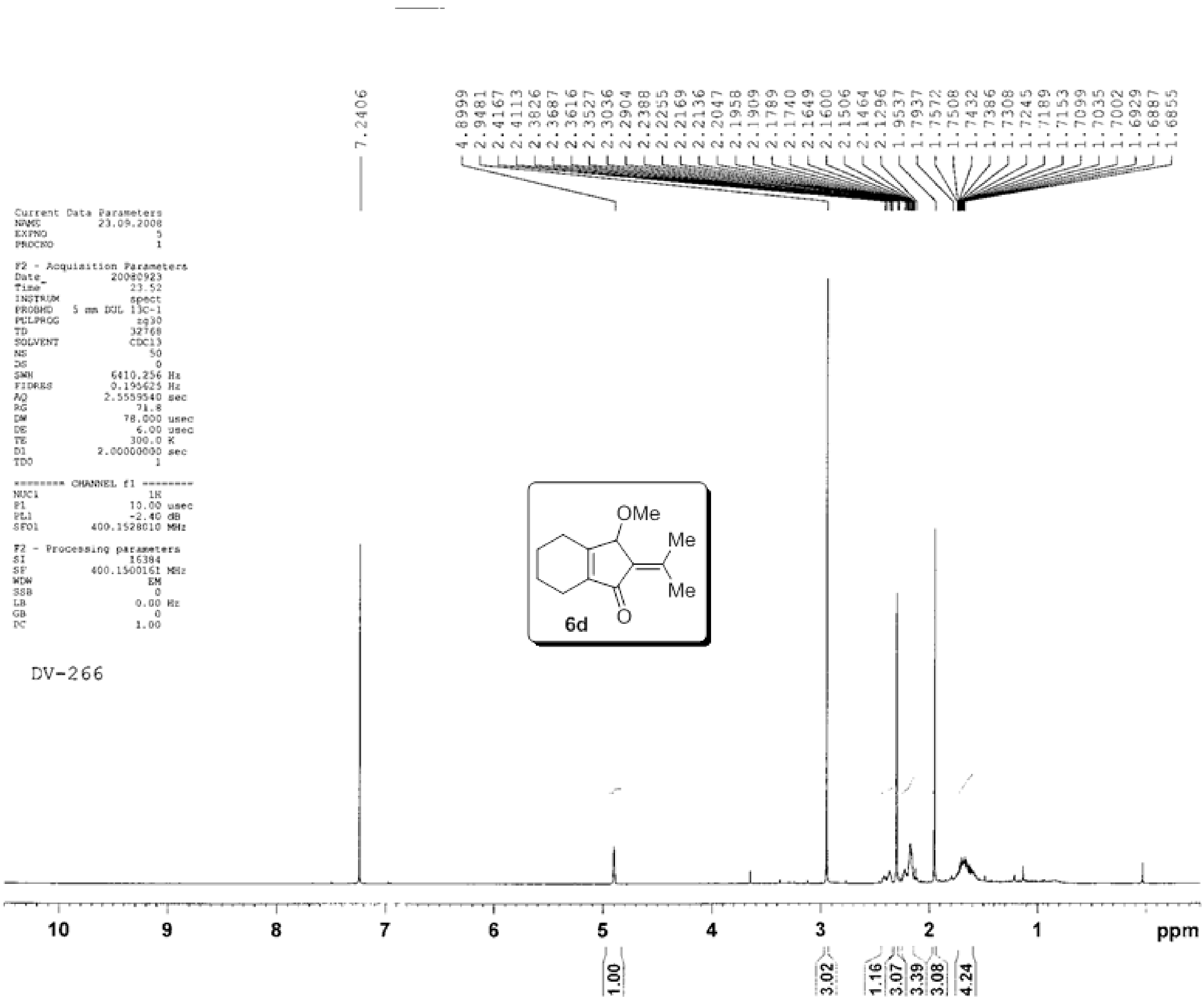
Current Data Parameters
NAME 23.09.2008
EXPNO 5
PROCNO 1
F2 - Acquisition Parameters
Date_ 20080923
Time 23.52
INSTRUM spect
PROBHD 5 mm DUL 13C-1
PULPROG zg30
TD 32768
SOLVENT CDCl3
NS 50
DS 0
SWH 6410.256 Hz
FIDRES 0.195625 Hz
AQ 2.5559540 sec
RG 71.8
DW 78.000 usec
DE 6.00 usec
TE 300.0 K
D1 2.00000000 sec
TD0 1
======== CHANNEL f1 ========
NUC1 1H
P1 10.00 usec
PL1 -2.40 dB
SFO1 400.1528010 MHz
F2 - Processing parameters
SI 16384
SF 400.1500161 MHz
WDW EM
SSB 0
LB 0.00 Hz
GB 0
PC 1.00
DV-266
7.2406
4.8999 2.9481 2.4167 2.4113 2.3826 2.3687 2.3616 2.3527 2.3036 2.2904 2.2388 2.2255 2.2169 2.2136 2.2047 2.1958 2.1909 2.1789 2.1740 2.1649 2.1600 2.1506 2.1464 2.1296 1.9537 1.7937 1.7572 1.7508 1.7432 1.7386 1.7308 1.7245 1.7189 1.7153 1.7099 1.7035 1.7002 1.6929 1.6887 1.6855
OMe
Me
Me
O
6d
10 9 8 7 6 5 4 3 2 1 ppm
1.00 3.02 1.16 3.07 3.39 3.08 4.24

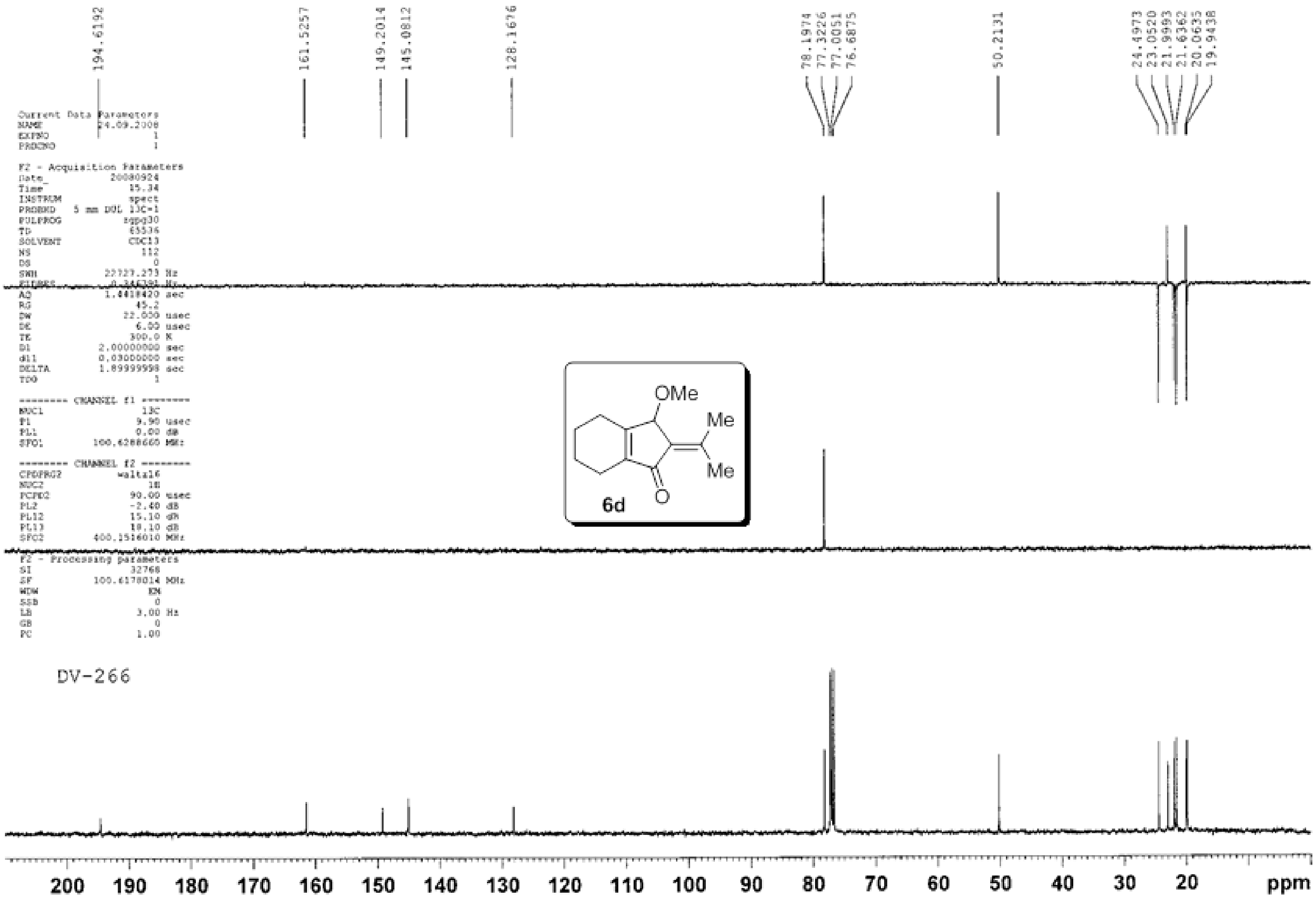
194.6192
161.5257
149.2014
145.0812
128.1676
78.1974
77.3226
77.0051
76.6875
50.2131
24.4973
23.0520
21.9993
21.6362
20.0635
19.9438
DV-266
OMe
Me
Me
O
6d
200 190 180 170 160 150 140 130 120 110 100 90 80 70 60 50 40 30 20 ppm

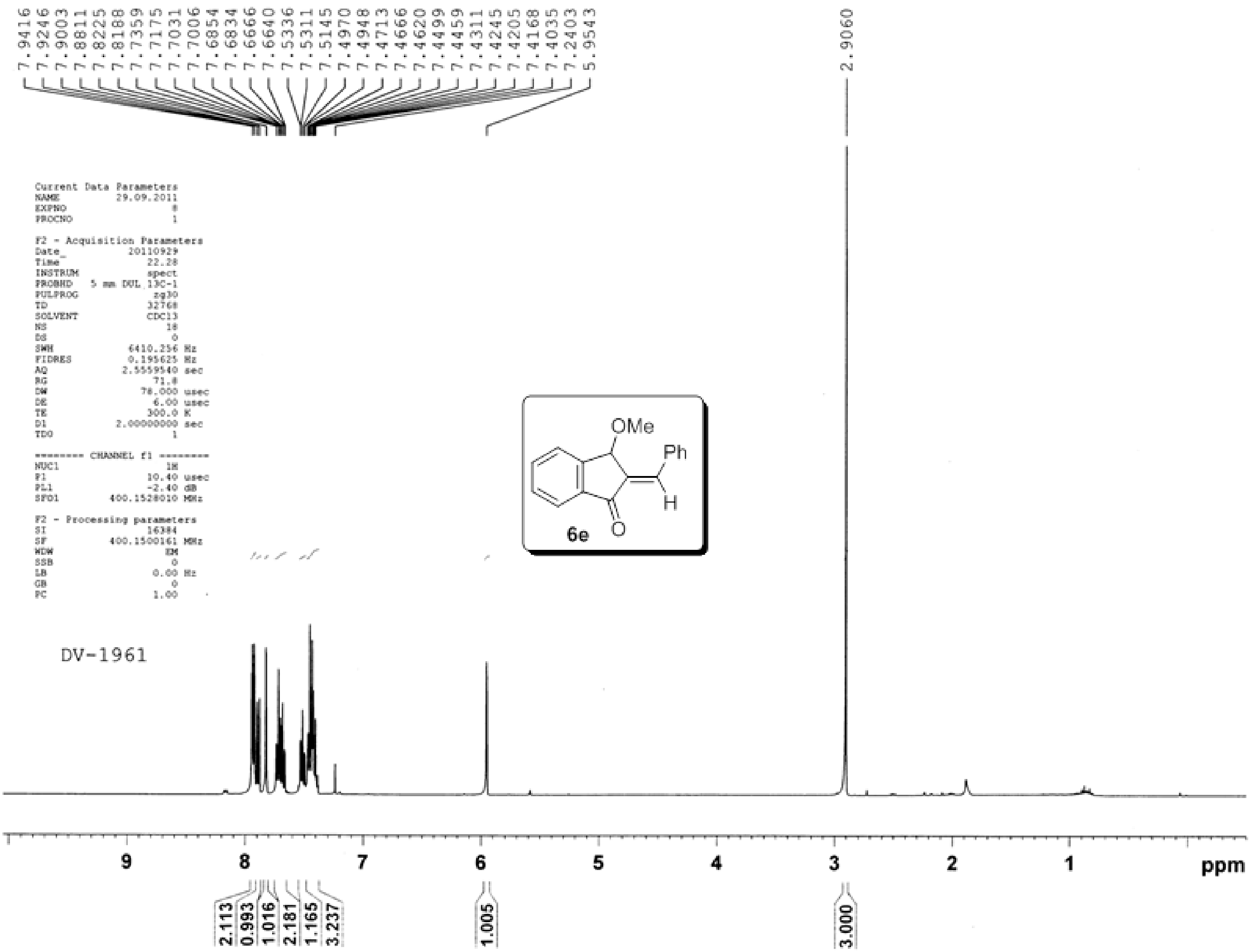
7.9416
7.9246
7.9003
7.8811
7.8225
7.8188
7.7359
7.7175
7.7031
7.7006
7.6854
7.6834
7.6666
7.6640
7.5336
7.5311
7.5145
7.4970
7.4948
7.4713
7.4666
7.4620
7.4499
7.4459
7.4311
7.4245
7.4205
7.4168
7.4035
7.2403
5.9543
2.9060
Current Data Parameters
NAME 29.09.2011
EXPNO 8
PROCNO 1
F2 - Acquisition Parameters
Date_ 20110929
Time 22.28
INSTRUM spect
PROBHD 5 mm DUL 13C-1
PULPROG zg30
TD 32768
SOLVENT CDCl3
NS 18
DS 0
SWH 6410.256 Hz
FIDRES 0.195625 Hz
AQ 2.5559540 sec
RG 71.8
DW 78.000 usec
DE 6.00 usec
TE 300.0 K
D1 2.00000000 sec
TD0 1
======== CHANNEL f1 ========
NUC1 1H
P1 10.40 usec
PL1 -2.40 dB
SFO1 400.1528010 MHz
F2 - Processing parameters
SI 16384
SF 400.1500161 MHz
WDW EM
SSB 0
LB 0.00 Hz
GB 0
PC 1.00
DV-1961
OMe
Ph
H
O
6e
9
8
7
6
5
4
3
2
1
ppm
2.113
0.993
1.016
2.181
1.165
3.237
1.005
3.000

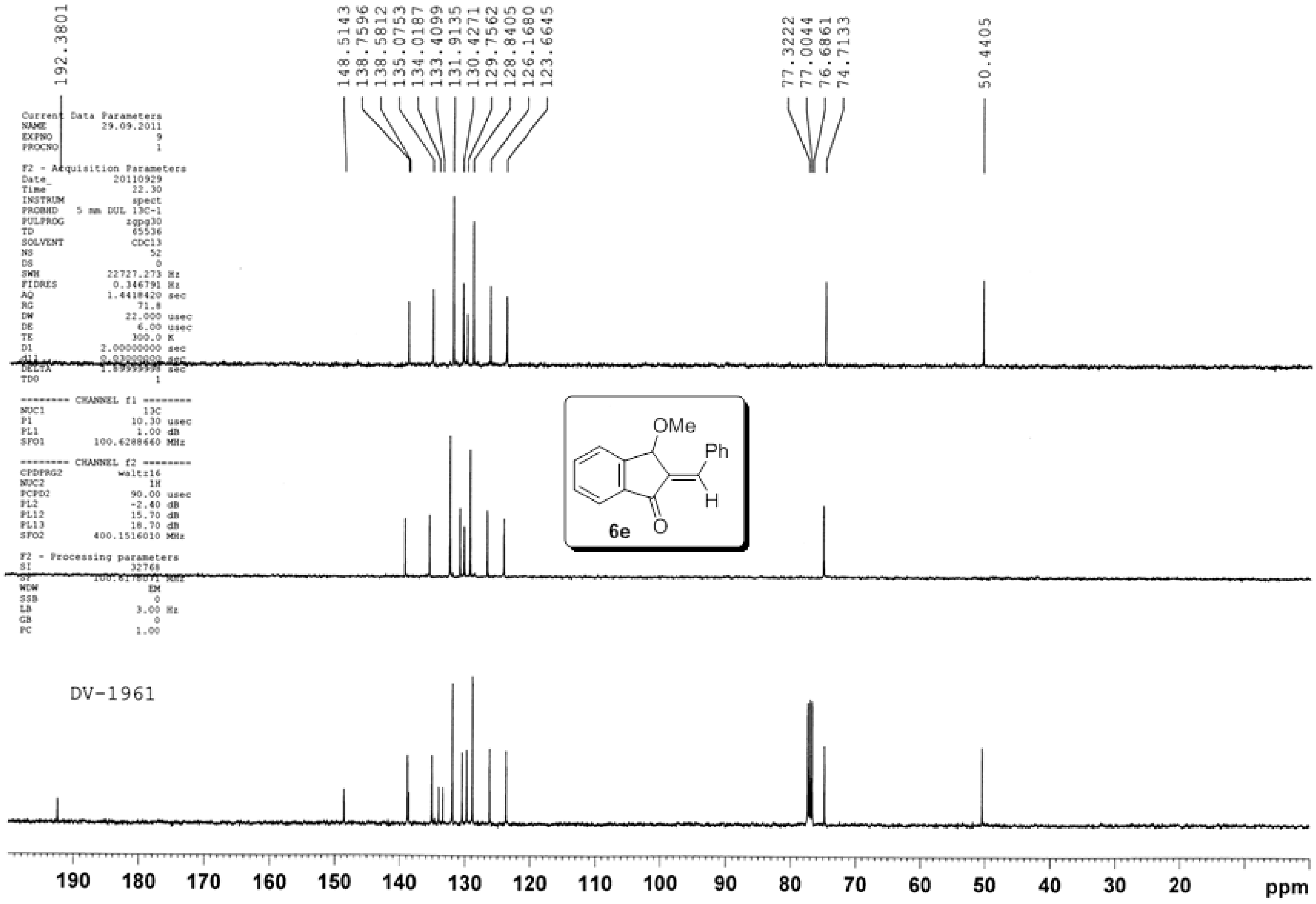
192.3801
148.5143
138.7596
138.5812
135.0753
134.0187
133.4099
131.9135
130.4271
129.7562
128.8405
126.1680
123.6645
77.3222
77.0044
76.6861
74.7133
50.4405
Current Data Parameters
NAME 29.09.2011
EXPNO 9
PROCNO 1
F2 - Acquisition Parameters
Date_ 20110929
Time 22.30
INSTRUM spect
PROBHD 5 mm DUL 13C-1
PULPROG zgpg30
TD 65536
SOLVENT CDCl3
NS 52
DS 0
SWH 22727.273 Hz
FIDRES 0.346791 Hz
AQ 1.4418420 sec
RG 71.8
DW 22.000 usec
DE 6.00 usec
TE 300.0 K
D1 2.00000000 sec
TD0 1
======== CHANNEL f1 ========
NUC1 13C
P1 10.30 usec
PL1 1.00 dB
SFO1 100.6288660 MHz
======== CHANNEL f2 ========
CPDPRG2 waltz16
NUC2 1H
PCPD2 90.00 usec
PL2 -2.40 dB
PL12 15.70 dB
PL13 18.70 dB
SFO2 400.1516010 MHz
F2 - Processing parameters
SI 32768
WDW EM
SSB 0
LB 3.00 Hz
GB 0
PC 1.00
OMe
Ph
H
O
6e
DV-1961
190 180 170 160 150 140 130 120 110 100 90 80 70 60 50 40 30 20 ppm

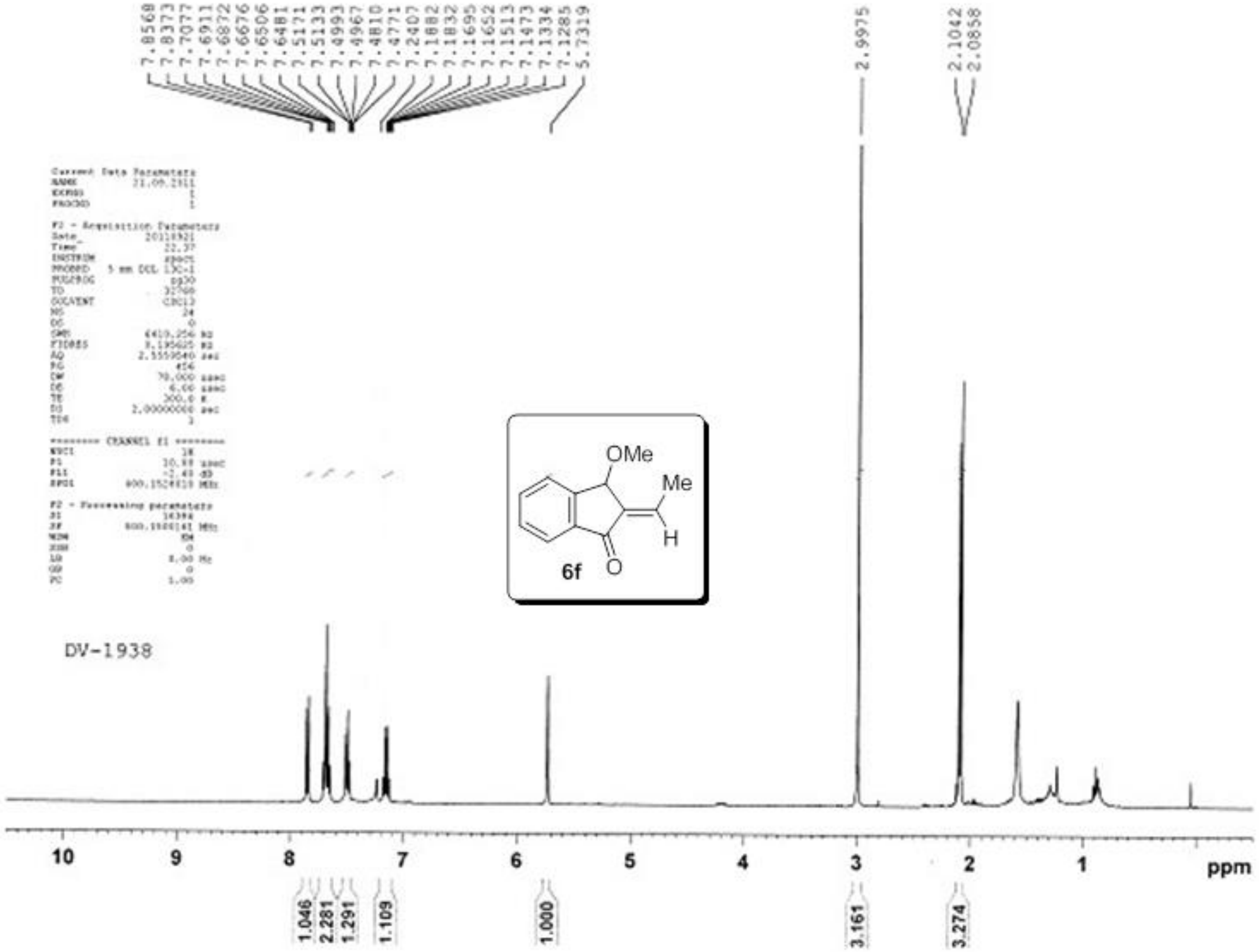

DV-1938
OMe
Me
H
O
6f
ppm

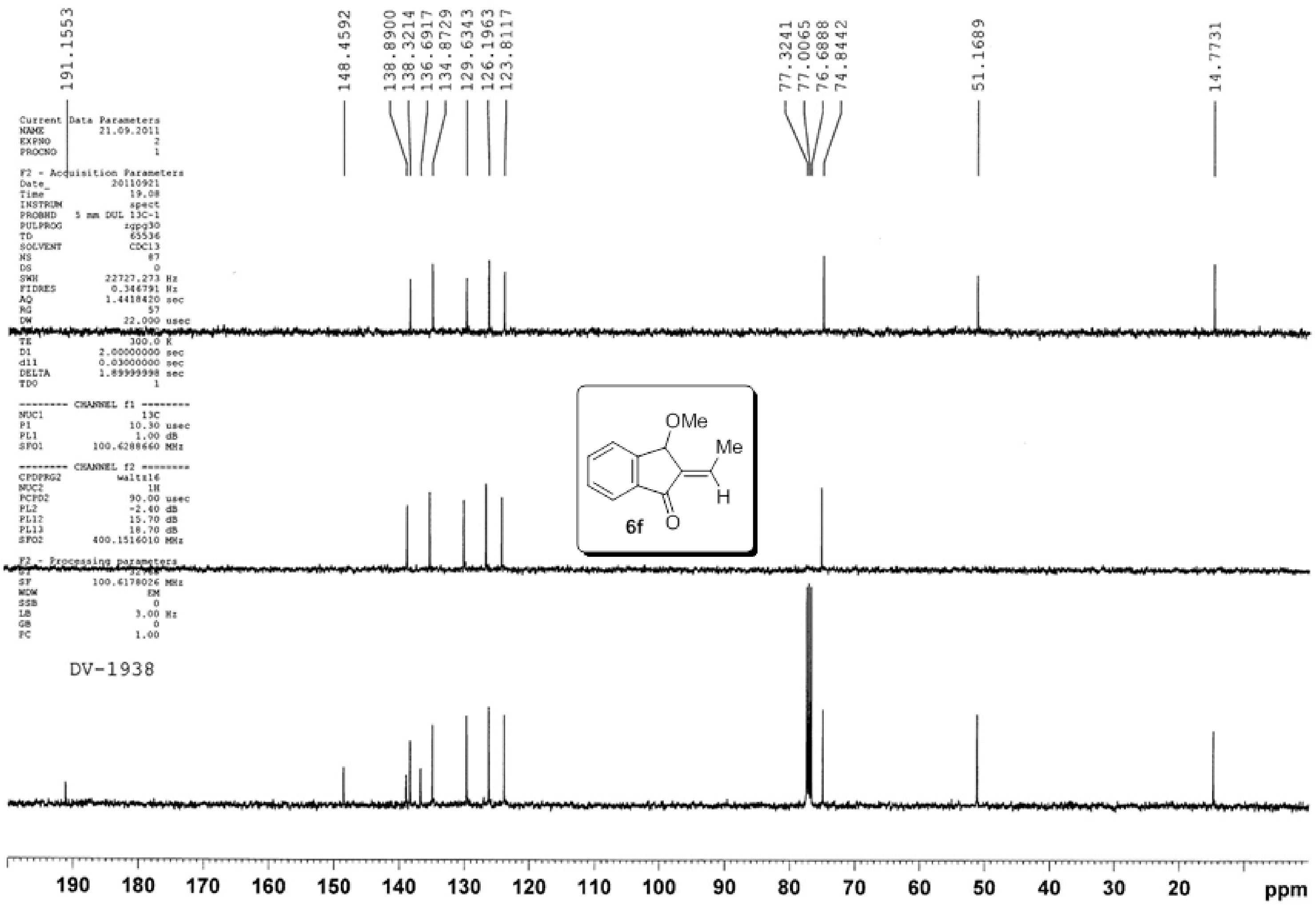

191.1553
148.4592
138.8900
138.3214
136.6917
134.8729
129.6343
126.1963
123.8117
77.3241
77.0065
76.6888
74.8442
51.1689
14.7731
Current Data Parameters
NAME 21.09.2011
EXPNO 2
PROCNO 1
F2 - Acquisition Parameters
Date_ 20110921
Time 19.08
INSTRUM spect
PROBHD 5 mm DUL 13C-1
PULPROG zgpg30
TD 65536
SOLVENT CDCl3
NS 87
DS 0
SWH 22727.273 Hz
FIDRES 0.346791 Hz
AQ 1.4418420 sec
RG 57
DW 22.000 usec
TE 300.0 K
D1 2.00000000 sec
d11 0.03000000 sec
DELTA 1.89999998 sec
TD0 1
======== CHANNEL f1 ========
NUC1 13C
P1 10.30 usec
PL1 1.00 dB
SFO1 100.6288660 MHz
======== CHANNEL f2 ========
CPDPRG2 waltz16
NUC2 1H
PCPD2 90.00 usec
PL2 -2.40 dB
PL12 15.70 dB
PL13 18.70 dB
SFO2 400.1516010 MHz
F2 - Processing parameters
SF 100.6178026 MHz
WDW EM
SSB 0
LB 3.00 Hz
GB 0
PC 1.00
DV-1938
OMe
Me
H
O
6f
190 180 170 160 150 140 130 120 110 100 90 80 70 60 50 40 30 20 ppm

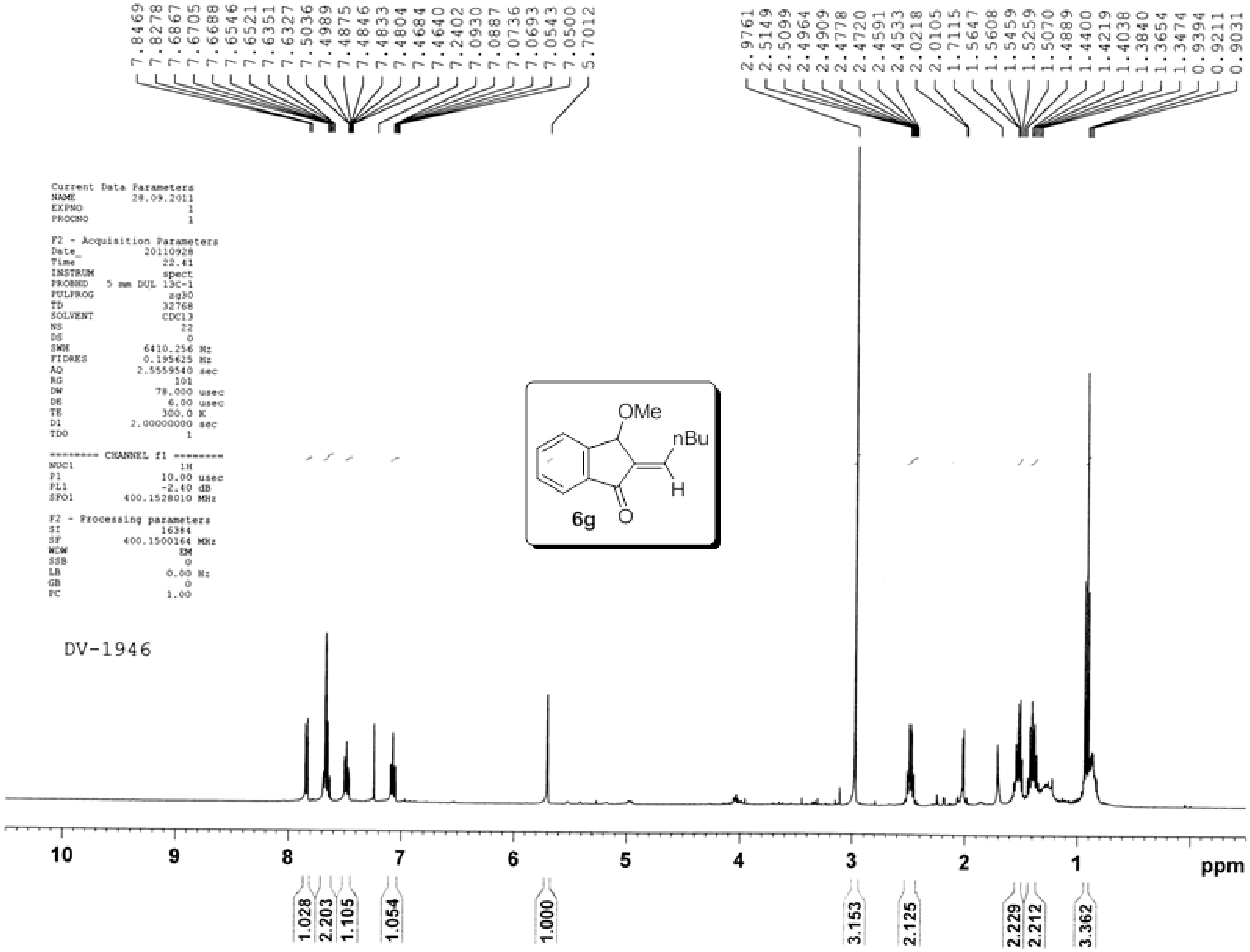

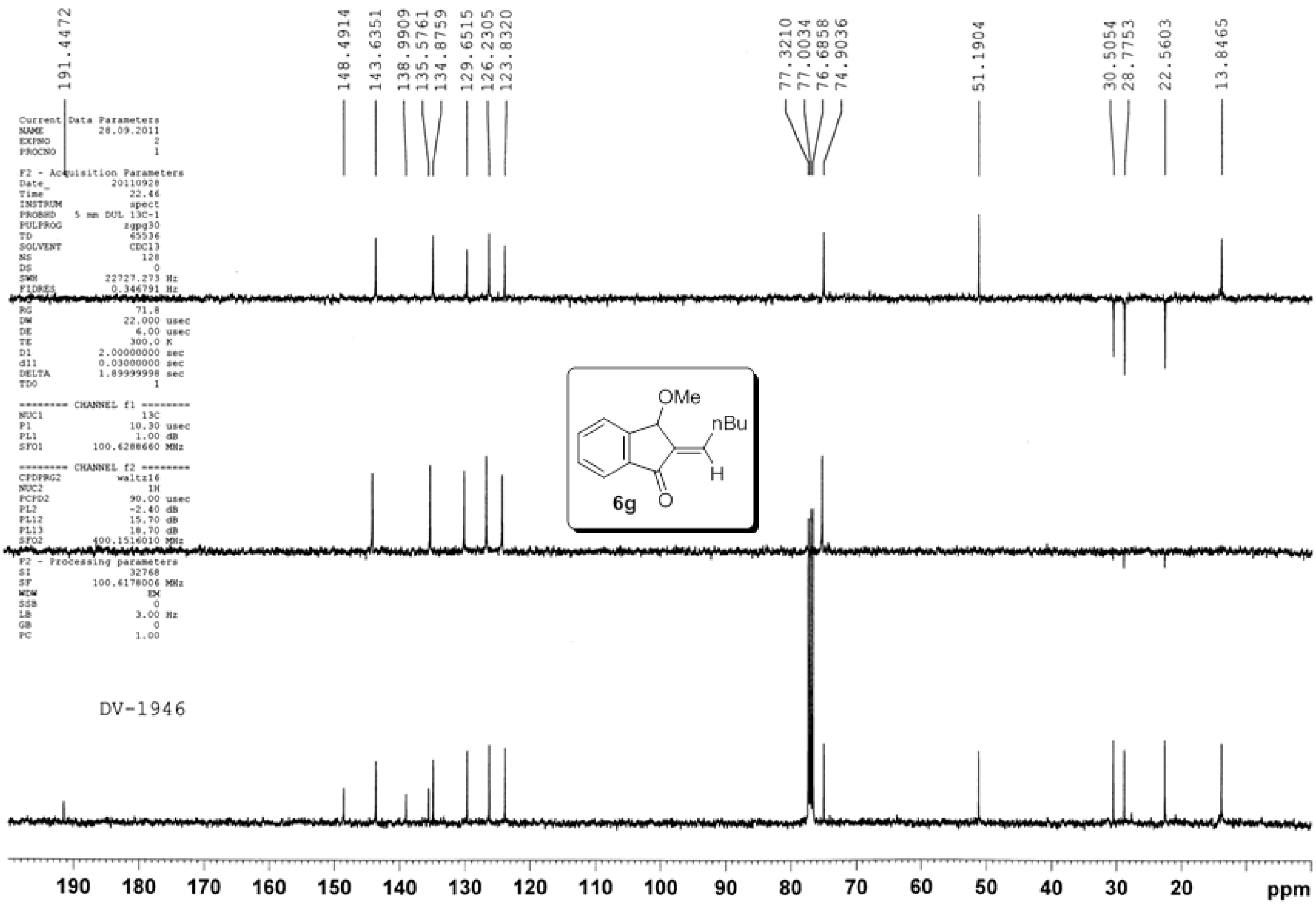

191.4472
148.4914
143.6351
138.9909
135.5761
134.8759
129.6515
126.2305
123.8320
77.3210
77.0034
76.6858
74.9036
51.1904
30.5054
28.7753
22.5603
13.8465
OMe
nBu
H
O
6g
DV-1946
190 180 170 160 150 140 130 120 110 100 90 80 70 60 50 40 30 20 ppm

^{1}H NOE spectra of compounds 4c, 4e, and 4f

NOE of compound 4c

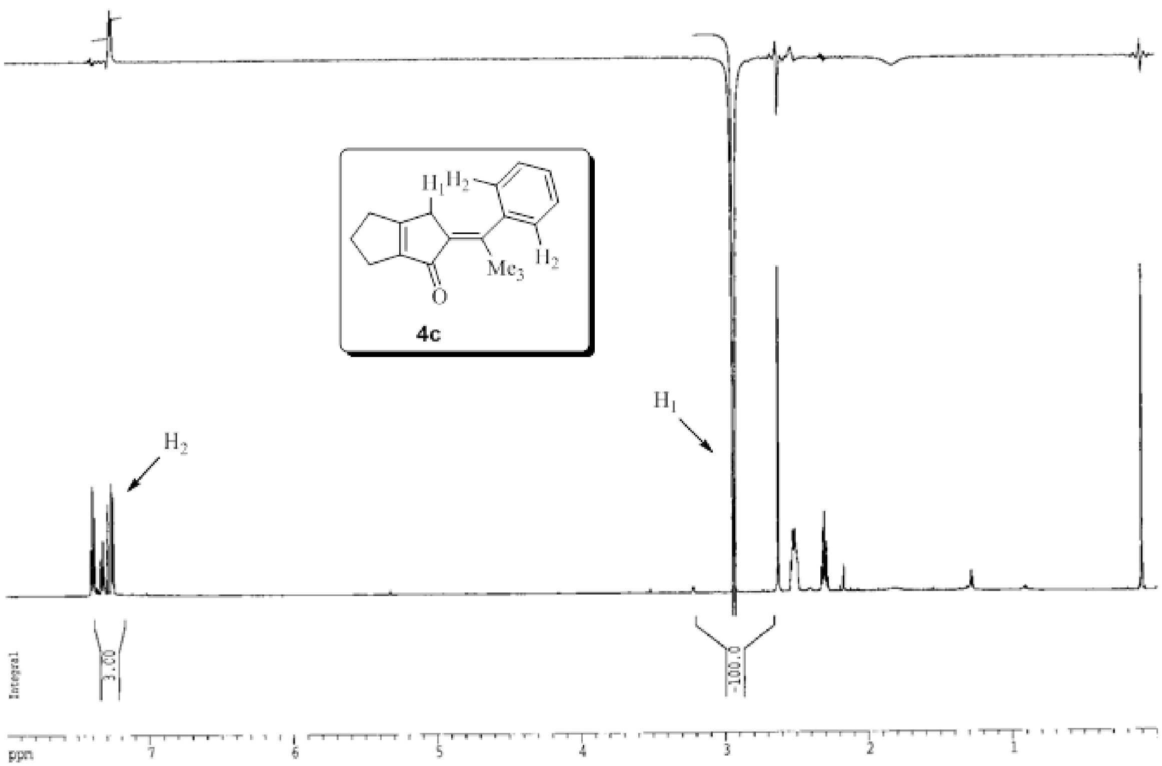

NOE of compound 4c

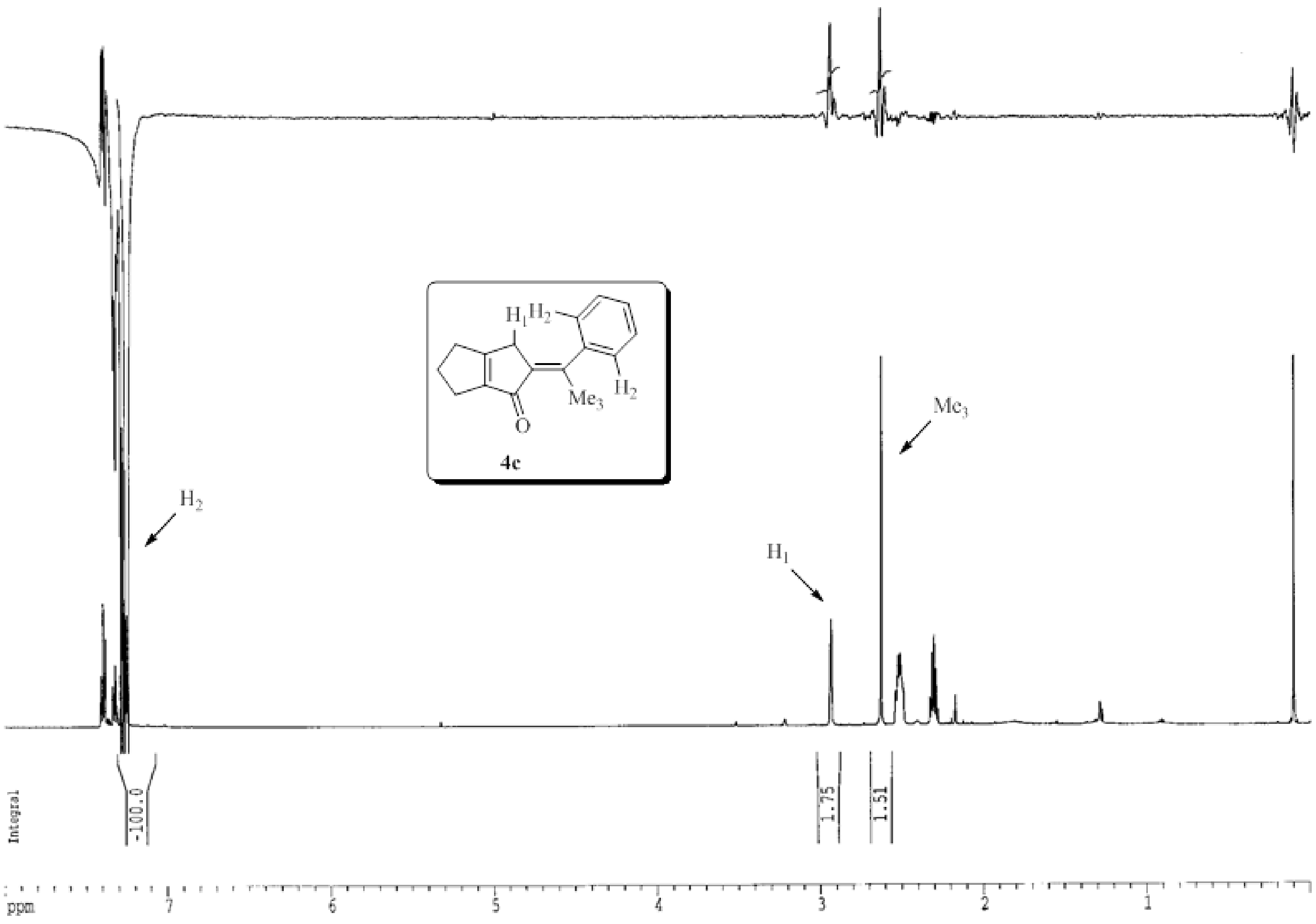

NOE of compound 4c

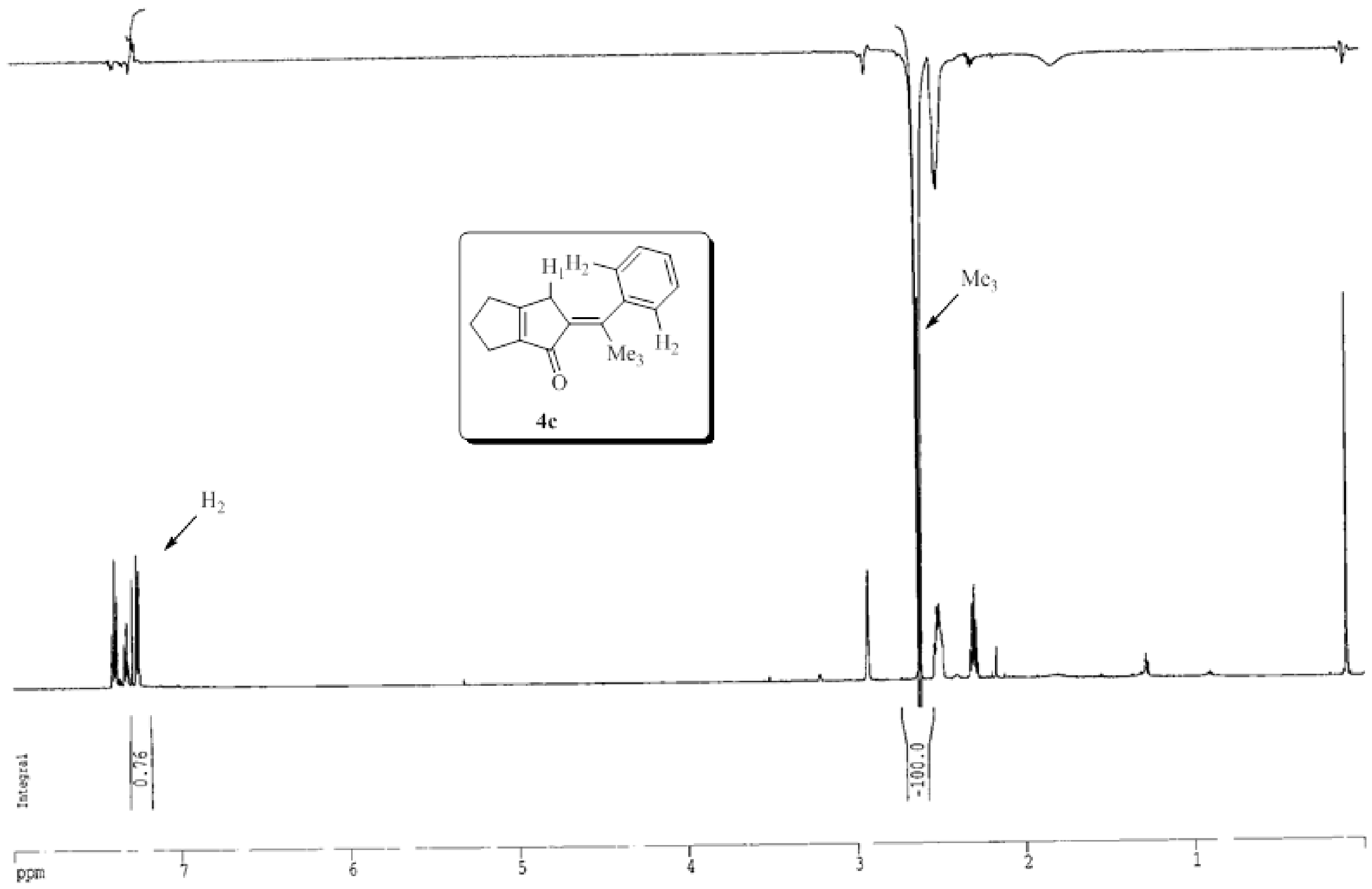

NOE of compound 4e

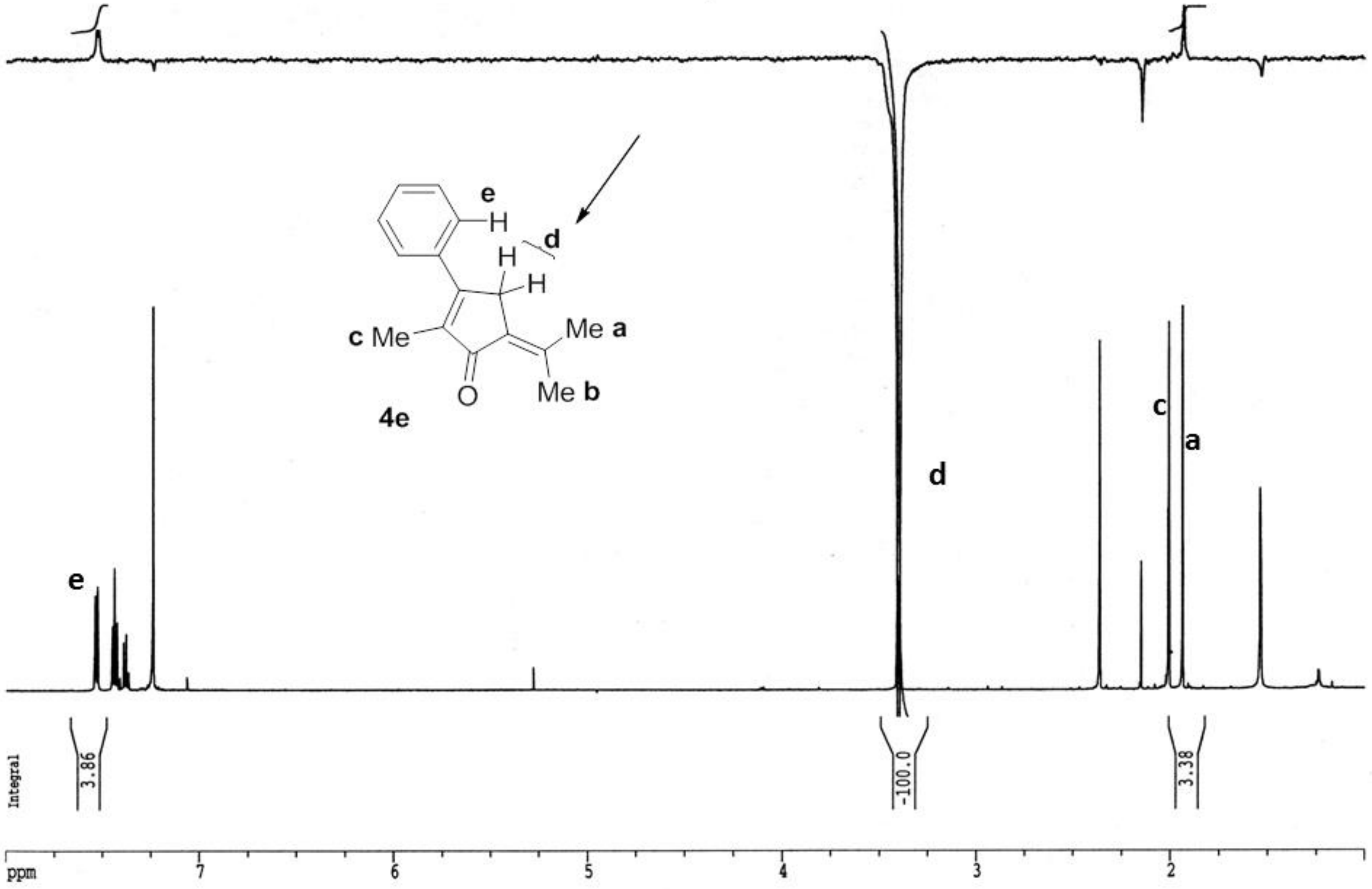

NOE of compound 4e

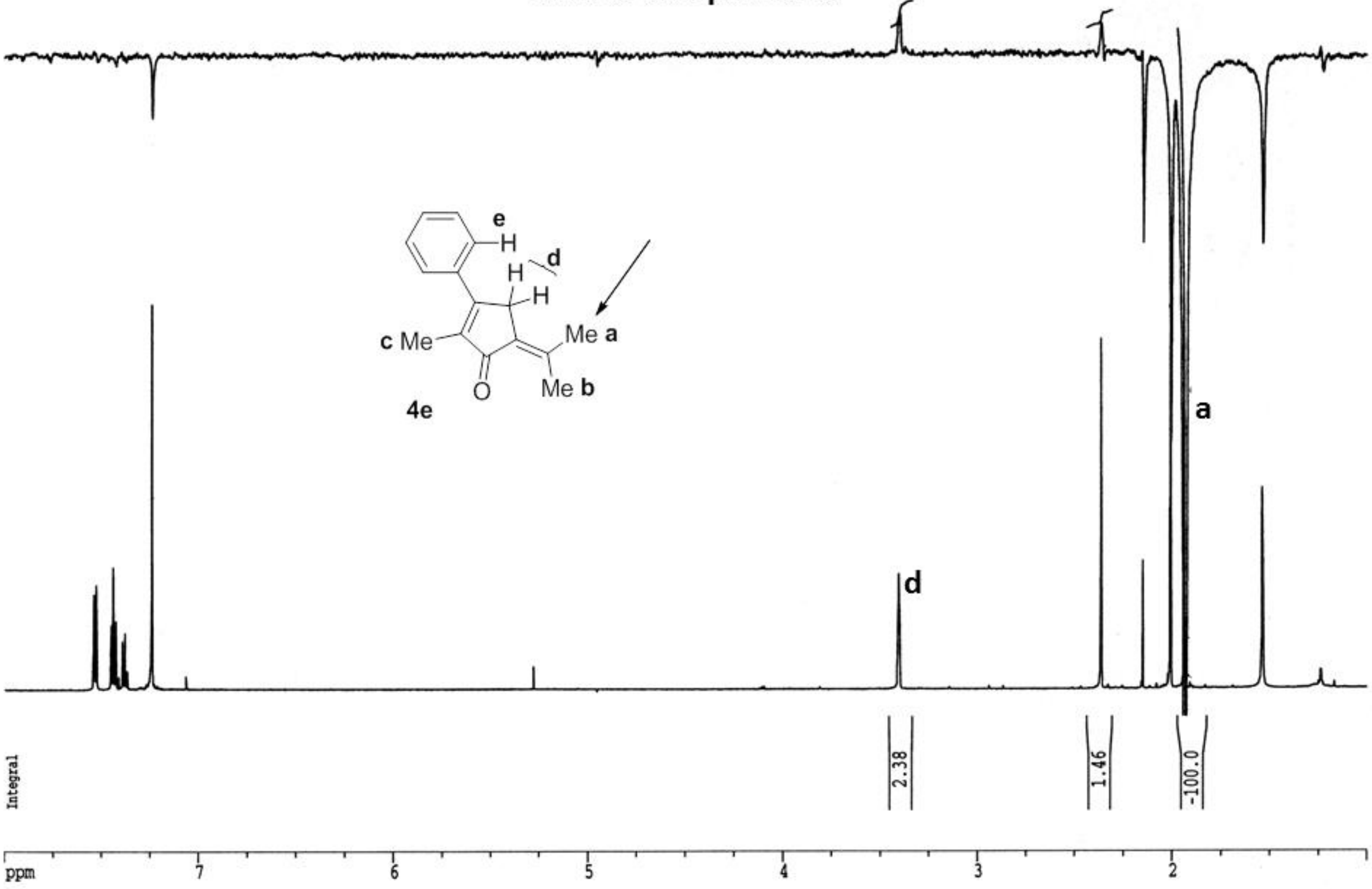

NOE of compound 4e

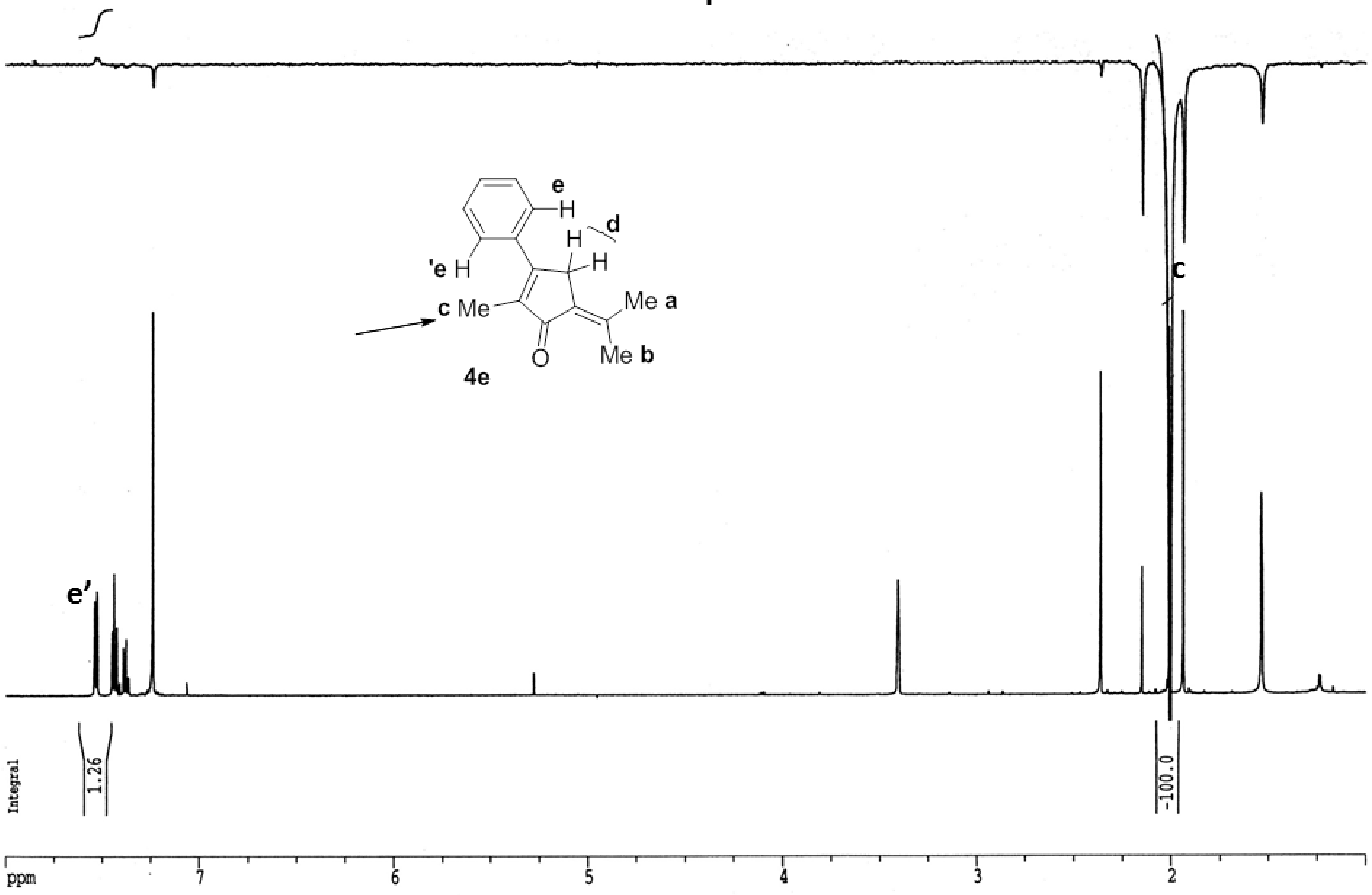

NOE of compound 4e

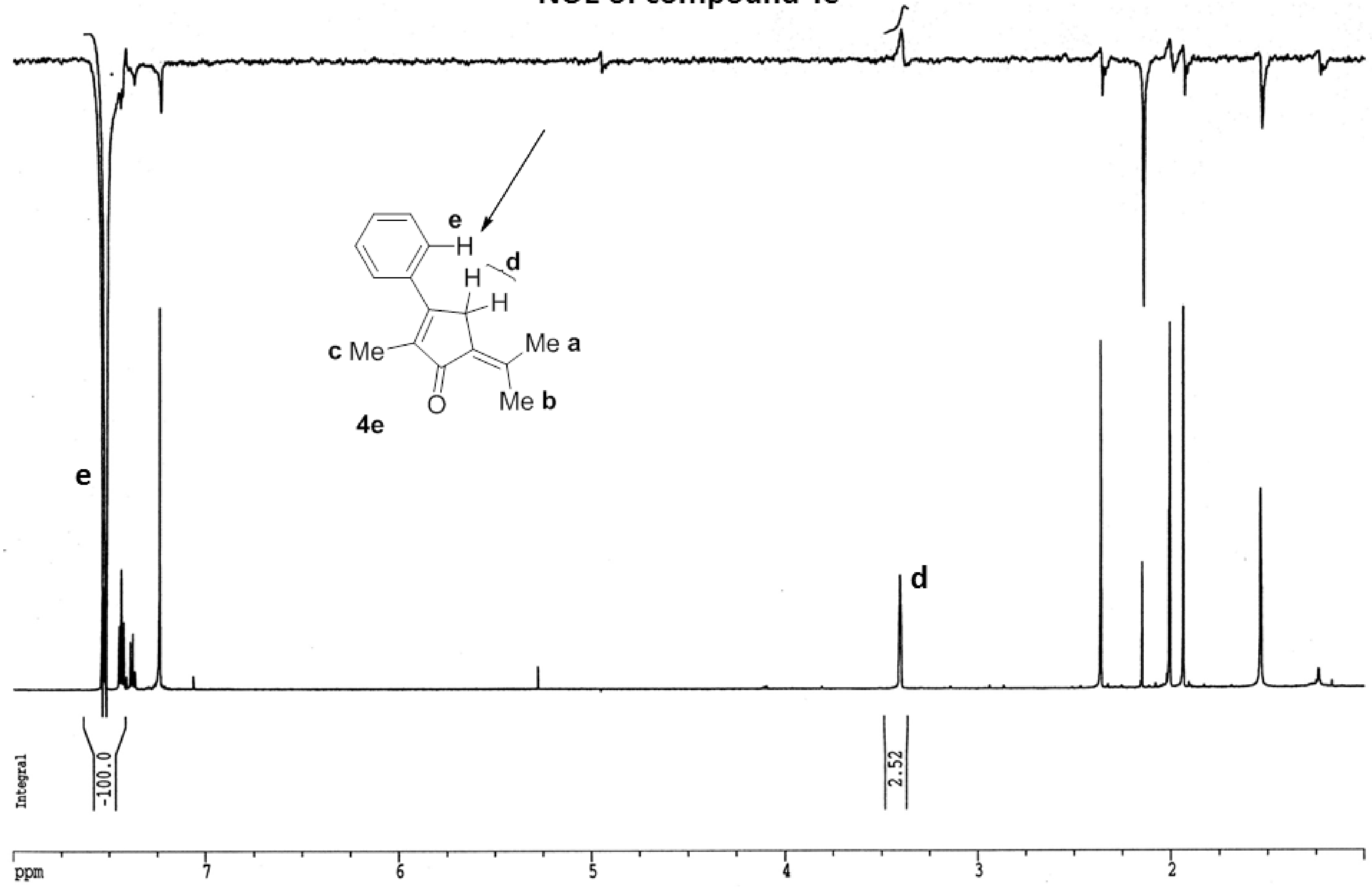

NOE of compound 4f

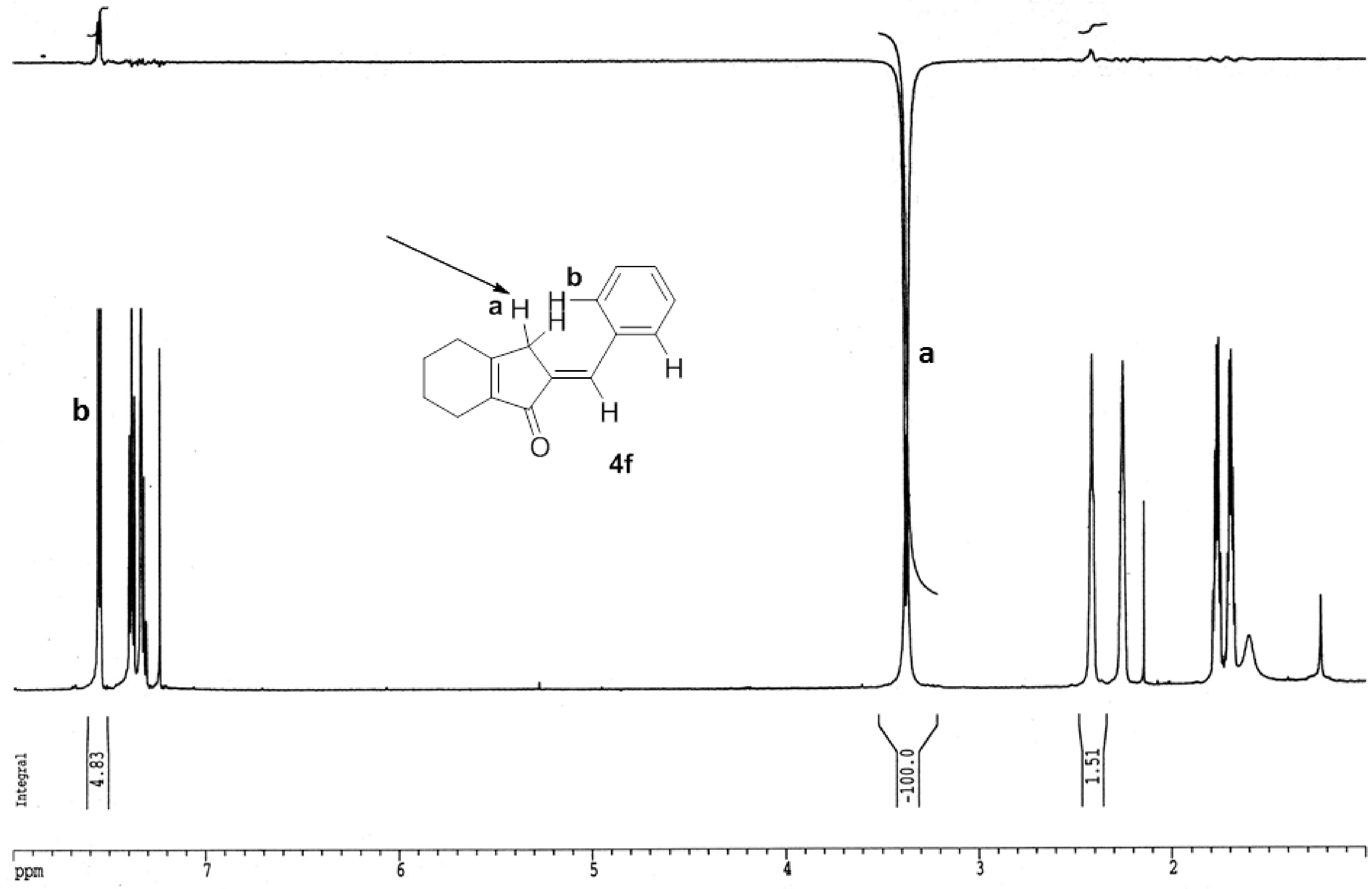

NOE of compound 4f

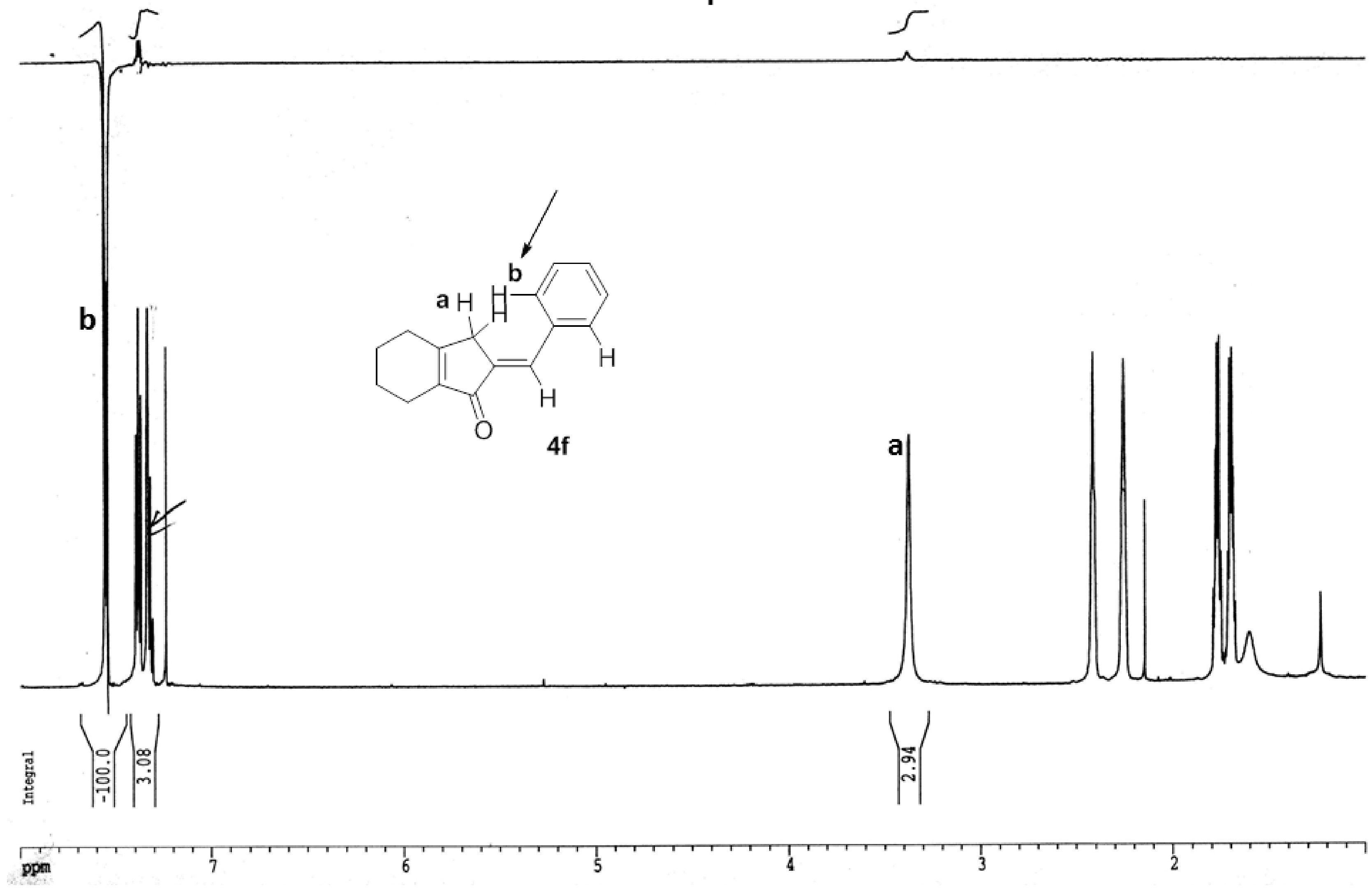

X-ray structure of compound 6a

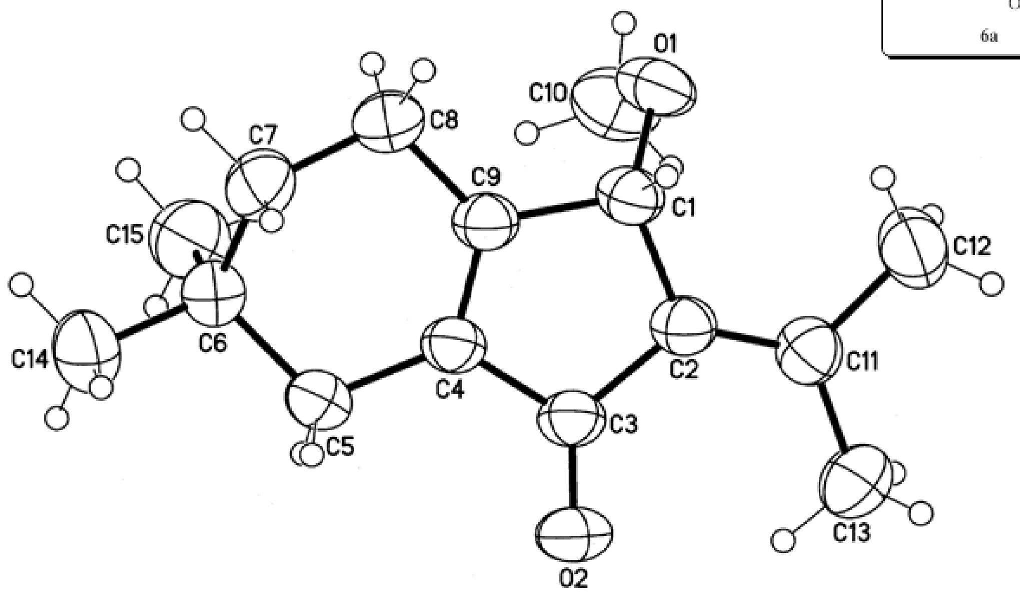
O1
C10
C8
C7
C9
C1
C15
C12
C6
C2
C11
C14
C4
C3
C5
C13
O2

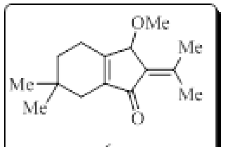
OMe
Me
Me
Me
Me
O
6a

Table 1. Crystal data and structure refinement for 08NV24_0m.

Identification code	08nv24_0m	
Empirical formula	C15 H22 O2	
Formula weight	234.33	
Temperature	296(2) K	
Wavelength	0.71073 Å	
Crystal system	Triclinic	
Space group	*P*-1	
Unit cell dimensions	a = 8.0947(3) Å	α= 79.573(2)°.
	b = 9.4471(3) Å	β= 87.361(2)°.
	c = 9.5269(3) Å	γ = 72.261(2)°.
Volume	682.41(4) $Å^3$	
Z	2	
Density (calculated)	1.140 Mg/m^3	
Absorption coefficient	0.074 mm^{-1}	
F(000)	256	
Crystal size	0.30 x 0.30 x 0.30 mm^3	
Theta range for data collection	2.17 to 28.32°.	
Index ranges	-10≤h≤10, -12≤k≤10, -11≤l≤12	
Reflections collected	12717	
Independent reflections	3373 [R(int) = 0.0189]	
Completeness to theta = 28.32°	99.3 %	
Absorption correction	Empirical	
Max. and min. transmission	0.7454 and 0.7120	
Refinement method	Full-matrix least-squares on F^2	
Data / restraints / parameters	3373 / 0 / 159	

Goodness-of-fit on F^2	1.071
Final R indices [I>2sigma(I)]	R1 = 0.0464, wR2 = 0.1411
R indices (all data)	R1 = 0.0580, wR2 = 0.1523
Largest diff. peak and hole	0.220 and -0.146 e.Å^{-3}

Table 2. Atomic coordinates (x 10^4) and equivalent isotropic displacement parameters ($Å^2$x 10^3) for 08NV24_0m. U(eq) is defined as one third of the trace of the orthogonalized U^{ij} tensor.

	x	y	z	U(eq)
C(4)	7020(1)	2004(1)	8695(1)	43(1)
C(1)	3985(2)	2370(1)	8785(1)	46(1)
C(3)	6592(2)	1712(1)	10219(1)	45(1)
C(9)	5578(2)	2369(1)	7899(1)	44(1)
C(2)	4680(2)	1980(1)	10305(1)	44(1)
C(5)	8792(2)	1899(2)	8124(1)	50(1)
C(6)	8748(2)	2636(2)	6542(1)	52(1)
C(11)	3732(2)	1883(2)	11493(1)	50(1)
C(8)	5564(2)	2737(2)	6312(1)	58(1)
C(7)	7398(2)	2228(2)	5736(1)	61(1)
C(13)	4487(2)	1514(2)	12972(1)	64(1)
C(12)	1806(2)	2175(2)	11428(2)	69(1)
C(15)	8273(2)	4342(2)	6431(2)	74(1)
C(14)	10536(2)	2058(2)	5895(2)	76(1)
C(10)	2965(2)	5043(2)	8723(2)	73(1)
O(1)	2604(1)	3748(1)	8429(1)	60(1)
O(2)	7643(1)	1320(1)	11202(1)	66(1)

Table 3. Bond lengths [Å] and angles [°] for 08NV24_0m.

C(4)-C(9)	1.3415(16)
C(4)-C(3)	1.4734(16)
C(4)-C(5)	1.4918(16)
C(1)-O(1)	1.4307(15)
C(1)-C(9)	1.5080(17)
C(1)-C(2)	1.5183(16)
C(1)-H(1)	0.9800
C(3)-O(2)	1.2223(14)
C(3)-C(2)	1.4909(16)
C(9)-C(8)	1.4879(17)
C(2)-C(11)	1.3435(18)
C(5)-C(6)	1.5389(17)
C(5)-H(5A)	0.9700
C(5)-H(5B)	0.9700
C(6)-C(15)	1.524(2)
C(6)-C(14)	1.5268(19)
C(6)-C(7)	1.5404(19)
C(11)-C(13)	1.5004(18)
C(11)-C(12)	1.5006(19)
C(8)-C(7)	1.524(2)
C(8)-H(8A)	0.9700
C(8)-H(8B)	0.9700
C(7)-H(7A)	0.9700
C(7)-H(7B)	0.9700
C(13)-H(13A)	0.9600

C(13)-H(13B)	0.9600
C(13)-H(13C)	0.9600
C(12)-H(12A)	0.9600
C(12)-H(12B)	0.9600
C(12)-H(12C)	0.9600
C(15)-H(15A)	0.9600
C(15)-H(15B)	0.9600
C(15)-H(15C)	0.9600
C(14)-H(14A)	0.9600
C(14)-H(14B)	0.9600
C(14)-H(14C)	0.9600
C(10)-O(1)	1.417(2)
C(10)-H(10A)	0.9600
C(10)-H(10B)	0.9600
C(10)-H(10C)	0.9600
C(9)-C(4)-C(3)	109.78(11)
C(9)-C(4)-C(5)	125.11(11)
C(3)-C(4)-C(5)	125.11(10)
O(1)-C(1)-C(9)	112.35(10)
O(1)-C(1)-C(2)	116.54(10)
C(9)-C(1)-C(2)	103.15(9)
O(1)-C(1)-H(1)	108.1
C(9)-C(1)-H(1)	108.1
C(2)-C(1)-H(1)	108.1
O(2)-C(3)-C(4)	124.99(11)
O(2)-C(3)-C(2)	127.94(11)

C(4)-C(3)-C(2)	107.07(9)
C(4)-C(9)-C(8)	123.24(12)
C(4)-C(9)-C(1)	112.71(10)
C(8)-C(9)-C(1)	124.04(10)
C(11)-C(2)-C(3)	127.07(11)
C(11)-C(2)-C(1)	125.72(11)
C(3)-C(2)-C(1)	107.22(10)
C(4)-C(5)-C(6)	112.14(10)
C(4)-C(5)-H(5A)	109.2
C(6)-C(5)-H(5A)	109.2
C(4)-C(5)-H(5B)	109.2
C(6)-C(5)-H(5B)	109.2
H(5A)-C(5)-H(5B)	107.9
C(15)-C(6)-C(14)	109.12(13)
C(15)-C(6)-C(5)	109.15(12)
C(14)-C(6)-C(5)	109.43(11)
C(15)-C(6)-C(7)	110.18(12)
C(14)-C(6)-C(7)	109.58(12)
C(5)-C(6)-C(7)	109.37(11)
C(2)-C(11)-C(13)	123.42(12)
C(2)-C(11)-C(12)	121.64(12)
C(13)-C(11)-C(12)	114.92(12)
C(9)-C(8)-C(7)	110.65(10)
C(9)-C(8)-H(8A)	109.5
C(7)-C(8)-H(8A)	109.5
C(9)-C(8)-H(8B)	109.5
C(7)-C(8)-H(8B)	109.5

H(8A)-C(8)-H(8B)	108.1
C(8)-C(7)-C(6)	113.82(11)
C(8)-C(7)-H(7A)	108.8
C(6)-C(7)-H(7A)	108.8
C(8)-C(7)-H(7B)	108.8
C(6)-C(7)-H(7B)	108.8
H(7A)-C(7)-H(7B)	107.7
C(11)-C(13)-H(13A)	109.5
C(11)-C(13)-H(13B)	109.5
H(13A)-C(13)-H(13B)	109.5
C(11)-C(13)-H(13C)	109.5
H(13A)-C(13)-H(13C)	109.5
H(13B)-C(13)-H(13C)	109.5
C(11)-C(12)-H(12A)	109.5
C(11)-C(12)-H(12B)	109.5
H(12A)-C(12)-H(12B)	109.5
C(11)-C(12)-H(12C)	109.5
H(12A)-C(12)-H(12C)	109.5
H(12B)-C(12)-H(12C)	109.5
C(6)-C(15)-H(15A)	109.5
C(6)-C(15)-H(15B)	109.5
H(15A)-C(15)-H(15B)	109.5
C(6)-C(15)-H(15C)	109.5
H(15A)-C(15)-H(15C)	109.5
H(15B)-C(15)-H(15C)	109.5
C(6)-C(14)-H(14A)	109.5
C(6)-C(14)-H(14B)	109.5

H(14A)-C(14)-H(14B)	109.5
C(6)-C(14)-H(14C)	109.5
H(14A)-C(14)-H(14C)	109.5
H(14B)-C(14)-H(14C)	109.5
O(1)-C(10)-H(10A)	109.5
O(1)-C(10)-H(10B)	109.5
H(10A)-C(10)-H(10B)	109.5
O(1)-C(10)-H(10C)	109.5
H(10A)-C(10)-H(10C)	109.5
H(10B)-C(10)-H(10C)	109.5
C(10)-O(1)-C(1)	114.56(10)

Symmetry transformations used to generate equivalent atoms:

Table 4. Anisotropic displacement parameters ($Å^2$x 10^3) for 08NV24_0m. The anisotropic displacement factor exponent takes the form: $-2\pi^2[h^2 a^{*2}U^{11} + ... + 2 h k a^* b^* U^{12}]$

	U^{11}	U^{22}	U^{33}	U^{23}	U^{13}	U^{12}
C(4)	38(1)	44(1)	42(1)	-6(1)	-4(1)	-6(1)
C(1)	40(1)	52(1)	46(1)	-10(1)	-5(1)	-11(1)
C(3)	42(1)	44(1)	42(1)	-5(1)	-5(1)	-5(1)
C(9)	42(1)	48(1)	41(1)	-8(1)	-5(1)	-10(1)
C(2)	42(1)	46(1)	42(1)	-9(1)	-3(1)	-10(1)
C(5)	39(1)	59(1)	49(1)	-6(1)	-3(1)	-9(1)

C(6)	47(1)	61(1)	48(1)	-6(1)	2(1)	-17(1)
C(11)	49(1)	53(1)	48(1)	-14(1)	3(1)	-15(1)
C(8)	55(1)	80(1)	41(1)	-6(1)	-8(1)	-23(1)
C(7)	62(1)	83(1)	43(1)	-13(1)	2(1)	-27(1)
C(13)	69(1)	78(1)	44(1)	-12(1)	3(1)	-20(1)
C(12)	53(1)	92(1)	66(1)	-22(1)	10(1)	-27(1)
C(15)	72(1)	63(1)	85(1)	2(1)	-4(1)	-26(1)
C(14)	56(1)	103(1)	66(1)	-16(1)	14(1)	-23(1)
C(10)	60(1)	55(1)	90(1)	-10(1)	-10(1)	2(1)
O(1)	39(1)	67(1)	66(1)	-10(1)	-13(1)	-4(1)
O(2)	49(1)	89(1)	46(1)	1(1)	-13(1)	-7(1)

Table 5. Hydrogen coordinates (x 10^4) and isotropic displacement parameters (Å^{2}x 10 ^{3}) for 08NV24_0m.

	x	y	z	U(eq)
H(1)	3580	1542	8603	55
H(5A)	9476	845	8229	61
H(5B)	9353	2391	8678	61
H(8A)	5068	3818	6008	70
H(8B)	4847	2240	5928	70
H(7A)	7390	2685	4738	74
H(7B)	7749	1142	5788	74
H(13A)	5714	1365	12917	97
H(13B)	4276	610	13474	97
H(13C)	3954	2333	13472	97
H(12A)	1226	3171	11613	103
H(12B)	1479	1446	12131	103
H(12C)	1481	2093	10496	103
H(15A)	7184	4703	6893	111
H(15B)	8177	4812	5444	111
H(15C)	9158	4587	6887	111
H(14A)	11373	2325	6390	113
H(14B)	10505	2506	4904	113
H(14C)	10855	980	5984	113
H(10A)	3019	4986	9737	109
H(10B)	2063	5933	8321	109

H(10C)	4057	5087	8311	109
